# The TUBA1B–G3BP2 Axis Sustains p65 Transcriptional Activity to Promote Hepatocellular Carcinoma Progression

**DOI:** 10.1002/advs.77502

**Published:** 2026-09-06

**Authors:** Fen Lin, Hechun Lin, Junming Yu, Chao Ge, Lin Mao, Saihua Zhang, Yizi Jin, Yunyu Wu, Taoyang Chen, Jinjun Li, Hong Li

**Affiliations:** ^1^ State Key Laboratory of Systems Medicine for Cancer Shanghai Cancer Institute Renji Hospital Shanghai Jiao Tong University School of Medicine Shanghai China; ^2^ Department of Pathology Qidong Liver Cancer Institute Qidong People's Hospital Affiliated Qidong Hospital of Nantong University Qidong Jiangsu China

**Keywords:** G3BP2, hepatocellular carcinoma, p65 activation, protein stability, TUBA1B

## Abstract

Hepatocellular carcinoma (HCC) is a leading cause of cancer mortality, yet mechanisms sustaining malignant progression remain incompletely defined. Integrative analyses across multiple HCC cohorts identified TUBA1B as one of the most consistently upregulated tubulin isotypes whose high expression associates with poor prognosis and metastasis. In hydrodynamic tail‐vein injection (HTVi) models, co‐expression of *Tuba1b* with *myr‐AKT* enhanced liver tumor growth, supporting a tumor‐promoting role for *Tuba1b* in vivo. TUBA1B knockdown suppressed HCC cell proliferation, migration, and invasion in vitro and reduced tumor growth and metastasis in orthotopic models. HTVi‐mediated delivery of sg*Tuba1b* suppressed hepatocarcinogenesis in *NRasV12/myr‐AKT*– and *MYC/sg‐p53* HCC models and prolonged survival in the former, whereas sg*Tuba1b* delivery to normal livers caused no detectable histological abnormalities. Mechanistically, TUBA1B bound and stabilized G3BP2 by restraining TRIM25‐dependent K48‐linked ubiquitination, thereby promoting G3BP2–IκBα association and sustaining basal NF‐κB/p65 transcriptional activity; enforced G3BP2 expression rescued the proliferative and metastatic defects caused by TUBA1B loss. Clinically, combined upregulation of TUBA1B and G3BP2 with elevated p65 activity delineated a high‐risk HCC subgroup with the poorest outcomes. Collectively, these findings define a TUBA1B–G3BP2 stability axis that supports malignant phenotypes and highlight a potential therapeutic vulnerability in HCC.

## Graphical abstract

TUBA1B is consistently upregulated in hepatocellular carcinoma and sustains malignant progression by stabilizing G3BP2. By restraining TRIM25‐associated K48‐linked ubiquitination, TUBA1B preserves the G3BP2–IκBα complex and basal NF‐κB/p65 activity. Concurrent TUBA1B/G3BP2 upregulation identifies an aggressive HCC subgroup with enhanced p65 activation and poor prognosis, revealing a potential clinically relevant therapeutic vulnerability.

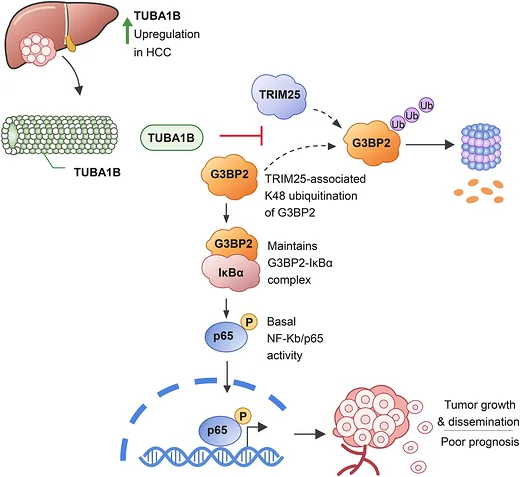

## 1 Introduction

Hepatocellular carcinoma remains a leading cause of cancer‐related mortality worldwide and is characterized by aggressive progression, high recurrence rates, and limited therapeutic options [1, 2]. A defining feature of advanced HCC is that tumor cells can maintain stable oncogenic states that support proliferation, survival, and metastatic competence even in the absence of persistent external stimuli [3]. However, how such malignant states are actively maintained at the cellular level remains incompletely understood.

Cytoskeletal organization has traditionally been viewed as a structural determinant of cell shape, division, and intracellular transport [4, 5]. However, emerging evidence suggests that cytoskeletal components can actively regulate signal transduction by organizing spatially confined signaling complexes and controlling the subcellular distribution of signaling molecules [6, 7, 8, 9]. In cancer cells, alterations in cytoskeletal architecture have been implicated not only in mechanical processes such as migration and invasion, but also in the stabilization of oncogenic signaling programs that enable tumor cells to adapt to metabolic and environmental stress [10, 11]. These observations raise the possibility that cytoskeletal structures may participate directly in the maintenance, rather than initiation, of malignant signaling states in hepatocellular carcinoma. Accordingly, microtubules may provide a plausible platform for such regulation by coordinating the spatial organization of signaling components.

Microtubules are dynamic polymers assembled from α/β‐tubulin heterodimers, and their behaviors are shaped by both post‐translational modifications (PTMs) of tubulin and the composition of tubulin isotypes [12, 13, 14, 15]. While PTMs such as acetylation and detyrosination have been widely studied in the context of microtubule stability and cancer progression [14, 15], comparatively less attention has been paid to the functional consequences of tubulin isotype diversity. Importantly, multiple tubulin isoforms are frequently upregulated in malignant tissues and have been linked to therapy resistance and poor prognosis [13, 16], supporting the notion that individual tubulin isotypes may exert non‐redundant functions beyond structural redundancy in cancer cells.

Through integrative analyses of genetically defined HCC mouse models and multiple independent human cohorts, we identified TUBA1B as the most consistently upregulated α‐tubulin isoform in HCC. Although elevated TUBA1B expression has been reported as a prognostic indicator [17], the biological basis for its consistent upregulation and its potential contribution to the maintenance of malignant cellular states remain unclear. In particular, whether TUBA1B exerts isoform‐specific, non‐canonical functions that support the maintenance of malignant cellular states has not been explored. Specifically, whether a single tubulin isoform can sustain basal oncogenic transcriptional output under steady‐state conditions remains unknown.

In this study, we uncover an unexpected isoform‐specific function of the α‐tubulin TUBA1B in sustaining malignant growth and metastatic progression in hepatocellular carcinoma. We demonstrate that TUBA1B restrains TRIM25‐dependent ubiquitination to stabilize G3BP2, thereby maintaining a G3BP2–IκBα control module that supports basal p65 transcriptional activity and downstream transcriptional output, under basal conditions and independently of overt canonical IKK activation. These findings define a TUBA1B–G3BP2 stability axis that links tubulin isoform dysregulation to the maintenance of aggressive oncogenic states in HCC.

## 2 Results

### 2.1 TUBA1B Is Consistently Upregulated and Exhibits Isoform‐Specific Tumor‐Promoting Activity in HCC

**FIGURE 1:**
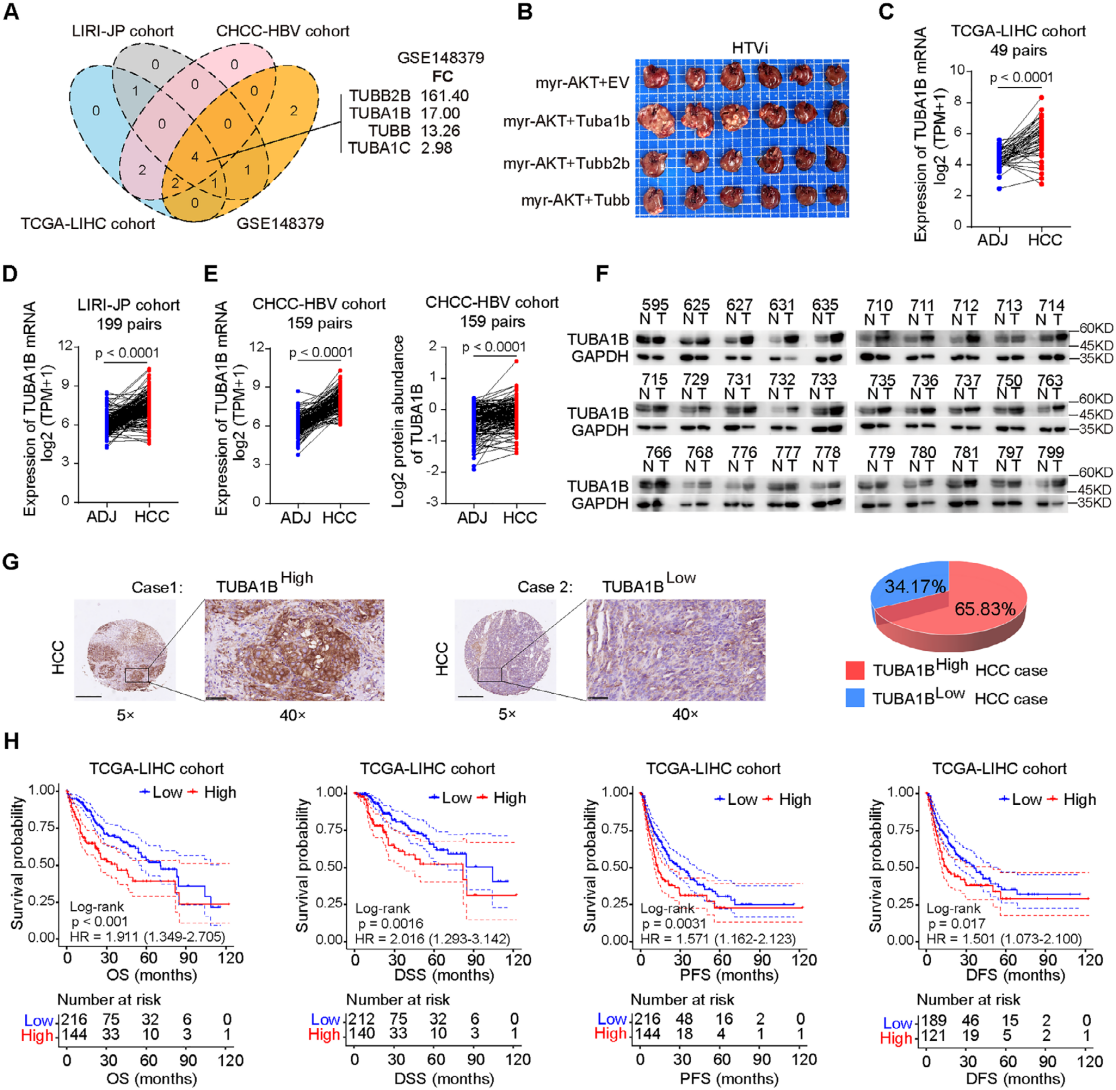
Multi‐cohort identification and functional specificity of TUBA1B in hepatocellular carcinoma. (A) Venn diagram illustrating the overlap of tubulin genes that met two screening criteria across TCGA‐LIHC, CHCC‐HBV, LIRI‐JP, and GSE148379: detectable tumor expression, defined as TPM > 3 [log2(TPM + 1) > 2], and >2‐fold upregulation in HCC tumor tissues compared with matched non‐tumor liver tissues or normal liver controls. For GSE148379, differential expression was assessed by comparing HCC samples from genetically engineered mouse models with normal mouse liver samples. The overlapping candidate genes were ranked according to their fold changes in GSE148379, in which Tubb2b displayed the highest fold increase. (B) Schematic of hydrodynamic tail vein injection (HTVi). Mice were co‐injected with *pT3‐myr‐AKT* and either *pT3‐EF1α* empty vector, *pT3‐EF1α‐Tuba1b*, *pT3‐EF1α‐Tubb2b*, or *pT3‐EF1α*‐*Tubb*, together with *SB* transposase. The total amount of injected DNA was kept consistent across groups (*n* = 6 per group). Liver tissues were collected 9 weeks post‐injection. *Tubb2b* and *Tubb* conferred minimal or no enhancement on liver tumor growth. (C, D) TUBA1B mRNA expression in paired tumor and adjacent non‐tumor tissues from the TCGA‐LIHC (*n* = 49) and LIRI‐JP (*n* = 199) cohorts. (E) TUBA1B mRNA and protein expression in 159 paired tumor and adjacent liver tissues from the CHCC‐HBV cohort. (F) Western blot analysis of TUBA1B protein levels in 30 paired HCC tumor tissues and matched adjacent non‐tumor liver tissues. (G) Representative immunohistochemical staining of TUBA1B in HCC. Pie chart shows the proportion of low (IHC score 1–2) and high (IHC score 3–4) TUBA1B expression in the institutional IHC cohort comprising 199 HCC patients. Scale bars: 500 µm (×5, left) and 50 µm (×40, right). (H) Kaplan–Meier survival analyses of overall survival (OS), disease‐specific survival (DSS), progression‐free survival (PFS), and disease‐free survival (DFS) in TCGA‐LIHC patients stratified by TUBA1B expression (mean cutoff). *p < 0.05, **p < 0.01, ***p < 0.001, ****p < 0.0001. Original blots can be found in Figure S17‐1.

To identify tubulin isoforms potentially involved in HCC progression, we analyzed transcriptomic data from GSE148379, which includes 11 mouse HCC sample groups generated under selected genetically defined oncogenic contexts [18], together with multiple independent human HCC cohorts [18, 19, 20]. GSE148379 was used as a cross‐species supportive dataset, and the analyzed mouse HCC sample groups are summarized in Table S6. Using a cross‐dataset screening strategy based on detectable tumor expression and tumor‐associated upregulation across TCGA‐LIHC, CHCC‐HBV, LIRI‐JP, and GSE148379, we identified *Tuba1b*, *Tubb2b*, *Tubb*, and *Tuba1c* as commonly upregulated candidates (Figures 1A and S1). The resulting candidates were ranked by their fold changes in GSE148379, and *Tuba1b, Tubb2b*, and *Tubb* were prioritized for HTVi‐based in vivo testing. In the HTVi‐based comparison, control mice received *myr‐AKT* plus empty vector, whereas experimental groups received *myr‐AKT* plus *Tuba1b, Tubb2b*, or *Tubb* expression plasmids. *Tuba1b* most potently enhanced liver tumor growth. In contrast, despite showing the greatest fold‐change upregulation in the cross‐species screen, *Tubb2b* conferred minimal or no enhancement, indicating that expression magnitude does not necessarily predict tumor‐promoting potency in vivo (Figure 1A,B). Across separately analyzed TCGA‐LIHC, CHCC‐HBV, LIRI‐JP, and GEO cohorts, TUBA1B expression was significantly elevated in tumor tissues compared with adjacent liver (Figures 1C–E and S2). This upregulation pattern was also observed across six independent GEO datasets, including GSE124535, GSE144269, GSE69164, GSE77314, GSE77509, and GSE94660 (Figure S2D), suggesting that the elevated expression of TUBA1B was not restricted to a single discovery cohort. This finding was further confirmed at the protein level by western blotting and immunohistochemistry in independent clinical samples (Figure 1F,G). Clinically, high TUBA1B expression was associated with advanced tumor stage, poor differentiation, and elevated serum AFP levels (Table S1). Survival analyses demonstrated that patients with high TUBA1B expression exhibited significantly reduced overall, disease‐specific, progression‐free, and disease‐free survival (Figure 1H and S3A). Consistently, analysis of the GSE14520 cohort linked elevated TUBA1B expression to increased metastatic risk (Figure S3B).

### 2.2 HCC Cells Exhibit a Functional Dependency on TUBA1B for Growth and Dissemination

**FIGURE 2:**
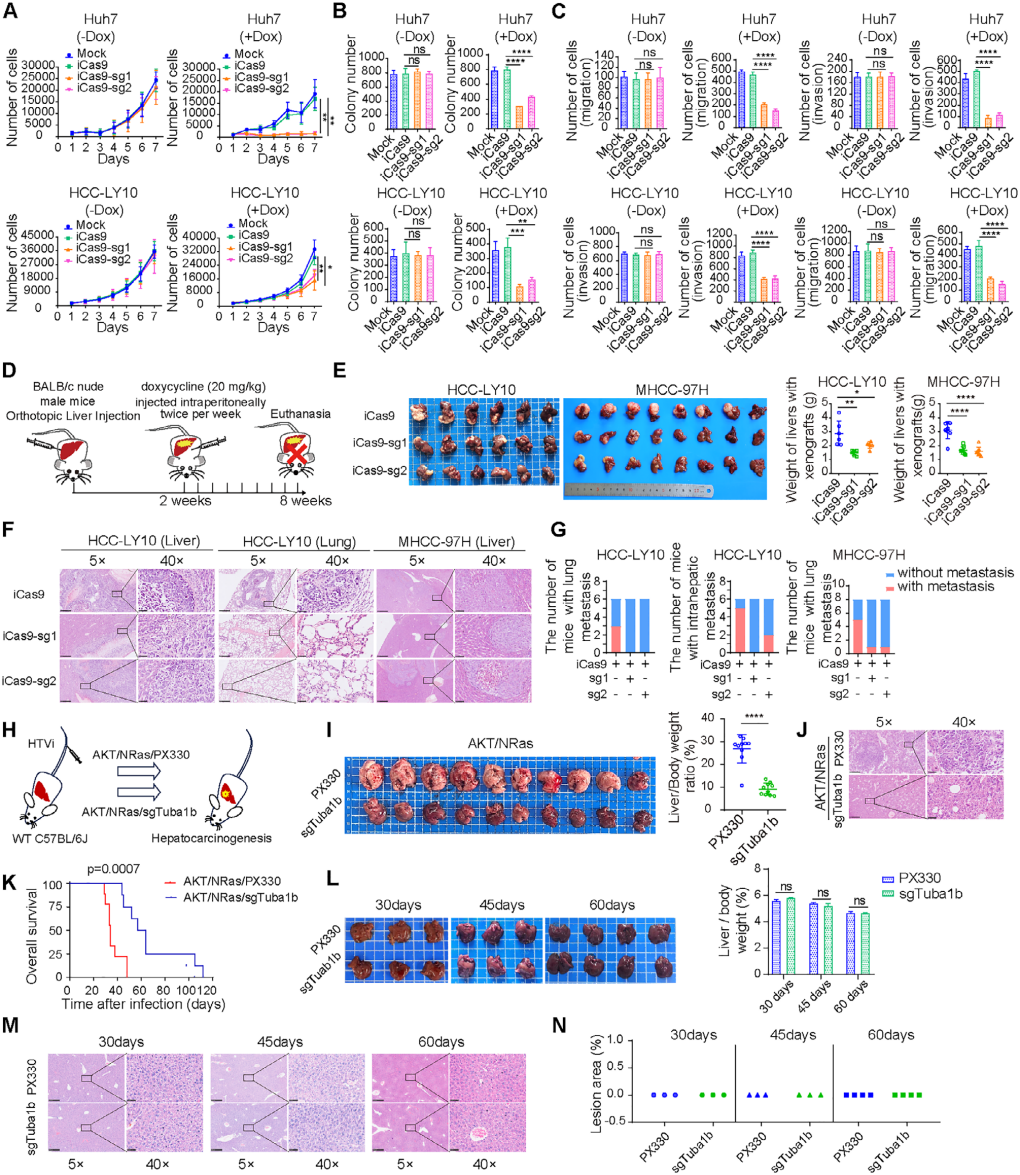
Functional requirement of TUBA1B for HCC cell growth and dissemination. (A, B) Cell growth of control (−Dox) and TUBA1B‐depleted (+Dox) HCC cells assessed by Celigo imaging cytometry (A) and colony formation assays (B). (C) Transwell migration and Matrigel invasion assays assessing migratory and invasive capacities upon doxycycline‐induced TUBA1B depletion. (D) Schematic of the orthotopic HCC xenograft model. (E) Representative liver images from xenograft‐bearing mice with corresponding liver weight quantification (HCC‐LY10, *n* = 6 per group; MHCC‐97H, *n* = 8 per group). (F, G) Representative intrahepatic (HCC‐LY10, MHCC‐97H) and lung metastatic lesions (HCC‐LY10), with quantification and statistical analysis. (H–J) Hydrodynamic tail‐vein injection (HTVi) experiments showing liver images, liver‐to‐body weight ratios, and H&E staining of liver sections from *NRasV12/myr‐AKT + *vector or sg*Tuba1b* groups (*n* = 10 per group). (K) Kaplan–Meier survival analysis of C57BL/6 mice following HTVi of *NRasV12/myr‐AKT* together with control or sg*Tuba1b* plasmids (*n* = 10 per group). (L–N) Gross liver images and liver‐to‐body weight ratios (L), representative H&E staining (M) with lesion area quantification (N) of mice injected with *PX330* or *sgTuba1b* alone at indicated time points (*n* = 10). Scale bar, 100 µm. Data are presented as means ± SD from three independent biological replicates; ns, not significant; *p < 0.05, **p < 0.01, ***p < 0.001, ****p < 0.0001.

To examine the functional contribution of TUBA1B to HCC progression, we silenced TUBA1B in representative HCC cell lines (Figure S3C,D; Figure S4A–F). TUBA1B depletion significantly impaired cell proliferation, colony formation, and DNA synthesis (Figure 2A,B and S5A–G). In addition to reduced growth, loss of TUBA1B markedly suppressed metastatic behaviors, including cell migration, invasion, and adhesion (Figure 2C; Figures S6A–C and S7). Given the canonical role of TUBA1B as a tubulin isotype, we next examined whether TUBA1B depletion affected cytoskeletal organization in HCC cells. Phalloidin staining showed that TUBA1B depletion increased F‐actin stress fiber formation in HCC cells, as reflected by a higher percentage of stress fiber‐positive cells (Figure S6D). These findings indicate that TUBA1B contributes to cytoskeletal homeostasis in HCC cells and suggest that cytoskeletal remodeling may contribute, at least in part, to the impaired migratory and invasive phenotypes caused by TUBA1B loss. Re‐expression of TUBA1B restored proliferative and migratory capacities, confirming the specificity of these effects (Figure S8A–C).

In vivo, doxycycline‐inducible TUBA1B knockdown significantly reduced tumor growth and decreased the incidence of spontaneous intrahepatic and pulmonary metastases in orthotopic xenograft models (Figure 2D–G). To further assess the requirement for TUBA1B in a physiologically relevant setting, we employed a hydrodynamic tail‐vein injection (HTVi) model. Co‐delivery of sg*Tuba1b* markedly attenuated *NRasV12/myr‐AKT–*driven hepatocarcinogenesis, resulting in reduced tumor burden and prolonged survival (Figures 2H–K and S8D–G). Notably, hydrodynamic delivery of sgTuba1b alone did not induce overt hepatic toxicity or morphological abnormalities (Figure 2L–N). To test whether the effect of *Tuba1b* perturbation was restricted to the *NRasV12/myr‐AKT* context, we further examined sg*Tuba1b* in a genetically distinct *MYC/sg‐p53* HCC model. Co‐delivery of sg*Tuba1b* similarly reduced liver tumor burden in this model, supporting a tumor‐promoting role of *Tuba1b* in more than one oncogenic context (Figure S8H,I).

### 2.3 TUBA1B Supports Basal p65 Activity Under Unstimulated Conditions

**FIGURE 3:**
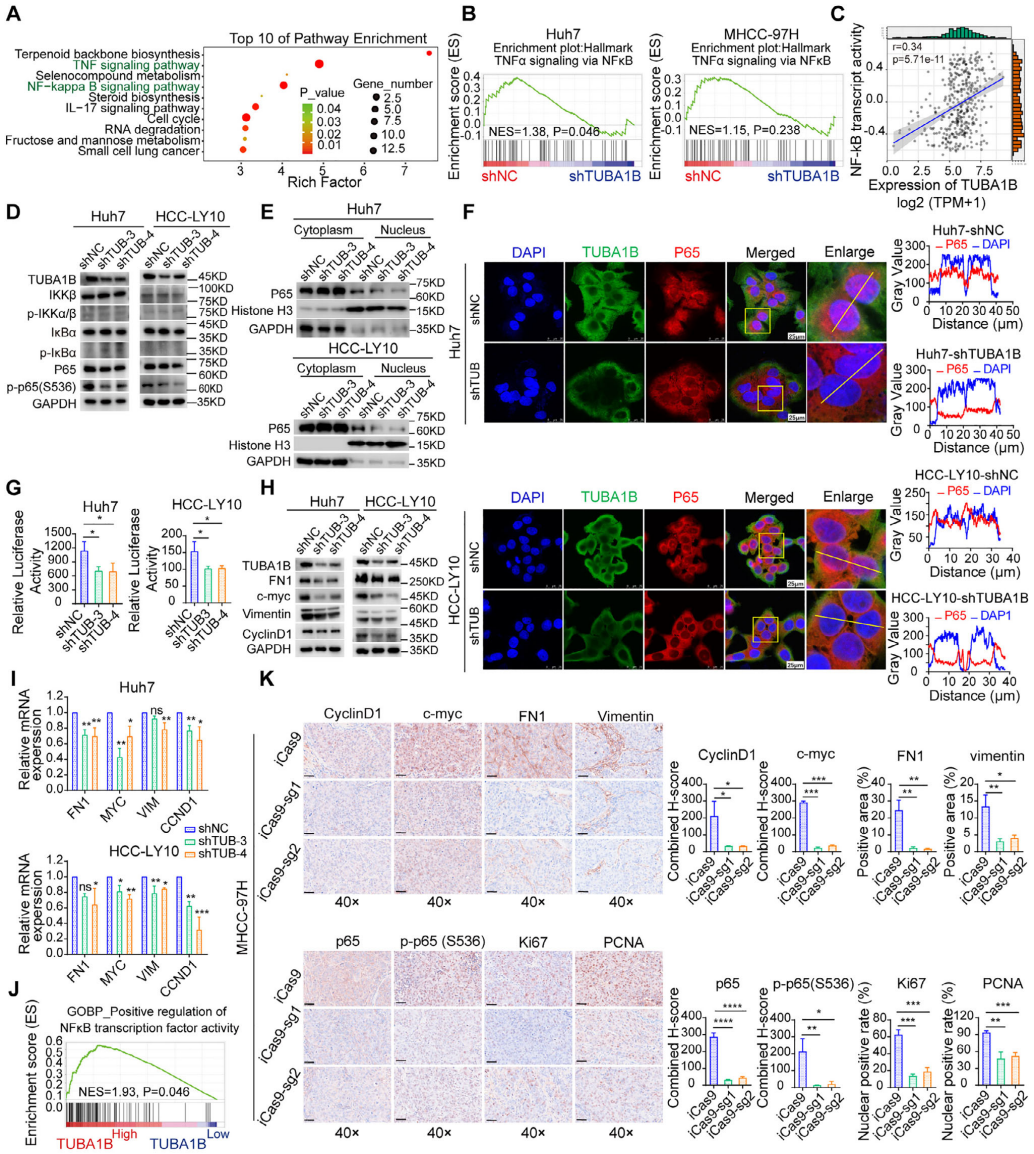
TUBA1B depletion inhibits p65 nuclear translocation and suppresses p65‐dependent NF‐κB transcriptional activity. (A) KEGG pathway enrichment analysis of signaling pathways altered by TUBA1B knockdown in MHCC‐97H cells. (B) GSEA shows TNFα/NF‐κB pathway enrichment in control versus TUBA1B‐depleted cells. (C) Correlation analysis between TUBA1B expression and NF‐κB activity in the TCGA‐LIHC cohort. (D) Western blot analysis of NF‐κB components and phosphorylation following TUBA1B knockdown. (E, F) Nuclear–cytoplasmic fractionation (E) and quantitative immunofluorescence (F) analyses of p65 localization, with GAPDH and Histone H3 marking cytoplasmic and nuclear fractions. (G) NF‐κB luciferase reporter assays assessing transcriptional activity upon TUBA1B depletion. (H, I) Protein (H) and transcript (I) levels of representative NF‐κB target genes following TUBA1B depletion. (J) GSEA of NF‐κB pathway activity in the TCGA‐LIHC cohort. (K) Representative H&E and immunohistochemical staining of xenograft tumors showing NF‐κB target genes (FN1, c‐MYC, VIM, CCND1) and proliferation markers (Ki67, PCNA). Data are presented as means ± SD from three independent biological replicates; *p < 0.05, **p < 0.01, ***p < 0.001, ****p < 0.0001. Original blots can be found in Figure S17‐2–S17‐4.

Transcriptomic analysis of Huh7 and MHCC‐97H cells collectively implicated suppression of TNF/NF‐κB signaling upon TUBA1B knockdown, as supported by KEGG pathway enrichment and GSEA (Figure 3A,B). Consistently, TUBA1B expression positively correlated with NF‐κB pathway signatures in the TCGA‐LIHC cohort (Figures 3C and S9A). At the protein level, TUBA1B knockdown selectively reduced basal phosphorylation of p65 at Ser536 without affecting total IKK, IκBα, or p65 abundance (Figures 3D and S9B). Canonical IKK activation remained undetectable under basal conditions but was readily induced by H_2_O_2_ stimulation (Figure S9C), indicating that TUBA1B supports basal p65 activity independently of acute pathway activation.

Accordingly, TUBA1B knockdown markedly impaired nuclear accumulation of p65 and p65‐dependent transcriptional activity, as demonstrated by subcellular fractionation, immunofluorescence, and reporter assays (Figures 3E–G and S9D,E). Expression of canonical NF‐κB target genes involved in proliferation and epithelial–mesenchymal transition, including c‐MYC, Cyclin D1, FN1, and Vimentin, was concomitantly reduced at both mRNA and protein levels (Figures 3H,I and S9F,G), and positively correlated with TUBA1B expression in TCGA‐LIHC (Figures 3J and S9H). These effects were also evident in xenograft tumors, where TUBA1B depletion decreased p‐p65 (Ser536) levels, nuclear p65 accumulation, and expression of proliferation and EMT markers (Figure 3K). In parallel, GSEA of HCC cells and TCGA‐LIHC cohorts showed enrichment of proliferative, EMT, and metastatic gene programs in tumors with high TUBA1B expression (Figure S9I–N).

### 2.4 TUBA1B Scaffolds G3BP2 to Maintain the G3BP2–IκBα Complex

**FIGURE 4:**
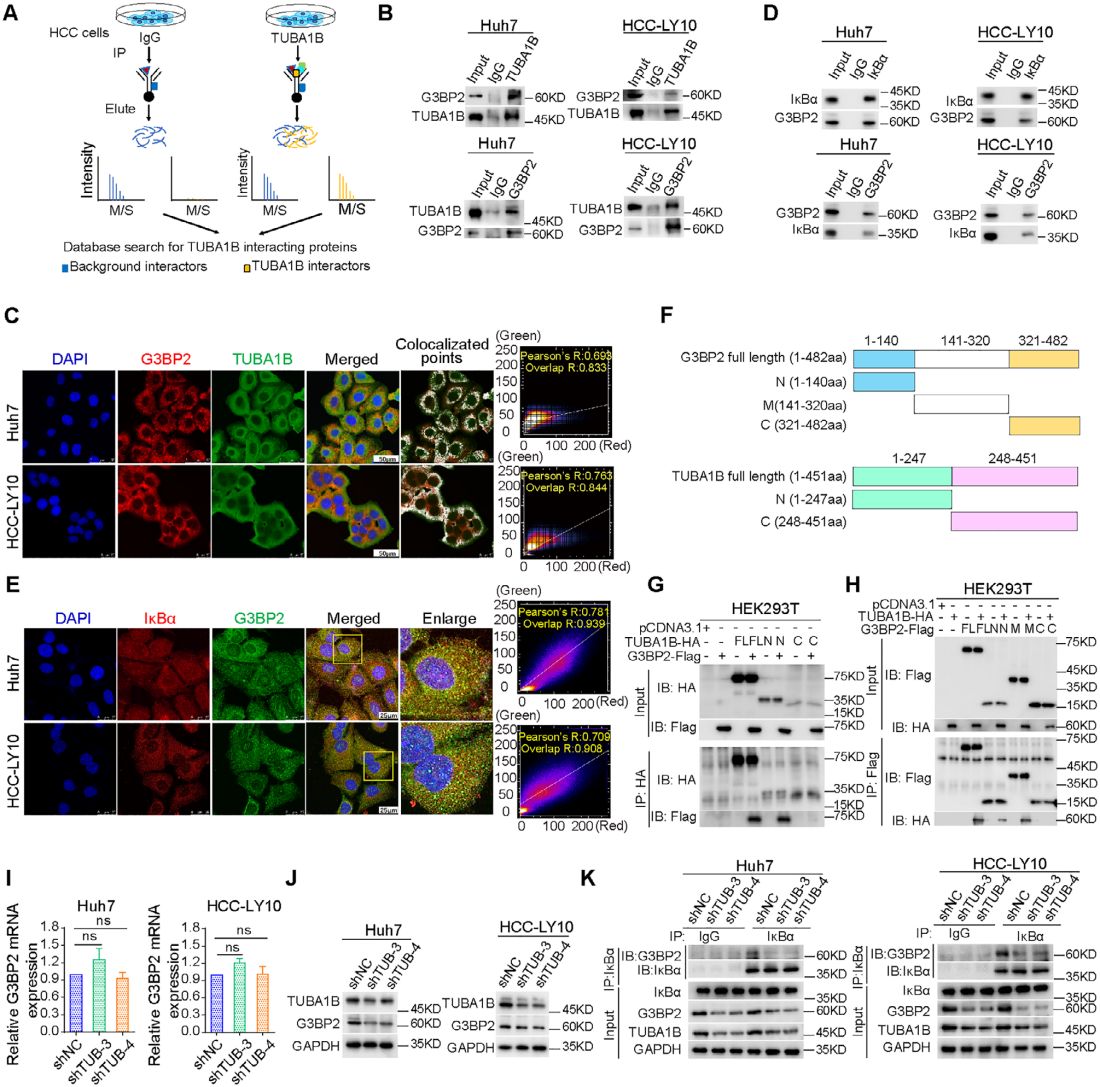
TUBA1B interacts with G3BP2 and supports formation of the G3BP2–IκBα complex. (A) Schematic of TUBA1B interactor identification by immunoprecipitation. (B) Endogenous interaction between TUBA1B and G3BP2 confirmed by co‐immunoprecipitation in Huh7 and HCC‐LY10 cells. (C) Confocal microscopy showing co‐localization of TUBA1B and G3BP2, with Pearson's and Manders’ coefficients. (D) Interaction between G3BP2 and IκBα validated by co‐immunoprecipitation in HCC cells. (E) Co‐localization of G3BP2 and IκBα assessed by confocal microscopy. (F) Schematic of TUBA1B and G3BP2 truncation constructs used for interaction domain mapping. (G) Co‐immunoprecipitation analysis of HA‐tagged TUBA1B truncations with Flag‐tagged G3BP2‐FL in HEK293T cells. (H) Co‐immunoprecipitation analysis of Flag‐tagged G3BP2 truncations with HA‐tagged TUBA1B‐FL. (I, J) G3BP2 mRNA (I) and protein (J) levels following TUBA1B knockdown. (K) Reduced G3BP2–IκBα complex formation upon TUBA1B depletion. Original blots can be found in Figure S17‐5–S17‐10.

To further explore how TUBA1B sustains p65/NF‐κB activity in HCC cells, we performed TUBA1B co‐immunoprecipitation followed by mass spectrometry in Huh7 and MHCC‐97H cells. This analysis identified 33 overlapping TUBA1B‐associated protein entries/protein groups shared by the two HCC cell lines (Figures 4A and S10A,B). We next examined whether any of these shared candidates could provide a plausible mechanistic link between TUBA1B‐associated assemblies and p65/NF‐κB regulation. Among these candidates, G3BP2 was prioritized for further validation because it was reproducibly enriched in both HCC cell lines and has reported links to stress granule‐associated signaling complex organization, IκBα/NF‐κB regulation, and cancer‐associated phenotypes [21, 22, 23, 24, 25]. The interaction between TUBA1B and G3BP2 was supported by molecular docking analysis (Figure S10C) and further validated by reciprocal Co‐IP and immunofluorescence co‐localization assays (Figures 4B,C and S10D,E). Consistent with the established role of G3BP2 in SG assembly and signaling complex organization [22, 23, 24], Co‐IP analyses confirmed an interaction between G3BP2 and IκBα (Figures 4D and S10F). Immunofluorescence analyses further revealed that IκBα signals were frequently detected in close association with G3BP2‐positive puncta in HCC cells (Figures 4E and S10G), consistent with spatial colocalization of IκBα within G3BP2‐enriched cytoplasmic structures.

To further explore the molecular basis underlying this cytoskeleton–stress granule coupling, truncation mutants of TUBA1B (full length, N‐terminal, and C‐terminal fragments) and G3BP2 (full length, N‐terminal, middle, and C‐terminal fragments) were co‐expressed in HEK293T cells. TUBA1B full‐length and N‐terminal fragments bound to G3BP2, whereas the middle and C‐terminal regions of G3BP2 retained interaction with TUBA1B (Figure 4G,H), identifying regions involved in the TUBA1B–G3BP2 association. Notably, depletion of TUBA1B markedly reduced G3BP2 protein abundance without affecting its mRNA expression (Figures 4I,J and S10H,I), indicating post‐transcriptional regulation of G3BP2 stability. Consistent with this observation, loss of TUBA1B significantly impaired the formation of the G3BP2–IκBα complex (Figures 4K and S10J), suggesting that TUBA1B is required to maintain G3BP2‐associated p65 regulatory assemblies. These observations raise the question of how G3BP2‐containing assemblies are maintained at sufficient levels to support sustained NF‐κB regulation in HCC cells.

### 2.5 TUBA1B Preserves G3BP2 Stability by Restraining TRIM25‐Dependent Ubiquitination

**FIGURE 5:**
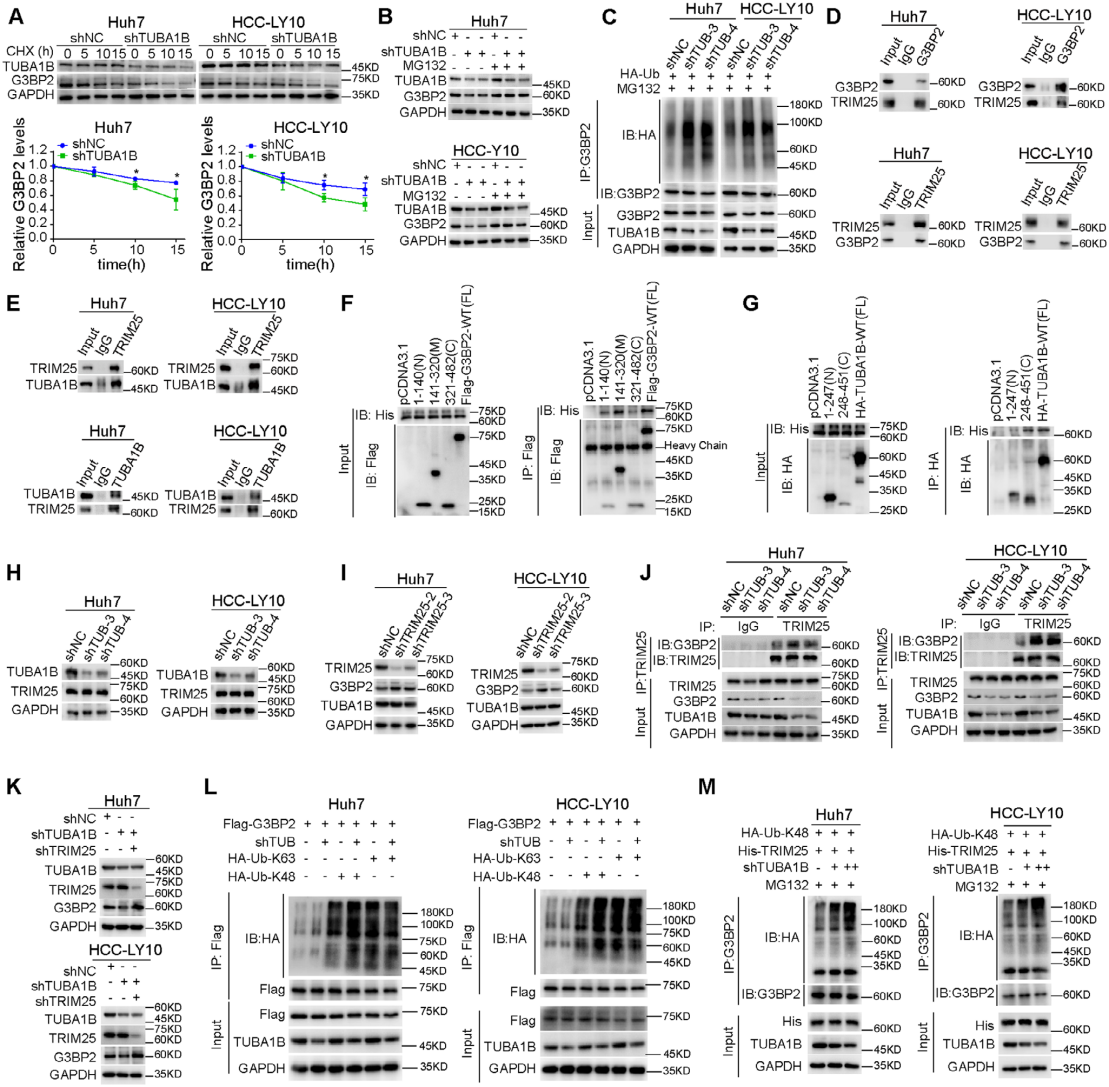
TUBA1B regulates G3BP2 stability in a TRIM25‐dependent manner. (A) Cycloheximide chase assays showing reduced G3BP2 stability following TUBA1B depletion. (B) Immunoblot analysis of G3BP2 protein levels after TUBA1B knockdown with or without MG132 treatment. (C) G3BP2 ubiquitination assay under MG132‐treated conditions. All indicated groups were transfected with HA‐tagged ubiquitin and treated with MG132 before harvest. G3BP2 was immunoprecipitated, and ubiquitinated G3BP2 was detected by immunoblotting with an anti‐HA antibody. (D) Endogenous interaction between G3BP2 and TRIM25 confirmed by co‐immunoprecipitation in HCC cells. (E) Endogenous interaction between TUBA1B and TRIM25 detected by co‐immunoprecipitation. (F, G) Mapping of interaction domains between TRIM25 and truncated G3BP2 or TUBA1B constructs. (H) TRIM25 protein expression following TUBA1B depletion. (I) G3BP2 and TUBA1B protein expression following TRIM25 knockdown. (J) Co‐immunoprecipitation analysis of the G3BP2–TRIM25 interaction in control and TUBA1B‐depleted cells. (K) G3BP2 protein levels in TUBA1B‐depleted cells with or without TRIM25 knockdown. (L) K48‐ and K63‐linked ubiquitination assays of G3BP2 in HCC cells. Ubiquitinated G3BP2 was detected as a broad HA‐ubiquitin smear by immunoblotting with an anti‐HA antibody and interpreted based on the overall smear pattern within each cell‐line panel. (M) Co‐immunoprecipitation analysis showing that progressive TUBA1B depletion dose‐dependently increases TRIM25‐mediated K48‐linked ubiquitination of G3BP2. His‐TRIM25 was transfected at a fixed amount, whereas shTUBA1B plasmids were transfected in increasing amounts. Data are presented as means ± SD from three independent biological replicates; *p < 0.05, **p < 0.01, ***p < 0.001, ****p < 0.0001. Original blots can be found in Figure S17‐11–S17‐23.

To determine how TUBA1B maintains G3BP2 abundance, we examined G3BP2 protein stability. Cycloheximide (CHX) chase assays showed that TUBA1B knockdown significantly shortened the half‐life of G3BP2 in HCC cells, and this effect was reversed by the proteasome inhibitor MG132, indicating proteasome‐dependent degradation (Figures 5A,B and S11A,B). Consistently, TUBA1B depletion increased G3BP2 ubiquitination (Figures 5C and S11C). TRIM25 has been reported to mediate both K48 and K63‐linked ubiquitination of G3BP2 during viral infection, with functional implications in carcinogenesis and antiviral responses [26, 27, 28]. We therefore assessed whether TRIM25 contributes to G3BP2 turnover in HCC cells. Co‐immunoprecipitation confirmed an interaction between endogenous G3BP2 and TRIM25 (Figures 5D and S11D). An endogenous interaction between TUBA1B and TRIM25 was also detected by co‐immunoprecipitation (Figure 5E and S11H). TRIM25 knockdown increased G3BP2 protein levels (Figures 5I and S11E), whereas TRIM25 overexpression reduced G3BP2 abundance in a dose‐dependent manner (Figure S11F). A TRIM25 ΔRING mutant failed to decrease G3BP2 levels, indicating that TRIM25 E3 ligase activity is required for G3BP2 turnover (Figure S11G).

To further define the structural relationships underlying this regulatory axis, we analyzed the interaction domains among TUBA1B, G3BP2, and TRIM25 using truncation mutants. TRIM25 showed the strongest association with the middle region of G3BP2 (Figure 5F), whereas the C‐terminal region of TUBA1B retained interaction with TRIM25 (Figure 5G). Together with our earlier mapping showing that the middle region of G3BP2 interacts with TUBA1B (Figure 4H), these data indicate that the middle region of G3BP2 is involved in binding to both TUBA1B and TRIM25. Importantly, TUBA1B depletion did not alter TRIM25 protein abundance, nor did TRIM25 knockdown affect TUBA1B expression (Figures 5H,I and S11E,I), indicating that TUBA1B does not regulate TRIM25 at the expression level. Functionally, depletion of TUBA1B enhanced the interaction between G3BP2 and TRIM25, whereas TRIM25 knockdown restored G3BP2 protein levels in TUBA1B‐deficient cells (Figures 5J,K and S11J,K). These findings suggest that endogenous TUBA1B limits productive TRIM25 engagement with G3BP2. Moreover, loss of TUBA1B selectively increased K48‐linked, but not K63‐linked, ubiquitination of G3BP2 (Figures 5L and S11L). Conversely, TUBA1B inhibited TRIM25‐mediated K48‐linked ubiquitination of G3BP2 in a dose‐dependent manner (Figure 5M). Collectively, these findings indicate that TUBA1B preserves G3BP2 stability by restricting TRIM25‐associated K48‐linked ubiquitination of G3BP2, thereby sustaining the availability of G3BP2 for cytoskeleton‐associated regulation of NF‐κB signaling.

### 2.6 G3BP2 Mediates TUBA1B‐Dependent p65 Activation and Malignant Phenotypes

**FIGURE 6:**
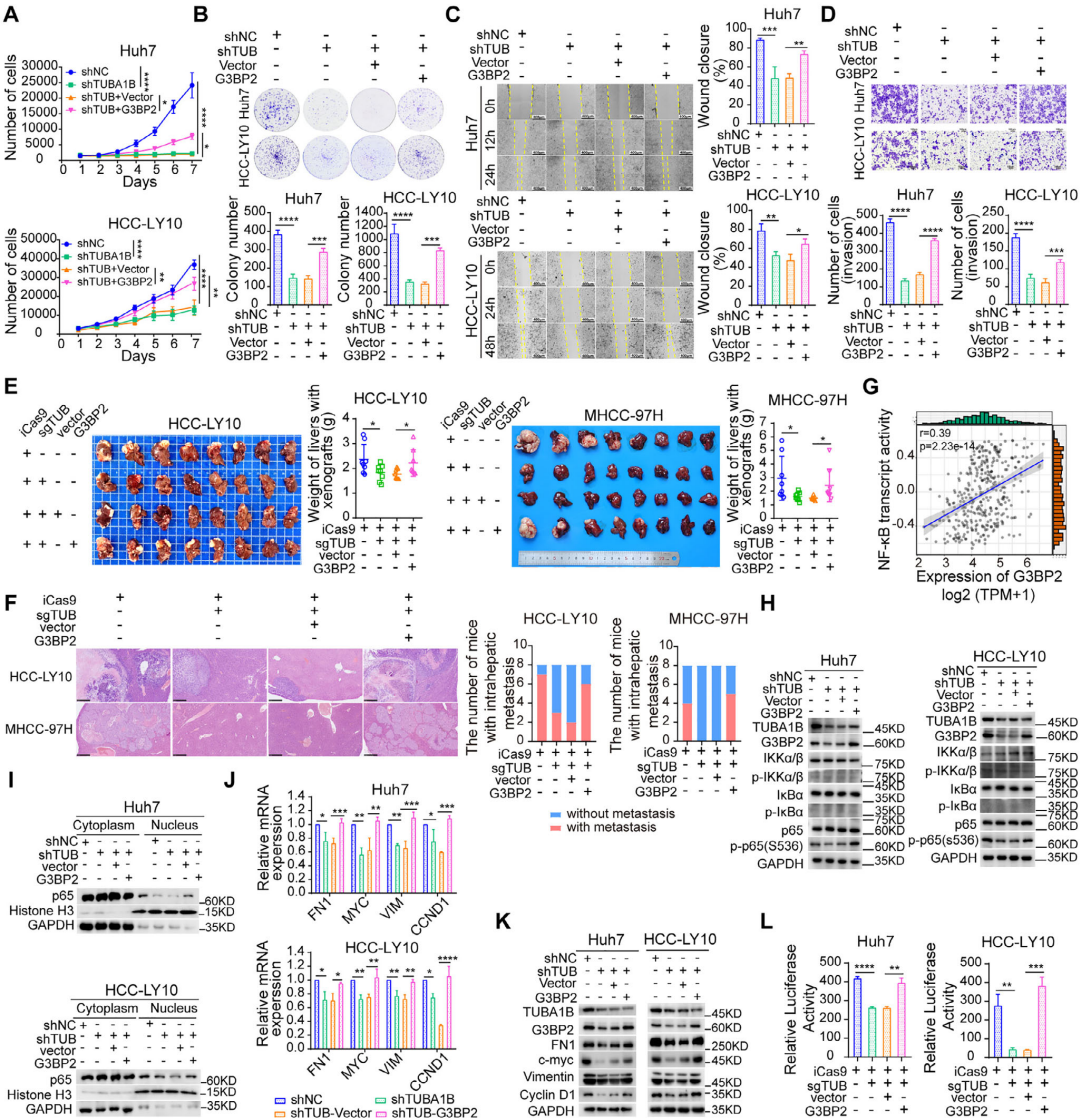
G3BP2 re‐expression rescues p65 activity and associated phenotypes in TUBA1B‐deficient HCC models. (A–D) Celigo proliferation, colony formation, wound‐healing migration, and Matrigel invasion assays in HCC cells with G3BP2 overexpression in the setting of TUBA1B knockdown. (E, F) Orthotopic xenograft models (HCC‐LY10 and MHCC‐97H) showing that G3BP2 overexpression restores tumor growth and metastasis suppressed by TUBA1B knockdown; liver weight, metastatic foci, and representative intrahepatic lesions are shown. (G) TCGA‐LIHC analysis showing correlation between G3BP2 expression and NF‐κB activity. (H, I) Immunoblotting and subcellular localization assays showing restoration of p65 phosphorylation and nuclear accumulation upon G3BP2 overexpression in TUBA1B‐knockdown cells. (J, K) NF‐κB target gene expression at the mRNA (J) and protein (K) levels. (L) NF‐κB luciferase reporter activity. Data are presented as means ± SD from three independent biological replicates; *p < 0.05, **p < 0.01, ***p < 0.001, ****p < 0.0001. Original blots can be found in Figure S17‐24–S17‐26.

Given the role of TUBA1B in stabilizing G3BP2 and organizing G3BP2‐containing assemblies, we asked whether G3BP2 is required for TUBA1B‐dependent maintenance of basal p65 signaling output in HCC cells. Silencing of G3BP2 markedly suppressed cell proliferation, migration, and invasion, whereas ectopic expression of G3BP2 enhanced these malignant phenotypes (Figure S12A–H). Importantly, re‐expression of G3BP2 in TUBA1B‐deficient cells largely rescued impaired proliferative, migratory, and invasive capacities both in vitro and in vivo (Figures 6A–F and S13A–D), indicating that G3BP2 acts downstream of TUBA1B to sustain malignant behaviors. Consistent with this functional dependency, analysis of the TCGA‐LIHC cohort revealed a positive correlation between G3BP2 expression and NF‐κB pathway activity (R = 0.39, *p* < 0.0001), as well as with canonical NF‐κB target genes involved in proliferation and epithelial–mesenchymal transition, including FN1, c‐MYC, Vimentin, Cyclin D1, Snail1, and Twist1 (Figures 6G and S13E). Restoration of G3BP2 in TUBA1B‐knockdown cells was sufficient to recover basal phosphorylation of p65 at Ser536, promote nuclear accumulation of p65, and restore p65 target gene expression at both the mRNA and protein levels (Figures 6H–K and S13F–I). Consistently, immunofluorescence staining further showed that TUBA1B depletion reduced p65 nuclear localization, whereas G3BP2 re‐expression restored p65 nuclear accumulation in TUBA1B‐deficient HCC cells (Figure S13K). Dual‐luciferase reporter assays further confirmed that G3BP2 re‐expression reinstated NF‐κB transcriptional activity under basal conditions (Figures 6L and S13J). Together, these findings establish G3BP2 as a key downstream effector through which TUBA1B sustains constitutive NF‐κB signaling and malignant phenotypes in hepatocellular carcinoma. To further assess the relevance of TUBA1B to inflammatory signaling and the immune context of HCC, we examined cytokine‐responsive transcription, immune‐related features, and immune‐cell composition. TUBA1B depletion attenuated the induction of CCL2 following TNFα or IL‐6 stimulation (Figure S15A,B). In the TCGA‐LIHC cohort, TUBA1B expression was associated with multiple immune‐ and stroma‐related features, including immune exclusion, IFNG, CD274/PD‐L1, MDSC, and CAF scores, while stratification by TUBA1B expression revealed significant differences in dysfunction, exclusion, CAF, and TIDE scores (Figure S15C,D). In the *AKT/NRAS*‐driven HCC model, *Tuba1b* perturbation was accompanied by increased hepatic NK1.1‐positive cells and decreased B cells, as well as increased splenic CD45‐positive and NK1.1‐positive cells and decreased neutrophils (Figure S15E–H).

### 2.7 Concurrent Upregulation of TUBA1B and G3BP2 Defines a High‐Risk HCC Subgroup With Sustained p65 Activation

**FIGURE 7:**
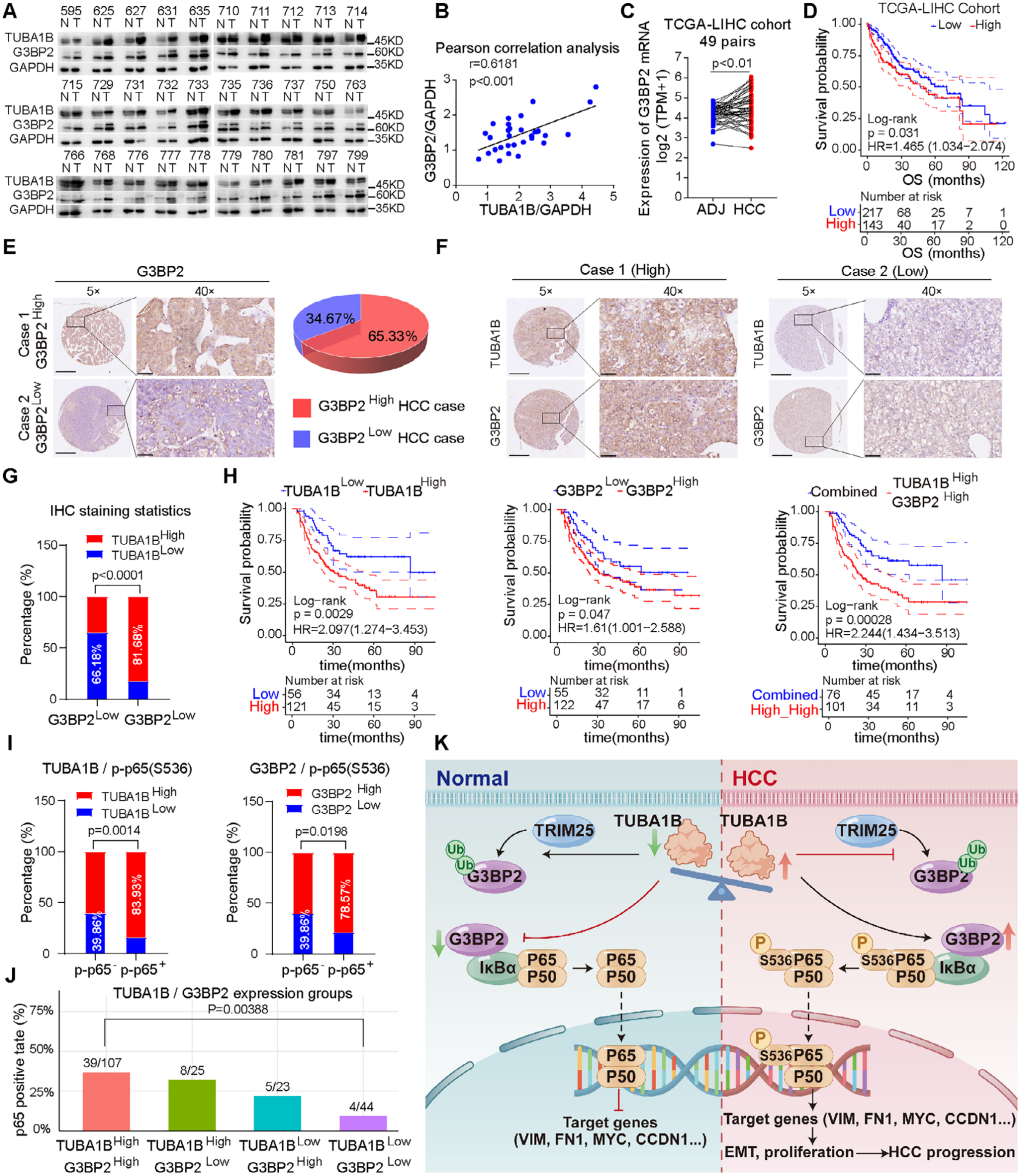
Co‐upregulation of TUBA1B and G3BP2 associates with p65 activation and adverse outcome in HCC. (A) Immunoblot of TUBA1B and G3BP2 in 30 paired HCC and adjacent tissues. (B) Pearson correlation between TUBA1B and G3BP2 protein levels in HCC tissues. (C) G3BP2 expression in TCGA‐LIHC tumors versus adjacent liver. (D) Kaplan–Meier analysis of overall survival stratified by G3BP2 expression (TCGA‐LIHC). (E) Representative IHC images and scoring distribution of G3BP2 in HCC specimens. (F, G) Representative IHC staining for TUBA1B and G3BP2 and their tissue‐level correlation (*n* = 199). (H) Kaplan–Meier survival analysis of patients stratified by joint TUBA1B and G3BP2 expression status. (I) Association of TUBA1B or G3BP2 expression with nuclear p‐p65 (Ser536) positivity (χ^2^ test). (J) Distribution of nuclear p‐p65 (Ser536) positivity across TUBA1B/G3BP2 expression groups. (K) Working model of TUBA1B–G3BP2–NF‐κB regulation in HCC. Original blots can be found in Figure S17‐27.

To assess the clinical relevance of the TUBA1B–G3BP2 axis in HCC, we analyzed tumor specimens from multiple independent cohorts. G3BP2 protein levels were markedly elevated in HCC tissues and positively correlated with TUBA1B expression, as confirmed by western blotting and immunohistochemistry in paired clinical samples (Figure 7A–G). Consistently, analyses of TCGA‐LIHC and additional public cohorts revealed significantly higher G3BP2 expression in tumors compared with adjacent liver tissues (Figures 7C and S14A,B). Kaplan–Meier analysis demonstrated that high G3BP2 expression was associated with reduced overall survival in the TCGA‐LIHC cohort (Figure 7D). Notably, patients with concurrent high expression of TUBA1B and G3BP2 exhibited the poorest prognosis compared with all other expression groups, whereas high expression of either factor alone conferred an intermediate risk (Figures 7H and S14C).

Consistent with these clinical outcomes, high expression of TUBA1B and/or G3BP2 was significantly associated with nuclear p‐p65 (Ser536) positivity in HCC specimens, with the TUBA1B/G3BP2 double‐high group showing the highest frequency of NF‐κB activation (Figures 7I,J and S14D,E). Multivariable analysis showed that TUBA1B expression exhibited a stronger association with nuclear p‐p65 positivity than G3BP2 (Figure S14F), suggesting a model in which TUBA1B acts upstream of G3BP2 in organizing sustained p65 activation.

## 3 Discussion

In this study, we show that the α‐tubulin isoform TUBA1B contributes to the maintenance of constitutive p65/NF‐κB transcriptional state in hepatocellular carcinoma, extending its function beyond a purely structural role in microtubules. Consistent with its canonical cytoskeleton‐related function, TUBA1B depletion induced F‐actin cytoskeletal remodeling, which may partly contribute to the impaired migratory and invasive phenotypes observed upon TUBA1B loss. Beyond its structural role in cytoskeletal homeostasis, our study further links TUBA1B to the maintenance of basal p65/NF‐κB signaling through regulation of G3BP2 protein stability. Consistent with this model, TUBA1B expression is elevated in HCC and correlates with poor clinical outcomes, including metastasis risk. Functional perturbation experiments revealed that HTVi‐mediated co‐expression of *Tuba1b* enhances *myr‐AKT–*driven liver tumor burden, whereas *Tuba1b* perturbation suppresses tumor progression across multiple experimental models, including both *NRasV12/myr‐AKT*‐driven and *MYC/sg‐p53* HCC contexts. Together, these observations identify TUBA1B as a previously unappreciated determinant of malignant progression in HCC.

Mechanistically, this p65‐sustaining effect is mediated by the TUBA1B–G3BP2 stability axis. We find that TUBA1B limits TRIM25 binding to G3BP2 and thereby restrains TRIM25‐associated K48‐linked ubiquitination of G3BP2. Our domain‐mapping assays showed that the middle region of G3BP2 is involved in binding to both TUBA1B and TRIM25, while the C‐terminal region of TUBA1B contributes to its interaction with TRIM25. TUBA1B depletion further enhanced G3BP2–TRIM25 association. These findings support a model in which TUBA1B reduces TRIM25 access to G3BP2, although the precise structural basis of this regulation remains to be defined. Previous studies have shown that TRIM25 can promote protein degradation in cancer contexts [26, 27, 28]. By limiting TRIM25‐mediated degradative ubiquitination of G3BP2, TUBA1B preserves G3BP2 protein stability. Both TUBA1B and G3BP2 have been individually implicated in tumor proliferation and metastasis [17, 25], yet how these factors converge at the level of inflammatory signaling has remained unclear. G3BP2 functions as a core stress granule component capable of assembling multivalent protein complexes [21, 22, 23, 24], and has been shown to modulate the subcellular behavior of IκBα under inflammatory conditions [25, 29]. In line with these reports, our data suggest that TUBA1B‐dependent stabilization of G3BP2 may influence the G3BP2–IκBα regulatory module, thereby contributing to sustained basal p65 activity.

This regulatory mode is distinct from canonical NF‐κB activation, which relies on IKK‐dependent phosphorylation and proteasomal degradation of IκBα [30]. Instead, our data support a model in which the TUBA1B–G3BP2 module modulates the functional availability of IκBα, thereby sustaining p65 transcriptional output under basal conditions without overt canonical IKK activation. In addition to controlling NF‐κB nuclear localization, the TUBA1B–G3BP2 axis also maintains basal phosphorylation of p65 at Ser536, a modification known to enhance NF‐κB transcriptional output [31, 32]. Phosphorylation of IKK and IκBα was undetectable under basal conditions, consistent with their low constitutive activity [33, 34]. Together with the G3BP2 rescue experiments, these findings indicate that G3BP2 is functionally involved in sustaining basal p65 Ser536 phosphorylation, although the responsible upstream kinase mechanism remains to be defined. Prior studies suggest that kinases such as CK2, MSK1, and IKKε may be involved in regulating this modification [25, 35, 36]. Thus, the TUBA1B–G3BP2 axis appears to support basal NF‐κB transcriptional activity through both IκBα‐related regulation of p65 localization and maintenance of p65 Ser536 phosphorylation. Rather than acting as a driver of acute canonical NF‐κB activation, this axis may help maintain a permissive NF‐κB signaling state that supports malignant progression in HCC. Given that HCC commonly develops in chronically inflamed livers, the TUBA1B–G3BP2 axis may help tumor cells maintain p65/NF‐κB activity and selected inflammatory transcriptional responses within a cytokine‐rich microenvironment. Consistent with this possibility, TUBA1B depletion attenuated cytokine‐induced CCL2/CXCL8 expression, while TUBA1B expression was associated with immune‐related features and Tuba1b perturbation was accompanied by changes in selected immune‐cell populations. These findings suggest that this axis may facilitate tumor‐cell adaptation to an established inflammatory environment, although they do not demonstrate that TUBA1B directly initiates hepatic inflammation or remodels the immune microenvironment.

The clinical relevance of this axis is highlighted by the observation that concurrent high TUBA1B and G3BP2 expression identifies HCC patients with the strongest p65 activity and worst outcomes. Importantly, HTVi‐mediated co‐expression of *Tuba1b* with *myr‐AKT* increased liver tumor burden, further supporting its isoform‐specific tumor‐promoting activity in vivo, whereas TUBA1B depletion impaired tumor progression without overt hepatocyte toxicity in the tested models. However, given the high conservation of tubulin isoforms and the essential physiological roles of microtubules, direct pharmacological targeting of TUBA1B may face challenges in isoform selectivity and therapeutic tolerance. Thus, TUBA1B may be better viewed as a functional dependency marker reflecting reliance on the G3BP2–p65/NF‐κB signaling state, rather than as an immediately druggable therapeutic target. Future translational efforts may therefore focus on stratifying tumors according to TUBA1B/G3BP2‐associated p65 activity or on more selective perturbation of the TUBA1B–G3BP2 regulatory axis [37, 38].

Several limitations of this study should be acknowledged. First, the molecular details of the TUBA1B–G3BP2–TRIM25 axis remain incompletely defined. Our findings support a model in which TUBA1B stabilizes G3BP2 and limits TRIM25‐associated K48‐linked ubiquitination, but the relative contribution of competitive binding, conformational changes, altered localization, or complex remodeling remains unclear. How stabilized G3BP2 further modulates IκBα availability and basal p65 activity also warrants further investigation. Second, phalloidin staining showed that TUBA1B depletion increases F‐actin stress fiber formation, supporting a cytoskeleton‐related contribution to the impaired migratory and invasive phenotypes. However, the biomechanical consequences of this remodeling were not directly examined, and this cytoskeletal phenotype alone is insufficient to explain the accompanying changes in G3BP2 stability, TRIM25‐associated K48‐linked ubiquitination, or basal p65/NF‐κB activity. Third, we were unable to include a robust and physiologically representative normal hepatocyte model for direct in vitro comparison, as commonly used immortalized hepatocyte cell lines have limitations in identity and biological suitability, while primary human hepatocytes rapidly lose differentiated features under conventional culture conditions [39, 40, 41]. We therefore used adjacent non‐tumorous liver tissues as the non‐tumorous expression reference. Fourth, although TUBA1B upregulation was reproducibly observed across independent HCC cohorts and *Tuba1b* perturbation suppressed tumor burden in both *NRasV12/myr‐AKT‐*driven and *MYC/sg‐p53* HCC models, these data do not establish TUBA1B as a uniform dependency across all HCC subtypes. Because the cross‐species GSE148379 analysis and the two HTVi models represent selected oncogenic contexts, our data do not fully cover the molecular and etiological heterogeneity of HCC. Therefore, the subtype‐ and etiology‐specific relevance of TUBA1B, including its potential involvement in chronic liver injury‐associated inflammatory or stromal remodeling, will require further validation in additional molecularly defined models and clinically annotated cohorts. In addition, because our current oncogene‐driven models do not recapitulate chronic liver injury or fibrosis, whether the TUBA1B–G3BP2 axis directly contributes to inflammation‐associated hepatocarcinogenesis or immune remodeling remains to be determined. Finally, the feasibility of selectively targeting TUBA1B itself remains uncertain, and future studies should determine whether the TUBA1B–G3BP2 interface or downstream NF‐κB‐dependent vulnerabilities can be exploited with greater therapeutic selectivity.

In summary, we identify an isoform‐specific function of TUBA1B in maintaining constitutive p65 transcriptional output in HCC by stabilizing G3BP2 and modulating the G3BP2–IκBα regulatory module. Co‐upregulation of TUBA1B and G3BP2, together with elevated p65 activity, delineates a high‐risk subgroup with the poorest outcomes, supporting the TUBA1B–G3BP2 axis as a clinically relevant regulatory vulnerability in HCC.

## 4 Experimental Section

Method details and statistical methods are provided in Supplementary Information.

### 4.1 Cell Lines and Cell Culture

The human HCC cell line Huh7 was obtained from the Riken Cell Bank (Tokyo, Japan). The human HCC cell lines MHCC‐LM3 and MHCC‐97H were kindly donated by the Liver Cancer Institute of Zhongshan Hospital at Fudan University (Shanghai, China). Human primary HCC‐LY10 (Patent No. ZL201110069783.5) cell line established in our laboratory was cultured in Williams’ Medium E supplemented with 10% FBS. PLC/PRF/5, Hep3B, HEK‐293T, and Hepa1‐6 cell lines were purchased from the American Type Culture Collection (Manassas, VA, USA). The cells were cultured in DMEM medium containing 10% fetal bovine serum at 37°C in an incubator with a humidified atmosphere of 20% O_2_ and 5% CO_2_. These cell lines were Mycoplasma‐free and routinely authenticated by quality examinations of morphology and the growth profile.

### 4.2 Generation of Inducible TUBA1B KD Cell Lines

The procedure for inducible knockdown of TUBA1B is as follows: First, lentiviral transduction was used to establish doxycycline‐inducible Cas9‐expressing stable cell lines in HCC cells. After 3 days of puromycin selection (2 µg/mL), single clones were isolated via limited dilution cloning. The selected monoclonal line exhibiting the highest Cas9 expression upon 1 µg/mL doxycycline induction was subsequently transduced with sgRNA‐targeting‐TUBA1B (sgTUBA1B‐1, sgTUBA1B‐2) lentivirus to generate inducible TUBA1B‐knockdown cells [42]. Inducible‐Cas9‐sgTUBA1B single clones were also isolated via limited dilution cloning. Due to unsuccessful isolation of monoclonal Cas9‐stable Huh7 cells, pooled Huh7 cells with stable Cas9 expression (Cas9‐stable bulk cells) were instead transduced with sgTUBA1B lentivirus to construct inducible TUBA1B‐knockdown Huh7 cells.

### 4.3 Animal Models

All animal care and experimental procedures were conducted in accordance with the National Institutes of Health Guidelines for the Care and Use of Laboratory Animals and approved by the Ethics Committee of Fudan University Shanghai Cancer Center (Approval No. FUSCC‐IACUC‐2023635). Seven‐week‐old male BALB/C nude mice (obtained from Jiangsu GemPharmatech LLC Co., Ltd) were used for the intrahepatic tumor model. 1 × 10^6^ cells suspended in 40 µL of a 1:1 (v/v) mixture of serum‐free DMEM and Matrigel (BD Biosciences) were injected into the left liver lobe. Two weeks after intrahepatic injection of HCC cells, mice were treated intraperitoneally with doxycycline (20 mg/kg body weight, twice weekly) for 6–8 weeks [43]. Approximately eight weeks after tumor cell injection, mice were sacrificed, and tumor sizes were determined. 6–8‐week‐old male C57BL/6J mice (Shanghai Bikai Keyi Biotechnology Co., Ltd.) were used. For overexpression experiments, *pT3‐myr‐AKT* plasmid (5 µg) was co‐injected with *pT3‐EF1α‐Tuba1b*, *pT3‐EF1α‐Tubb2b*, *pT3‐EF1α‐Tubb*, or *pT3‐EF1α* empty vector (12.5 µg each), together with *SB* transposase plasmid (5 µg). Plasmids were dissolved in 2 mL of sterile saline and rapidly injected (∼5–7 s) via the tail vein [44]. Mice were maintained for 2 months before tissue collection. For knockout experiments in *NRasV12/myr‐AKT–*driven HCC, plasmids encoding *pT3‐myr‐AKT* (7.5 µg), *pT3‐NRasV12* (7.5 µg), and *pT3‐PX330* (10 µg) or *PX330‐sgTuba1b* (10 µg), plus *SB* transposase plasmid (1/25 of total plasmid mass), were similarly injected. For the *MYC/sg‐p53* HCC model, *SB* transposase, *MYC*, *sg‐p53*, and *PX330‐sgTuba1b* plasmids were co‐injected at doses of 15, 25, 25, and 25 µg, respectively; the control group received *SB* transposase, *MYC*, *sg‐p53*, and *PX330* plasmids at the same doses. Mice were sacrificed 19 days after injection. Tumor tissues were analyzed by H&E staining and subjected to histological observation.

### 4.4 H&E Staining and Histological Assessment

Liver and lung tissues were collected at endpoint, fixed in 10% neutral‐buffered formalin, paraffin‐embedded, sectioned (4–5 µm), and stained with hematoxylin and eosin using standard protocols. Slides were examined by blinded investigators. Intrahepatic metastases were defined as discrete tumor nodules separated from the primary lesion by ≥2 mm of normal parenchyma, consistent with the definition of satellitosis as “a nodule separated from the main tumor by a distance greater than that of the satellite diameter” [45, 46]. Tumor thrombi within portal or hepatic veins were classified as microvascular invasion, following the pathological definition where cancer cell nests are present within vascular channels lined by endothelial cells [47]. Lesions along the injection tract without a capsule were excluded as artifacts. Pulmonary metastases were identified as isolated tumor nodules within lung parenchyma.

## Author Contributions

F.L.: Conceptualization, Methodology, Validation, Formal analysis, Investigation, Data curation, Visualization, Writing – original draft. H.C.L.: Conceptualization, Methodology, Validation, Investigation. J.M.Y.: Software, Methodology, Formal analysis, Data curation, Visualization, Writing – review & editing. H.L.: Conceptualization, Methodology, Validation, Formal analysis, Visualization, Funding acquisition, Supervision, Writing – review & editing. J.J.L.: Conceptualization, Resources, Funding acquisition, Supervision, Writing – review & editing. C.G.: Investigation. L.M.: Investigation. Y.Y.W.: Investigation. Y.Z.J.: Investigation. S.H.Z.: Investigation. T.Y.C.: Resources.

## Fundings

This work was supported by the National Natural Science Foundation of China (82173331, 82172904, and 82473028) and the Research Foundation of State Key Laboratory of Systems Medicine for Cancer (ZZ‐94‐25‐18 and ZZ‐94‐25‐19).

## Conflicts of Interest

The authors declare no conflicts of interest.

## Supporting information




**Supporting File 1**: advs77502‐sup‐0001‐SuppMat.docx.

**Supporting File 2**: advs77502‐sup‐0002‐FigureS17.pdf.

## Data Availability

The publicly available datasets analyzed in this study are described in the Methods. The data supporting the findings of this study are available within the article and its Supporting Information or from the corresponding author upon reasonable request.
