# Supplementary material for: The TUBA1B–G3BP2 Axis Sustains p65 Transcriptional Activity to Promote Hepatocellular Carcinoma Progression
Source: Adv Sci (Weinh). 2026 Sep 6:e77502. Online ahead of print. doi: 10.1002/advs.77502 (PMC13547788; doi:10.1002/advs.77502)
Supplement: Supplementary file 1 [file ADVS-9999-e77502-s002.pdf]

Fig.S17-1

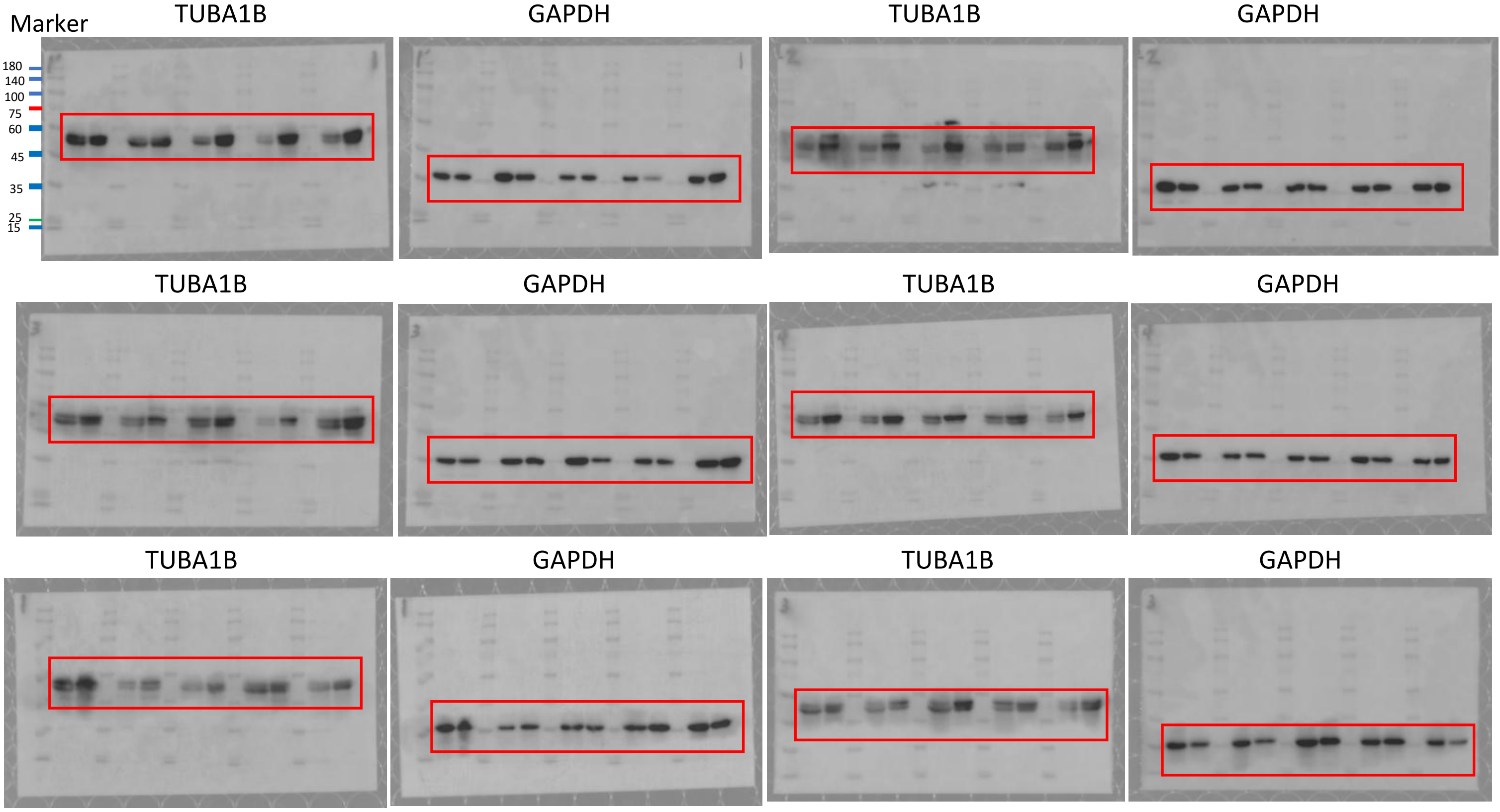

Fig.S17-1 Original blots corresponding to Fig.1F

Fig.S17-2

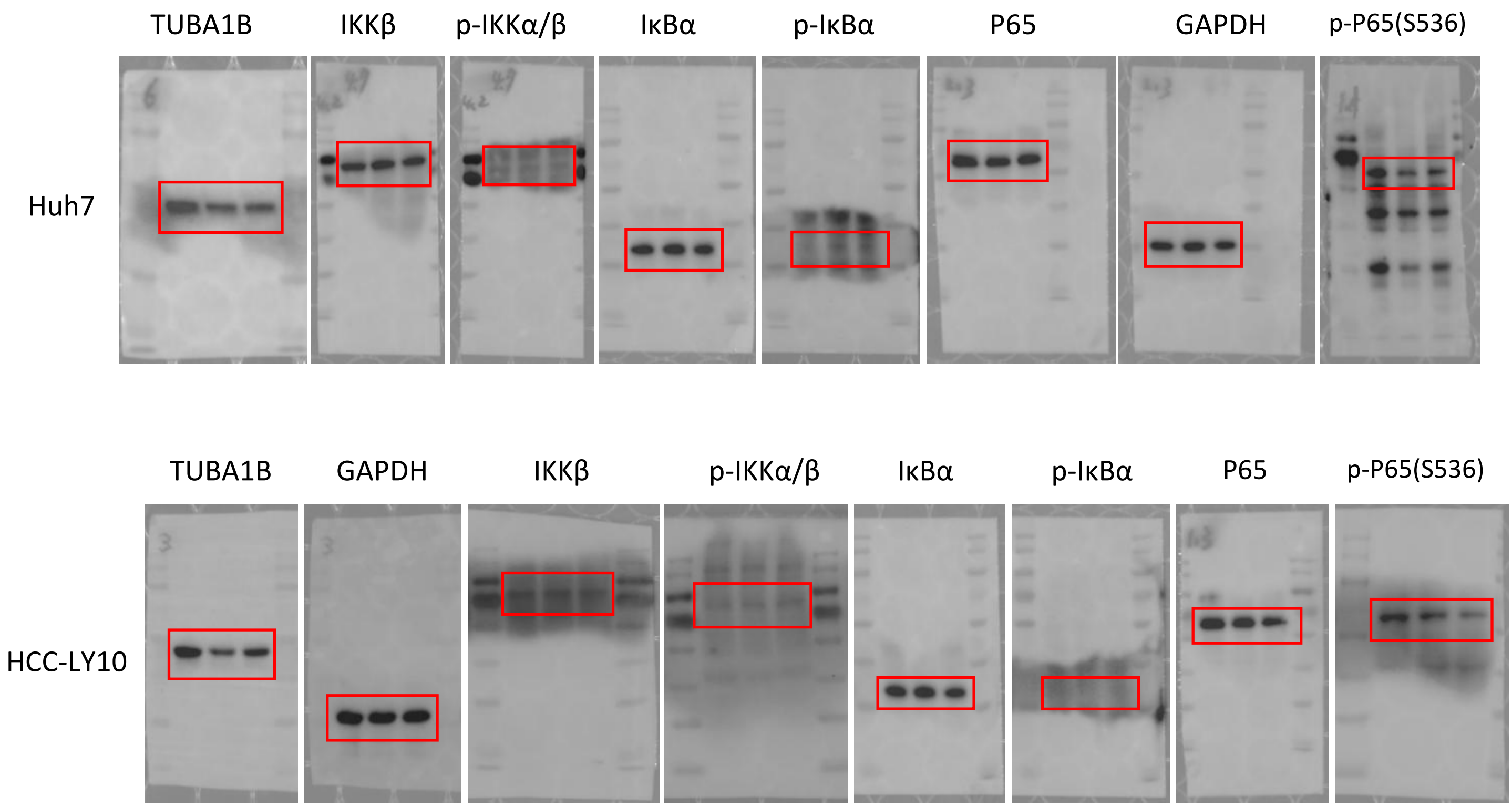

Fig.S17-2 Original blots corresponding to Fig.3D

Fig.S17-3

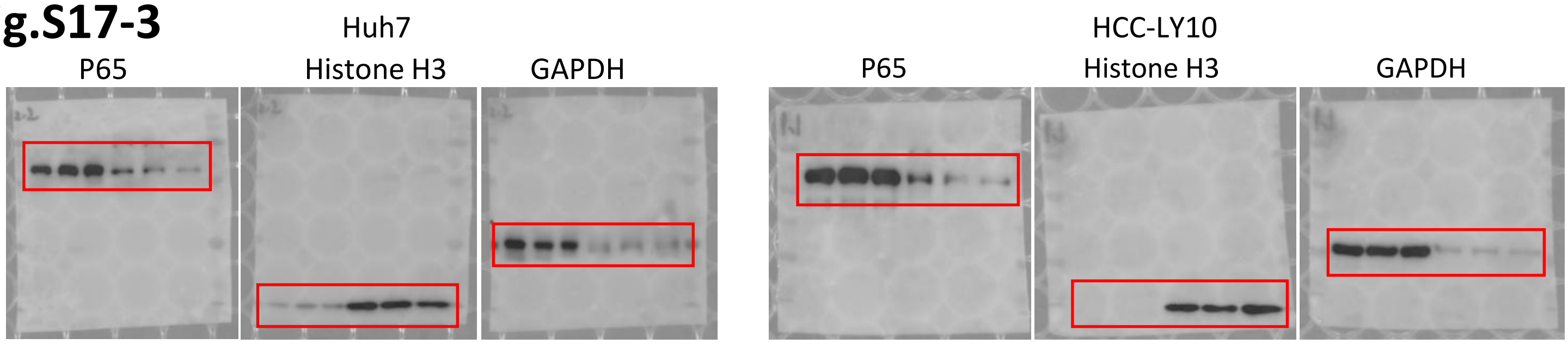

Fig.S17-3 Original blots corresponding to Fig.3E

Fig.S17-4

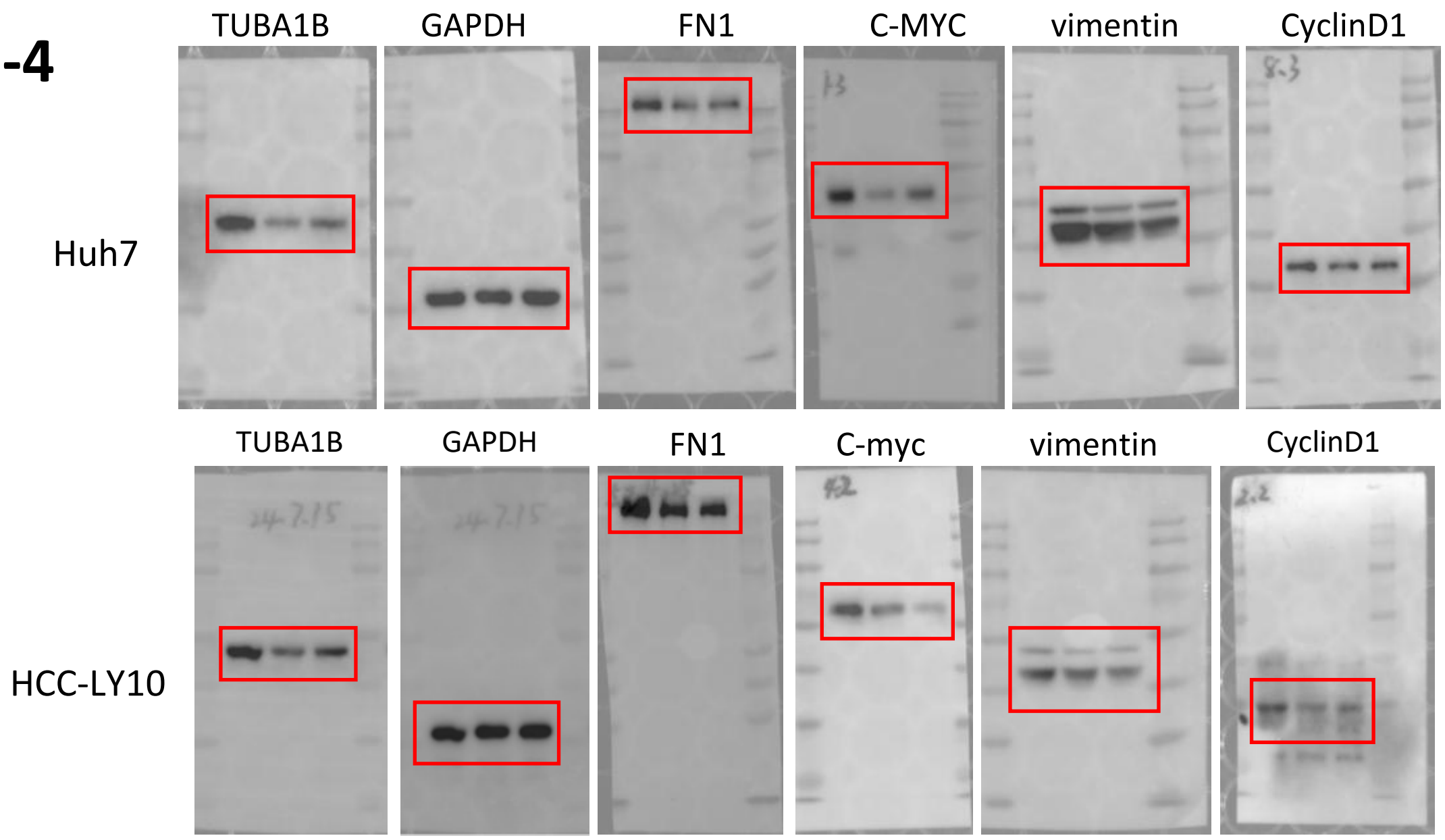

Fig.S17-4 Original blots corresponding to Fig.3H

Fig.S17-5

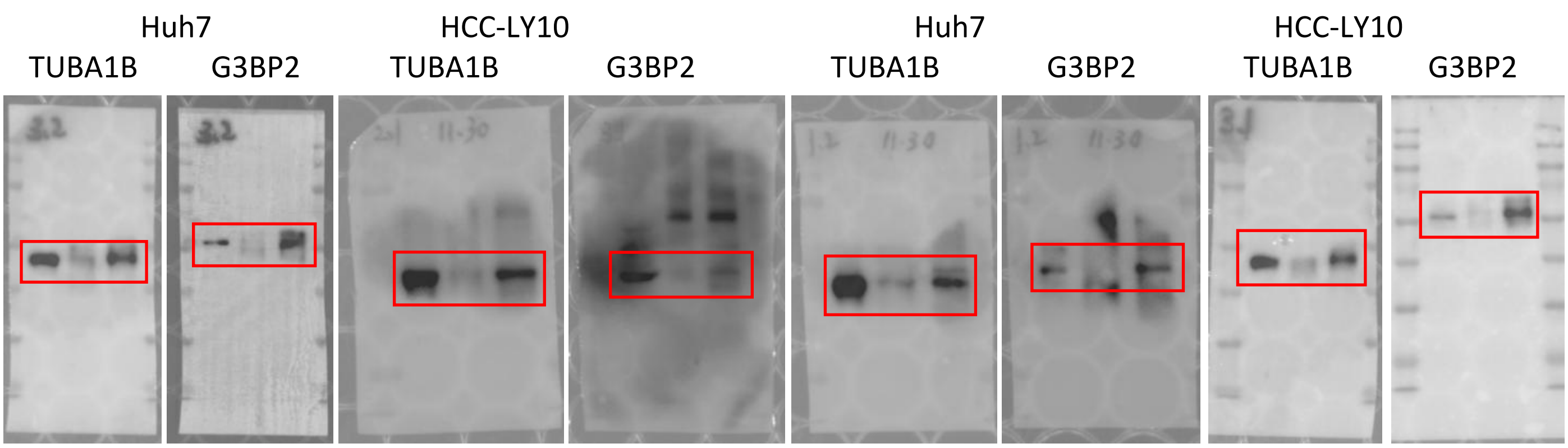

Fig.S17-5 Original blots corresponding to Fig.4B

Fig.S17-6

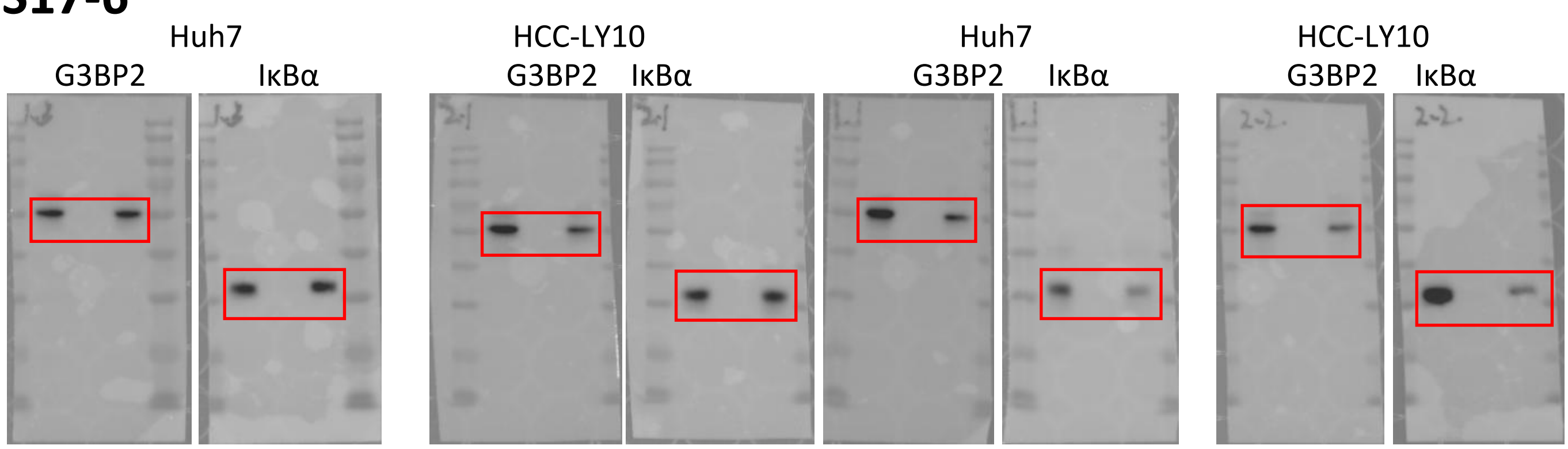

Fig.S17-6 Original blots corresponding to Fig.4D

Fig.S17-7

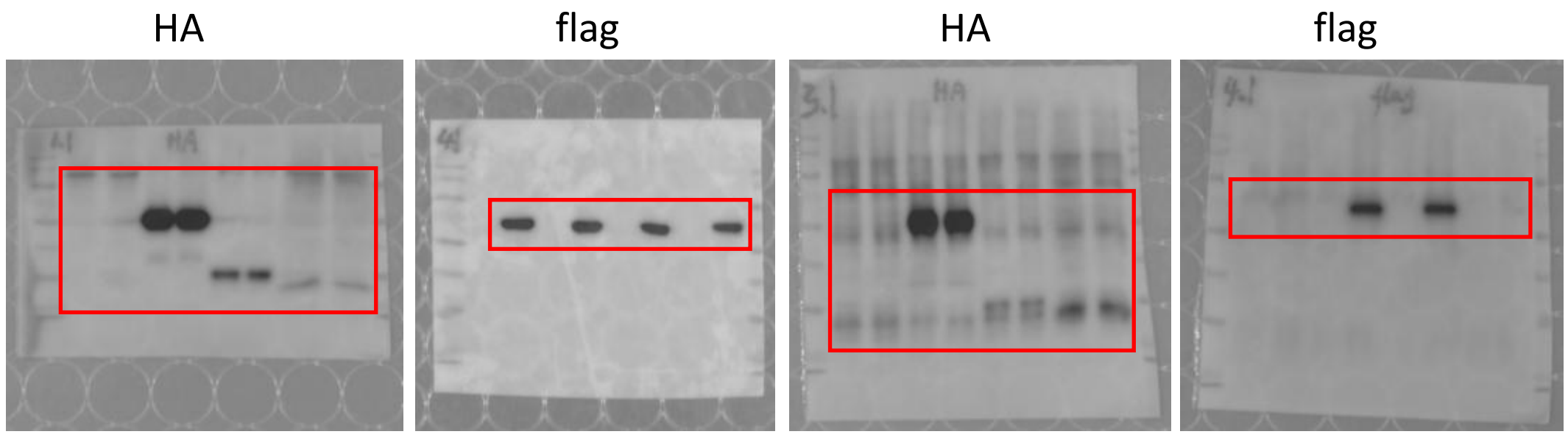

Fig.S17-7 Original blots corresponding to Fig.4G

Fig.S17-8

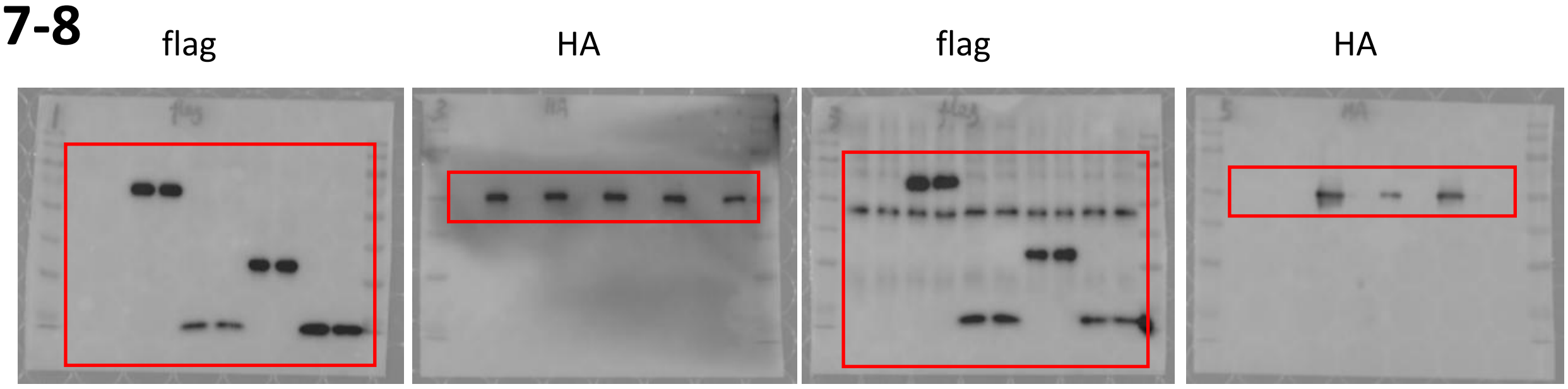

Fig.S17-8 Original blots corresponding to Fig.4H

Fig.S17-9

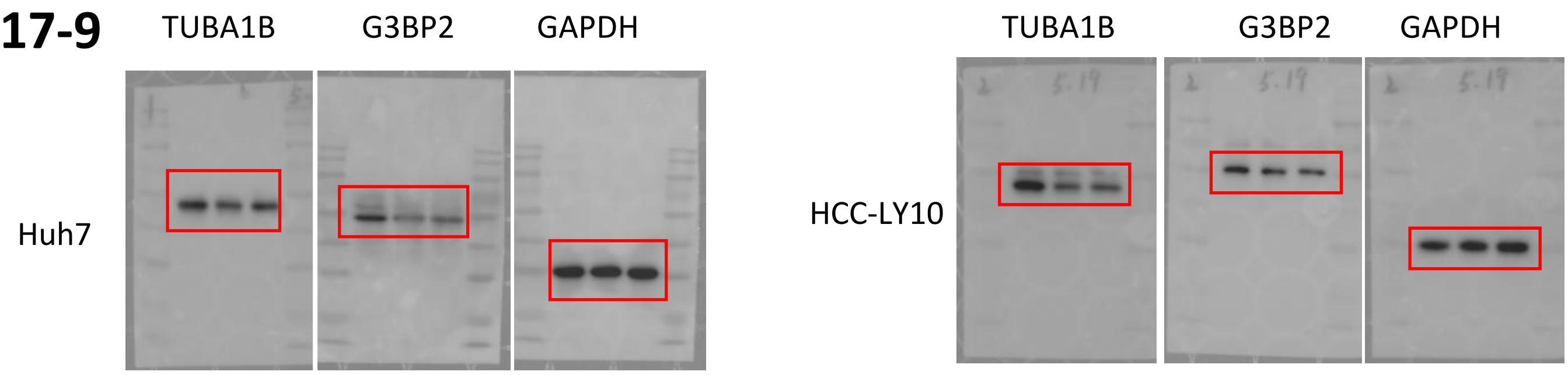

Fig.S17-9 Original blots corresponding to Fig.4J

Fig.S17-10

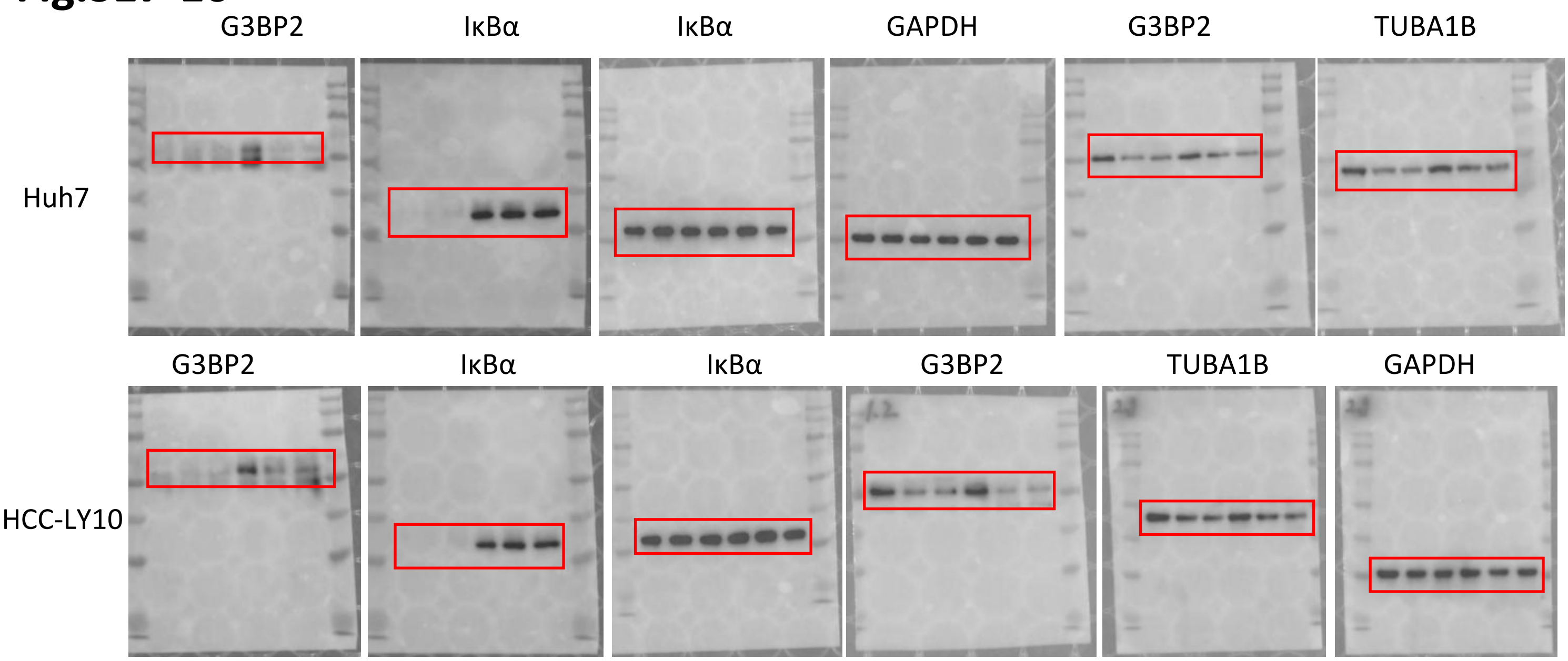

Fig.S17-10 Original blots corresponding to Fig.4K

Fig.S17-11

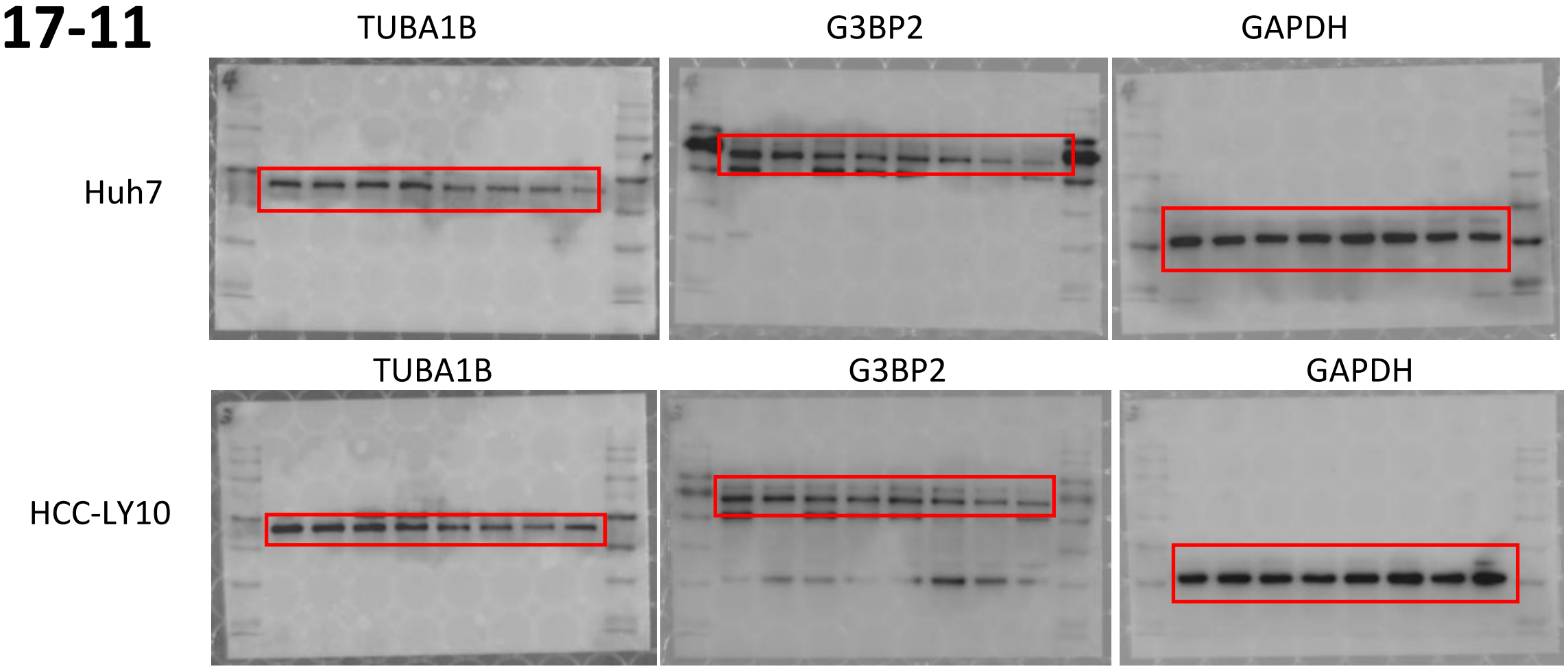

Fig.S17-11 Original blots corresponding to Fig.5A

Fig.S17-12

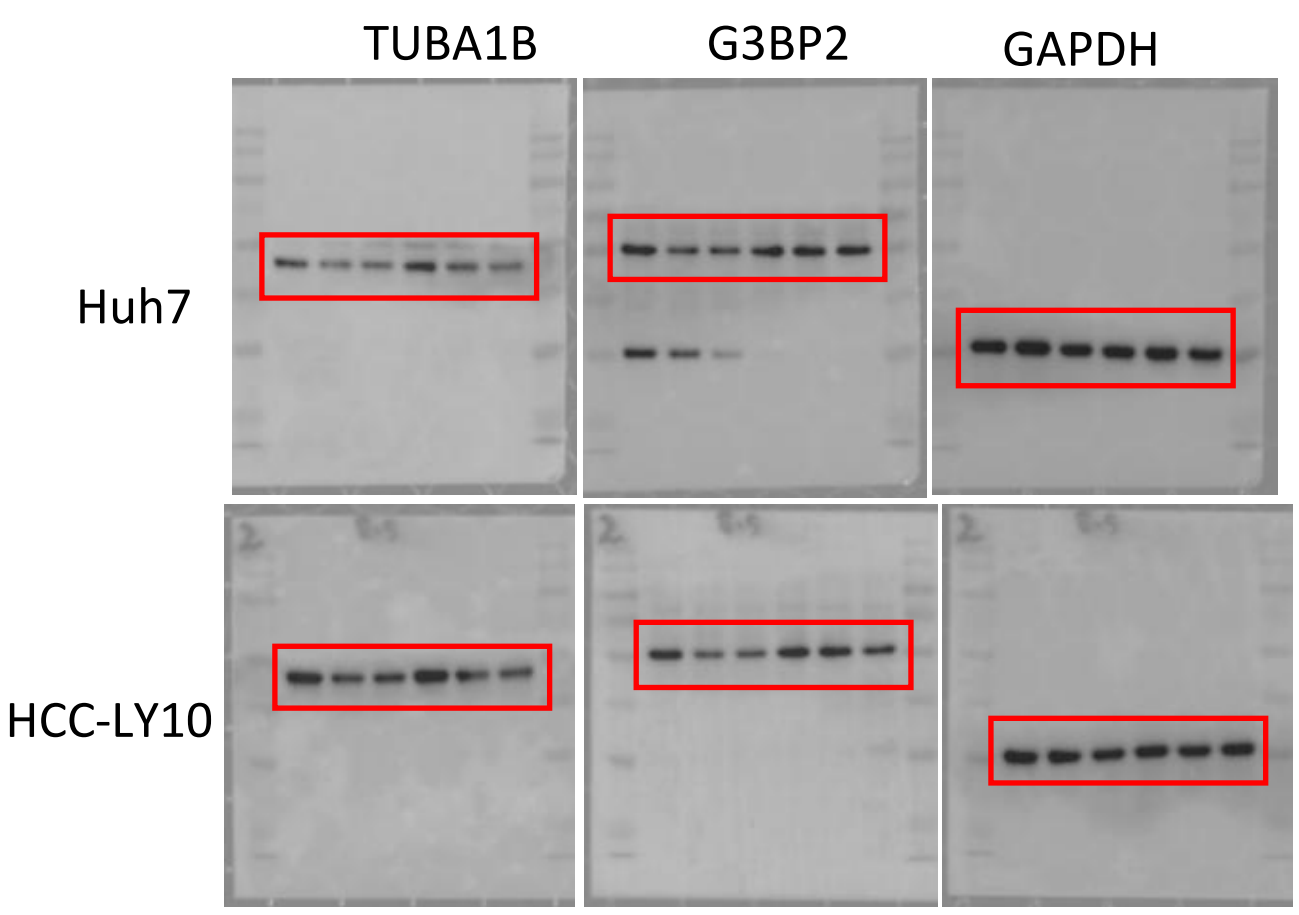

Fig.S17-12 Original blots corresponding to Fig.5B

Fig.S17-13

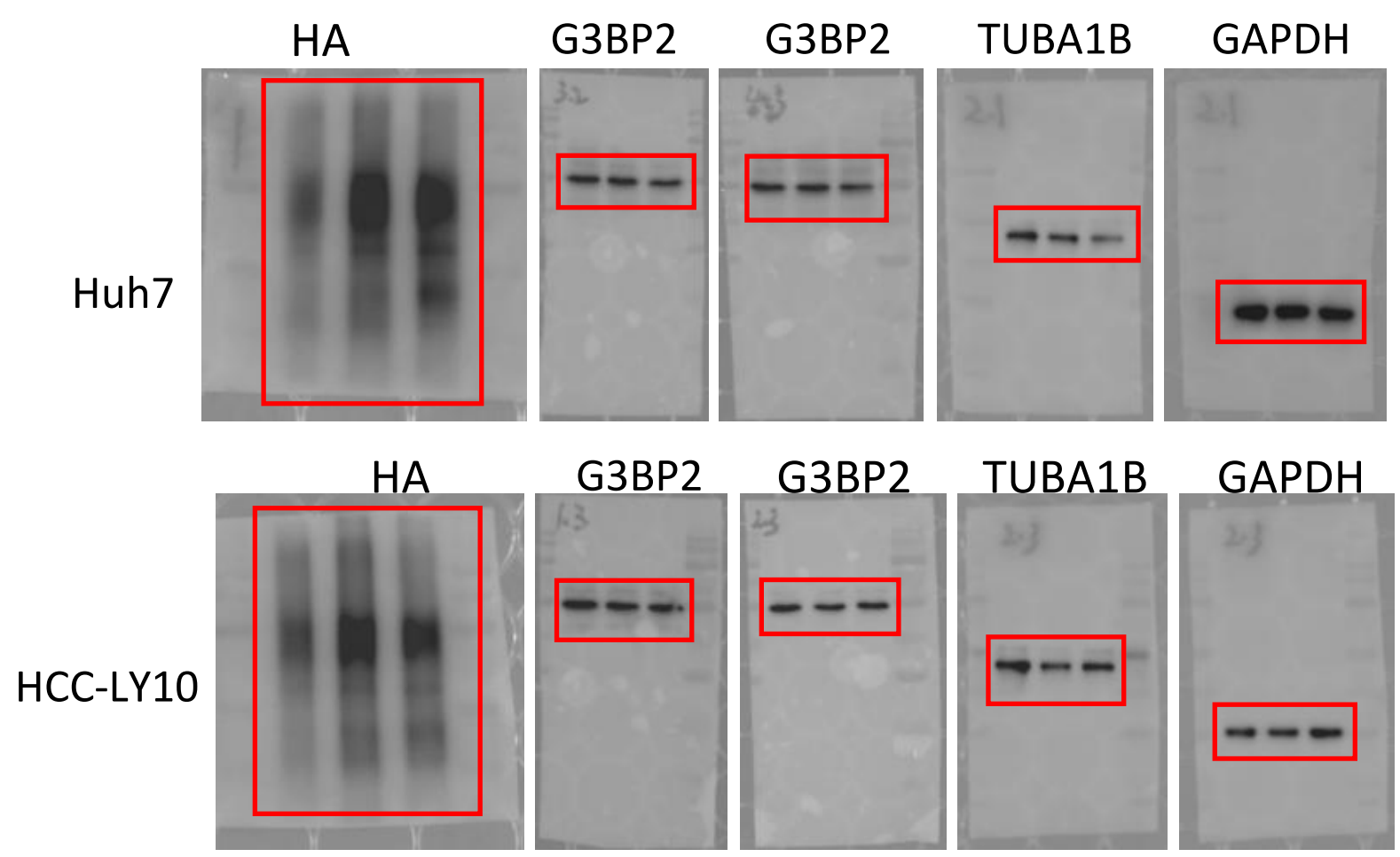

Fig.S17-13 Original blots corresponding to Fig.5C

Fig.S17-14

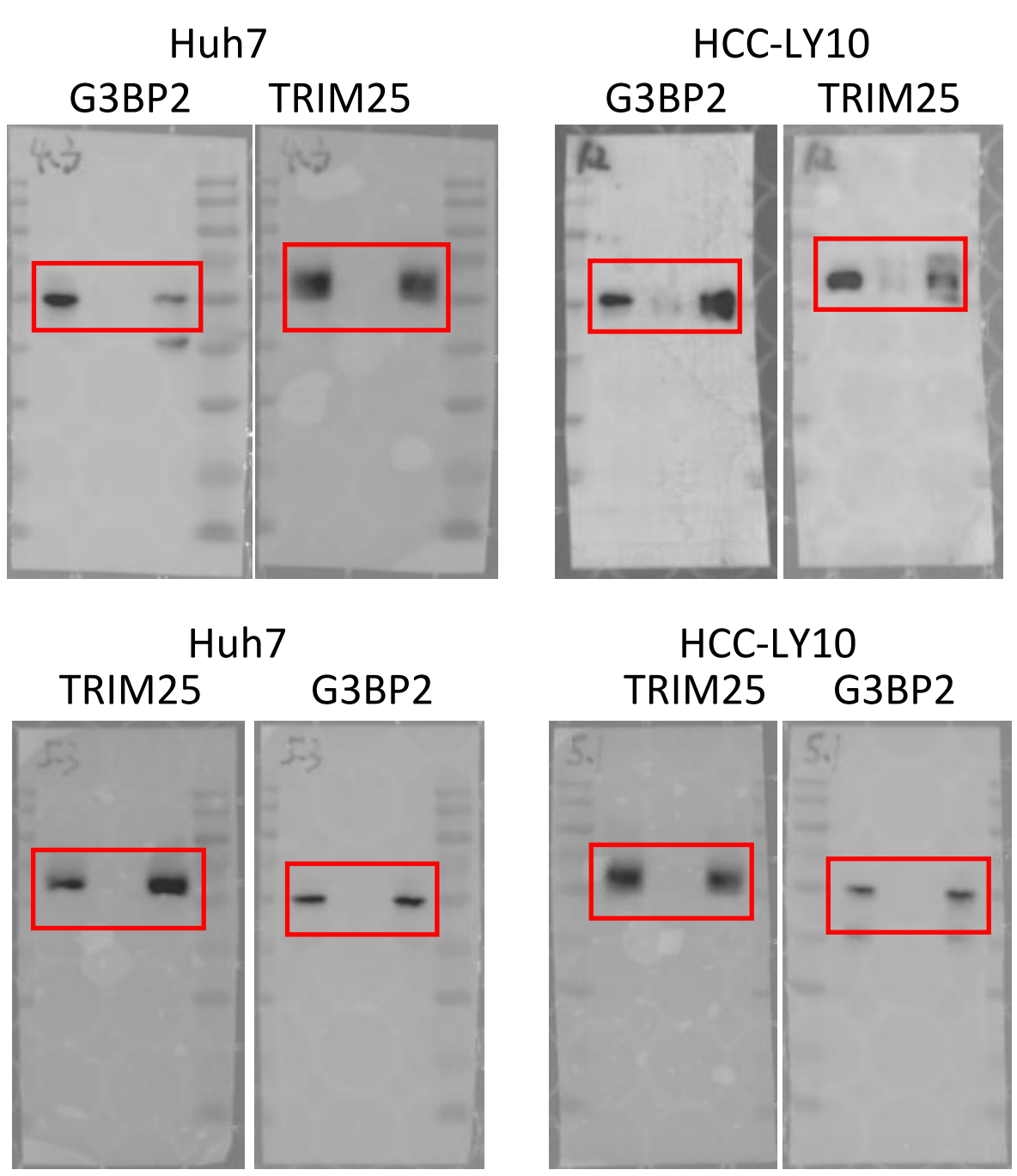

Fig.S17-14 Original blots corresponding to Fig.5D

Fig.S17-15

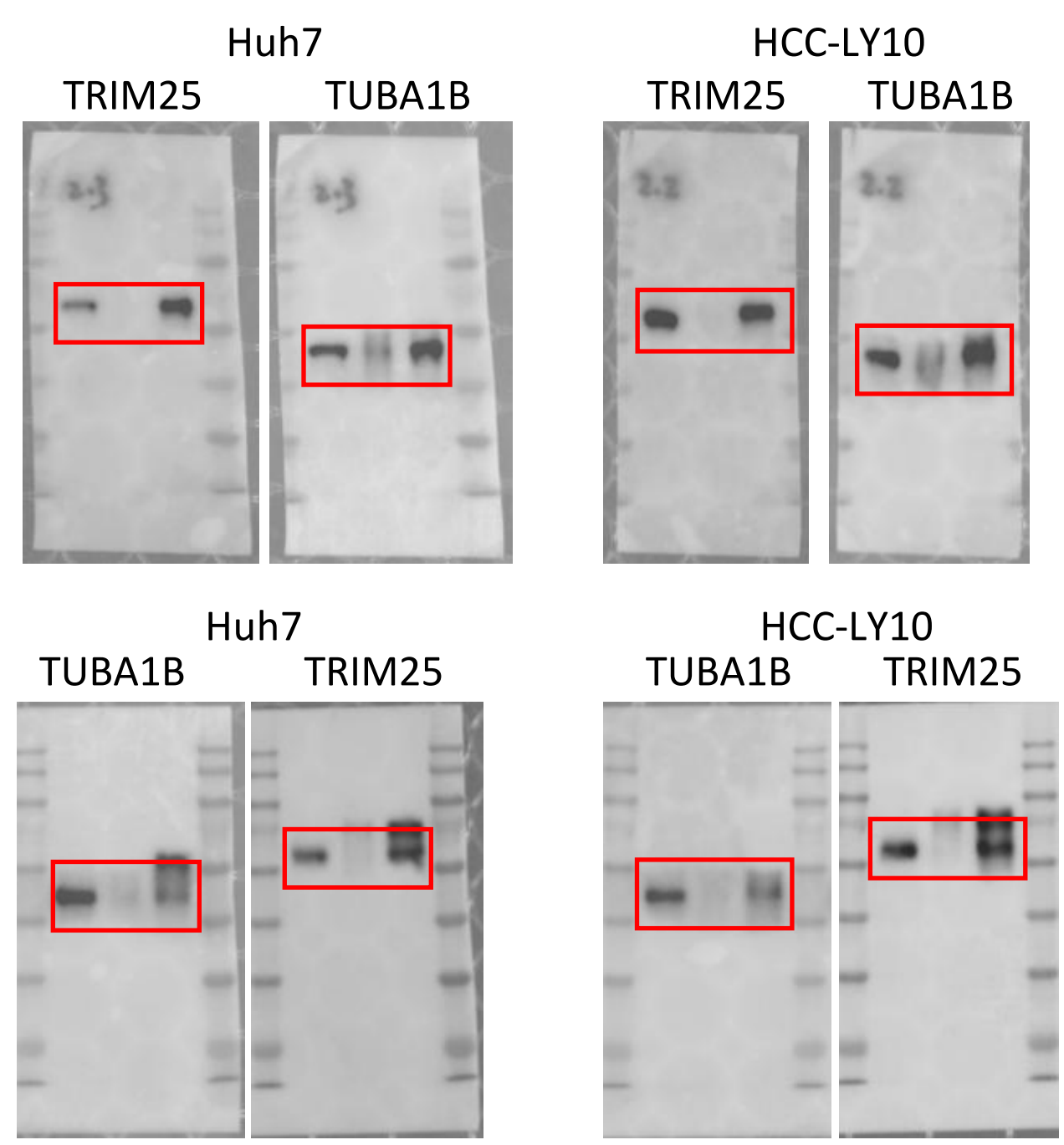

Fig.S17-15 Original blots corresponding to Fig.5E

Fig.S17-16

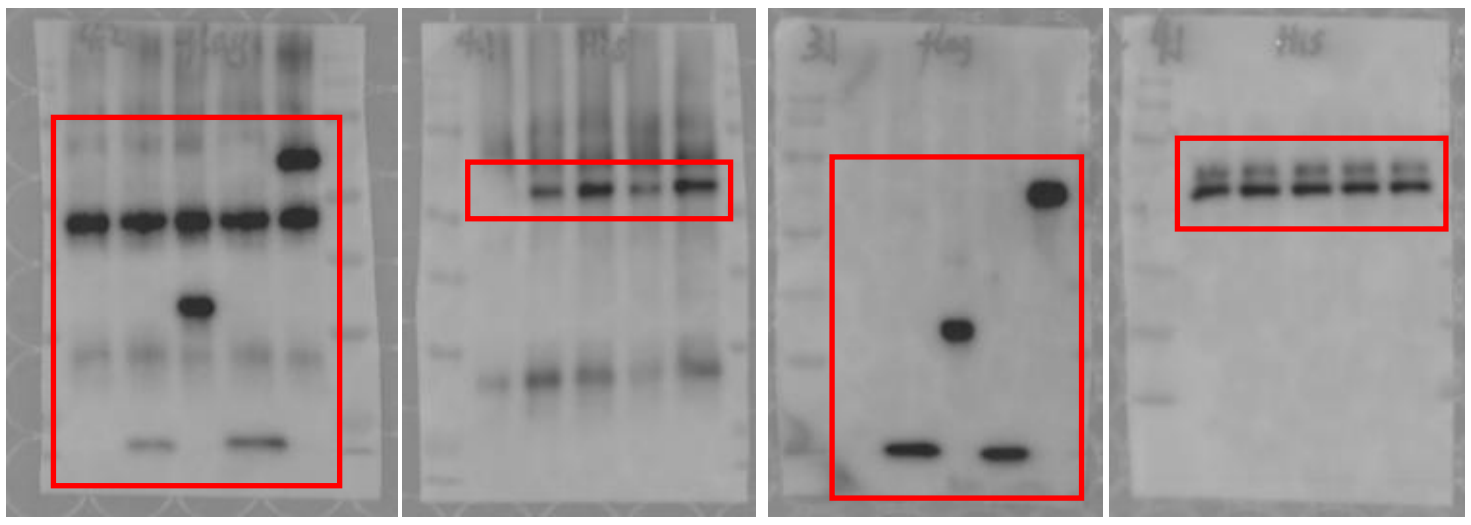

Fig.S17-16 Original blots corresponding to Fig.5F

Fig.S17-17

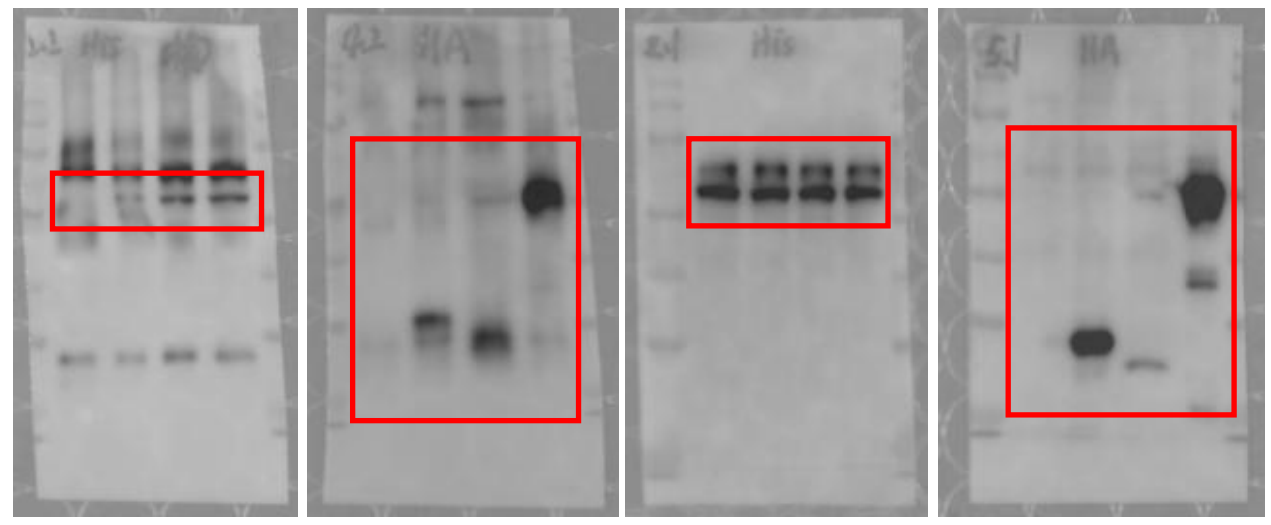

Fig.S17-17 Original blots corresponding to Fig.5G

Fig.S17-18

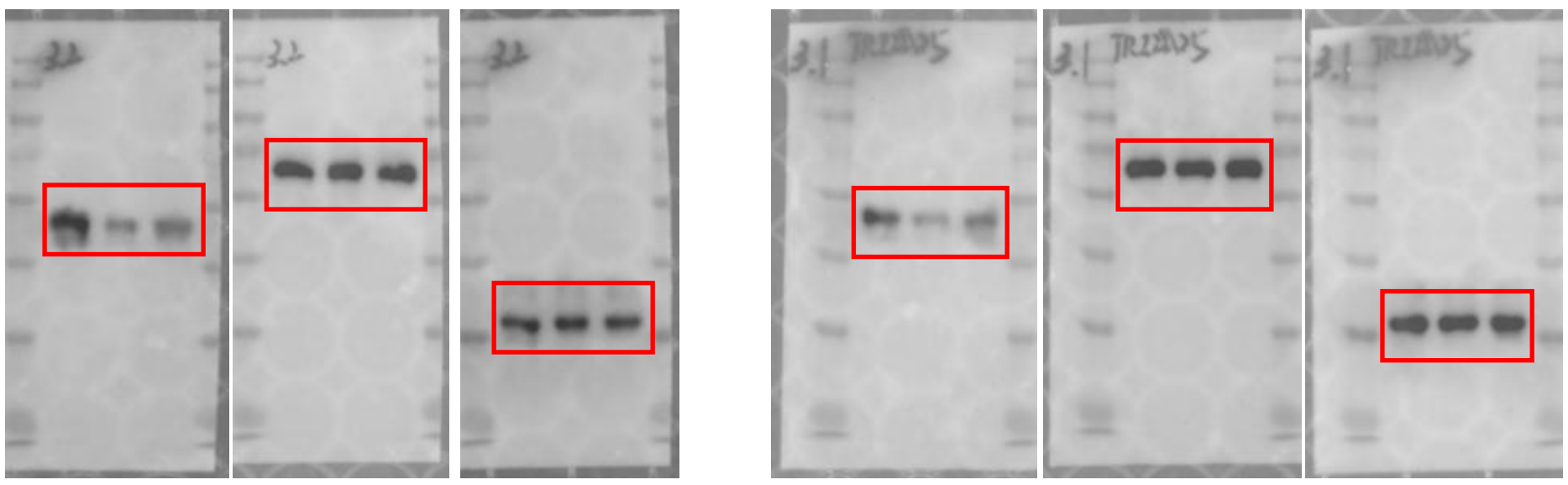

Fig.S17-18 Original blots corresponding to Fig.5H

Fig.S17-19

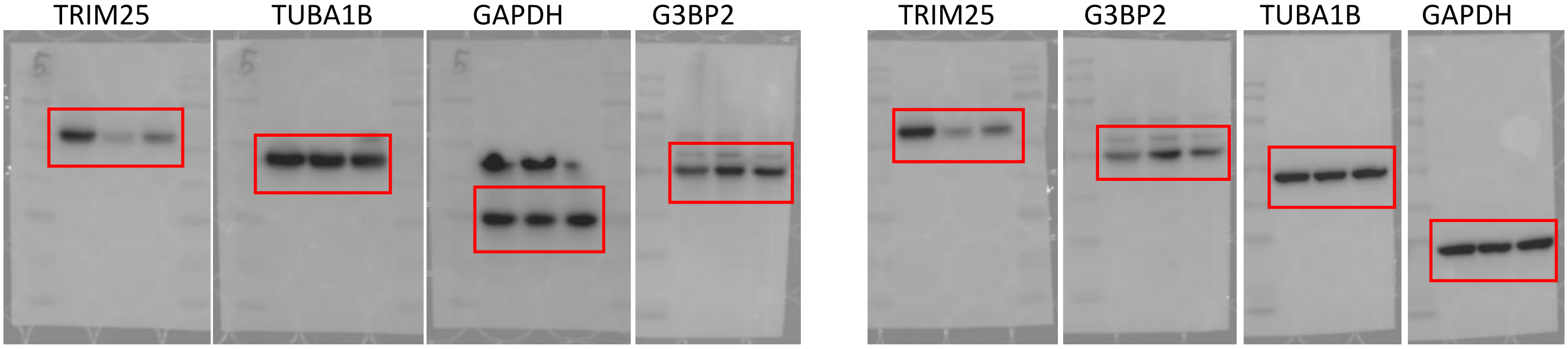

Fig.S17-19 Original blots corresponding to Fig.5I

Fig.S17-20

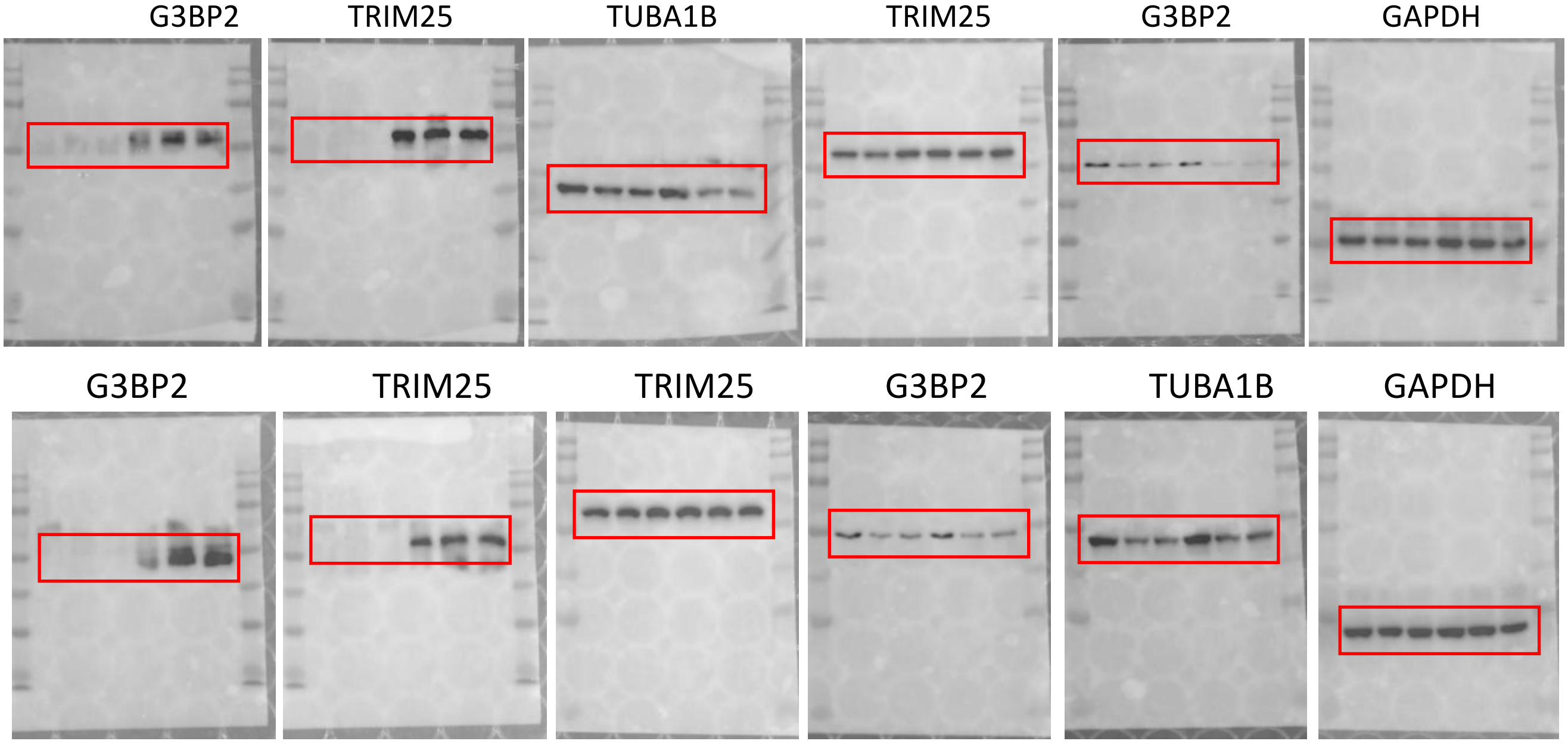

Fig.S17-20 Original blots corresponding to Fig.5J

Fig.S17-21

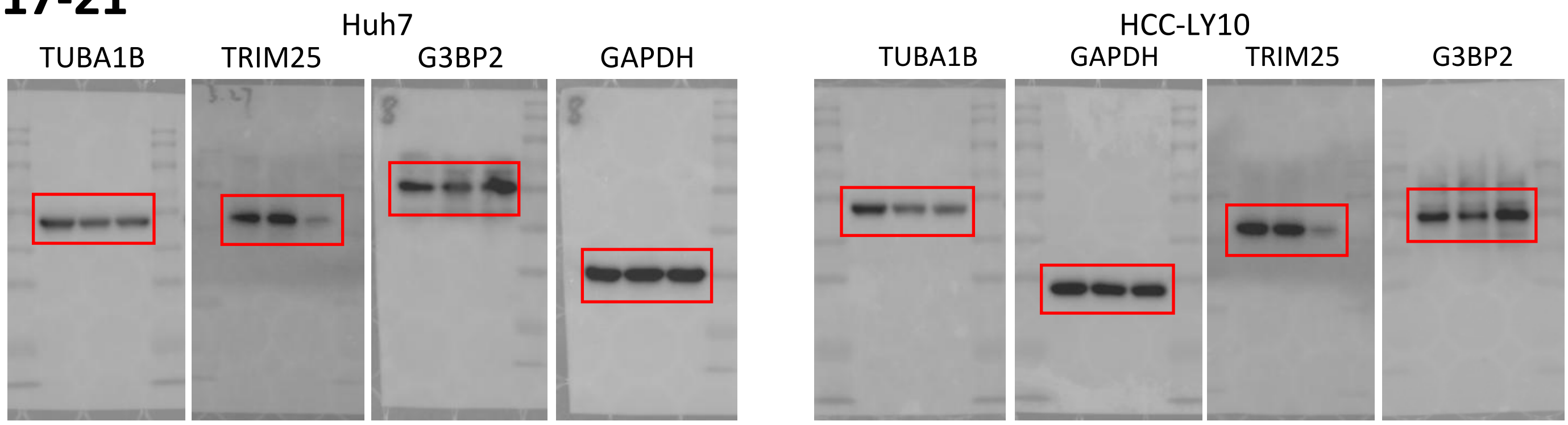

Fig.S17-21 Original blots corresponding to Fig.5K

Fig.S17-22

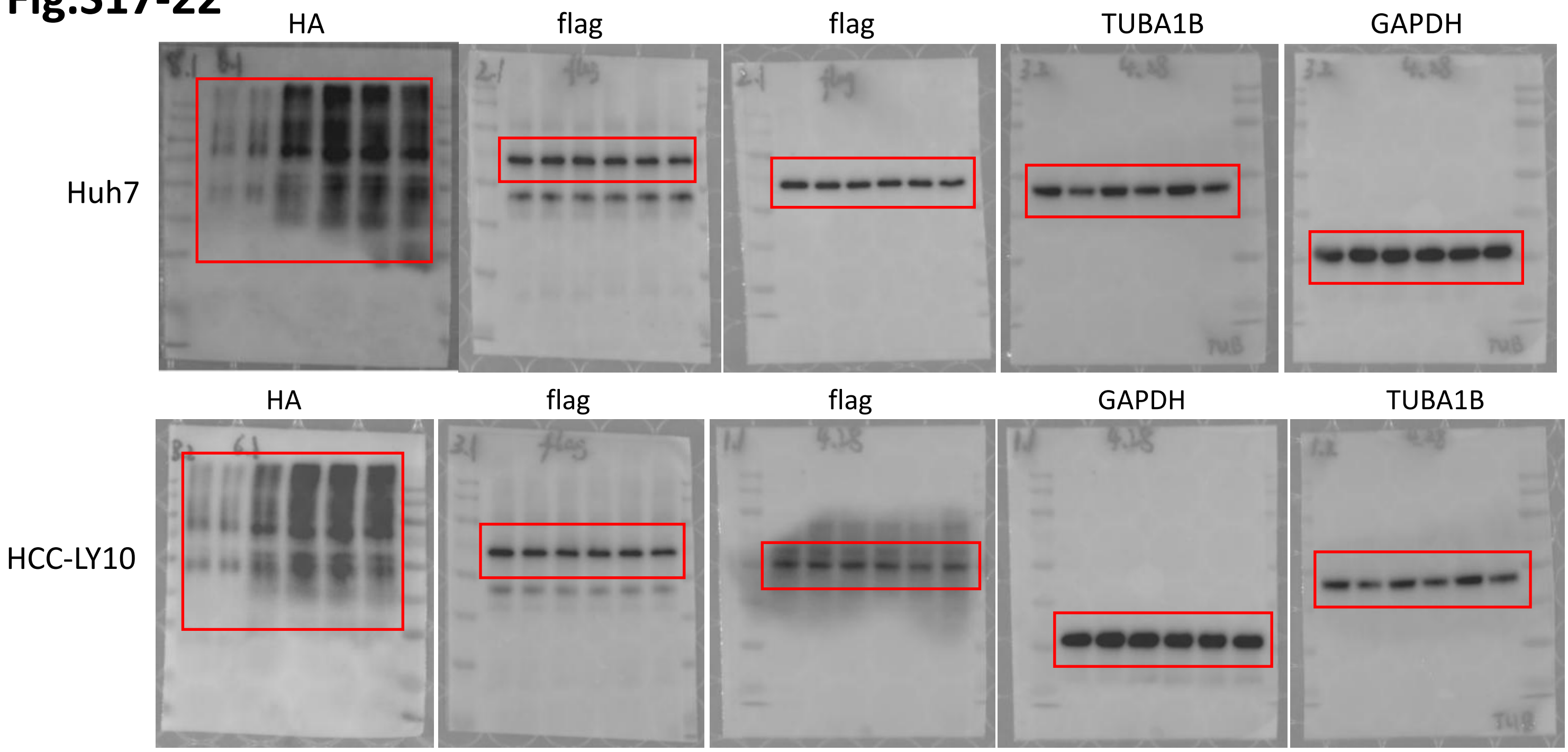

Fig.S17-22 Original blots corresponding to Fig.5L

Fig.S17-23

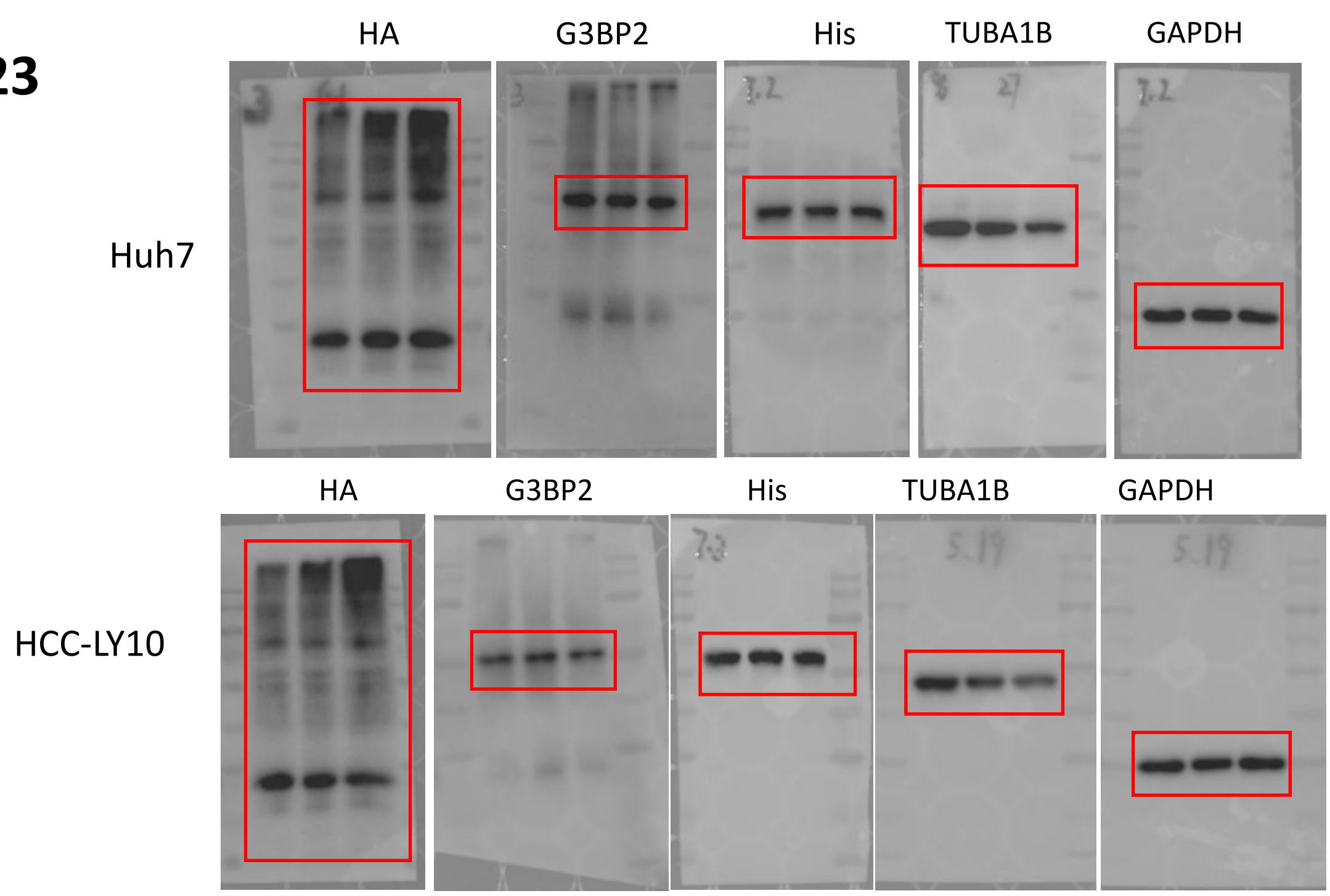

Fig.S17-23 Original blots corresponding to Fig.5M

Fig.S17-24

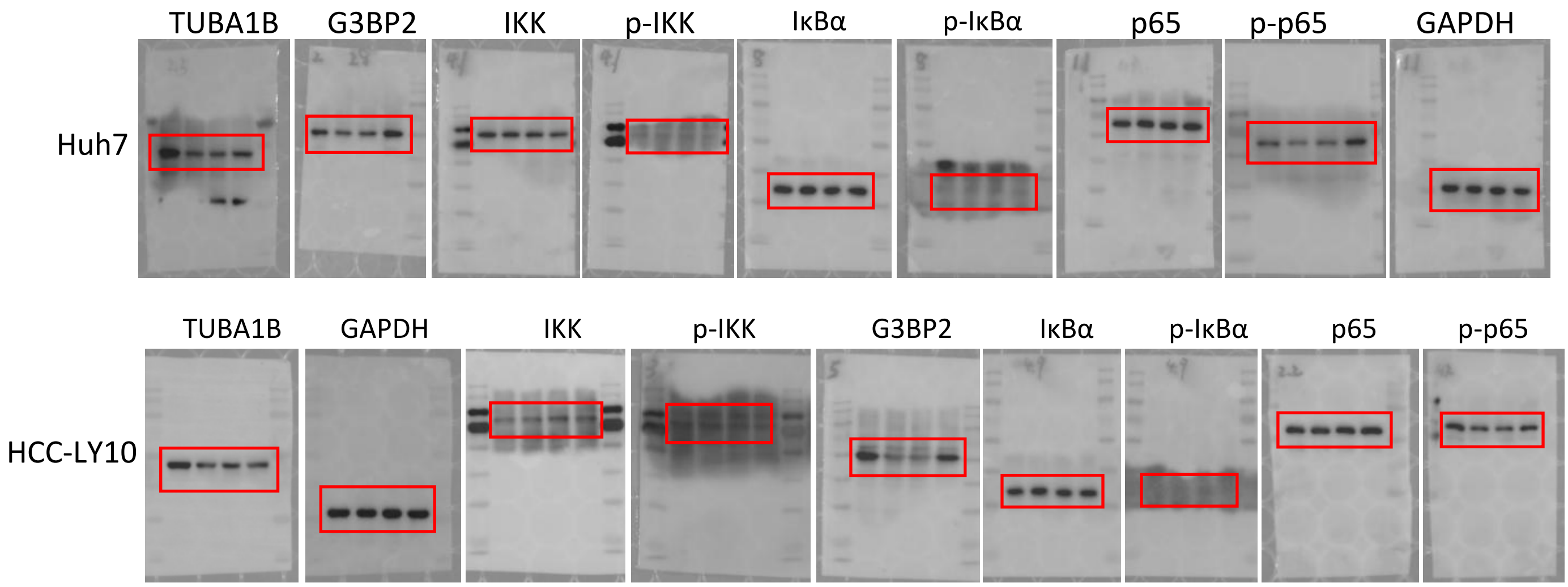

Fig.S17-24 Original blots corresponding to Fig.6H

Fig.S17-25

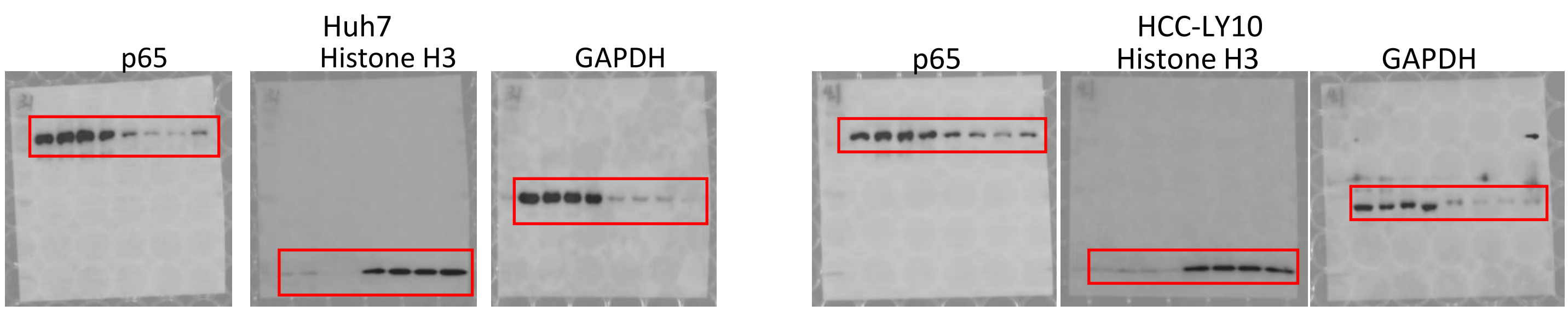

Fig.S17-25 Original blots corresponding to Fig.6I

Fig.S17-26

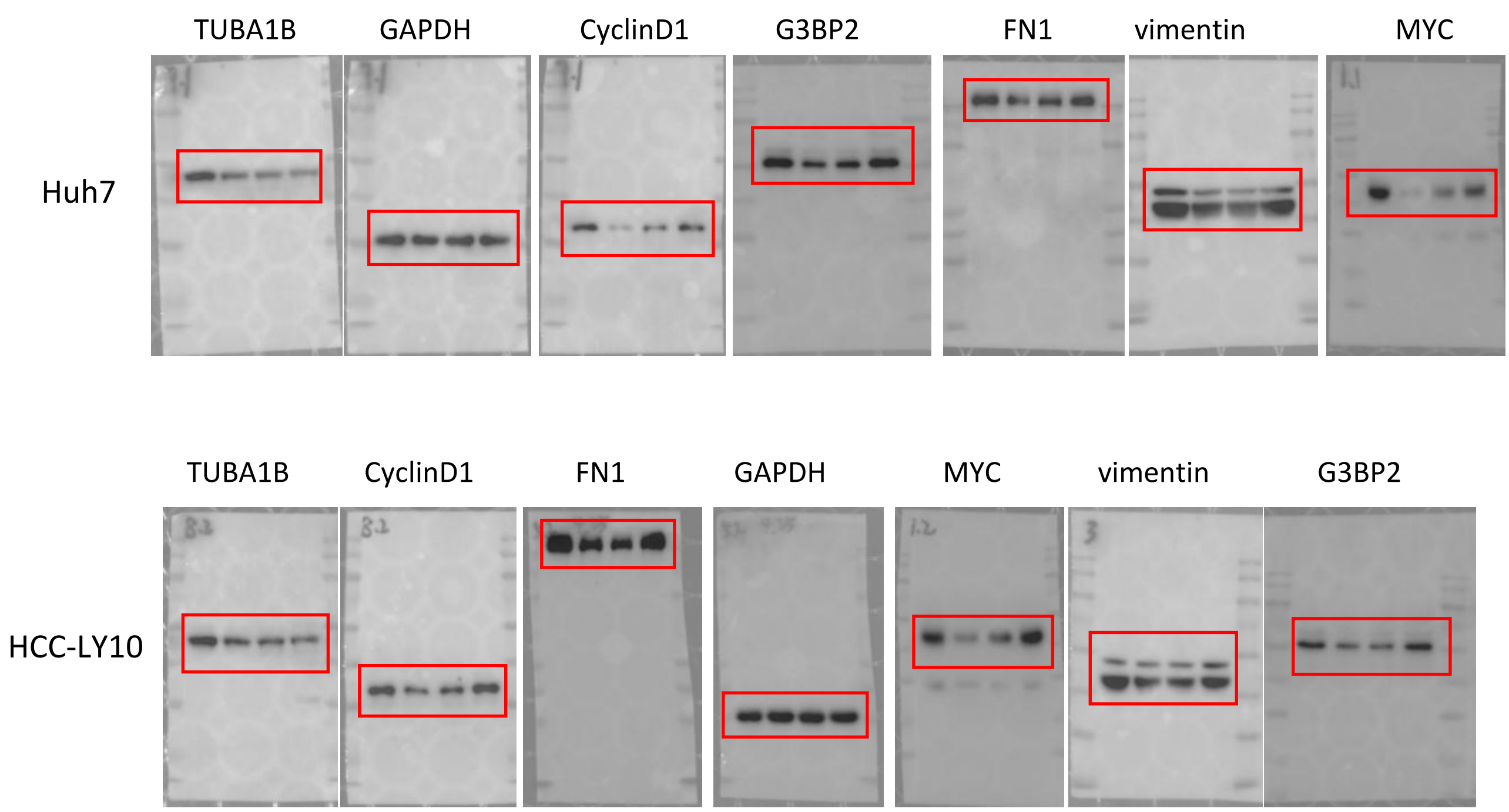

Fig.S17-26 Original blots corresponding to Fig.6K

Fig.S17-27

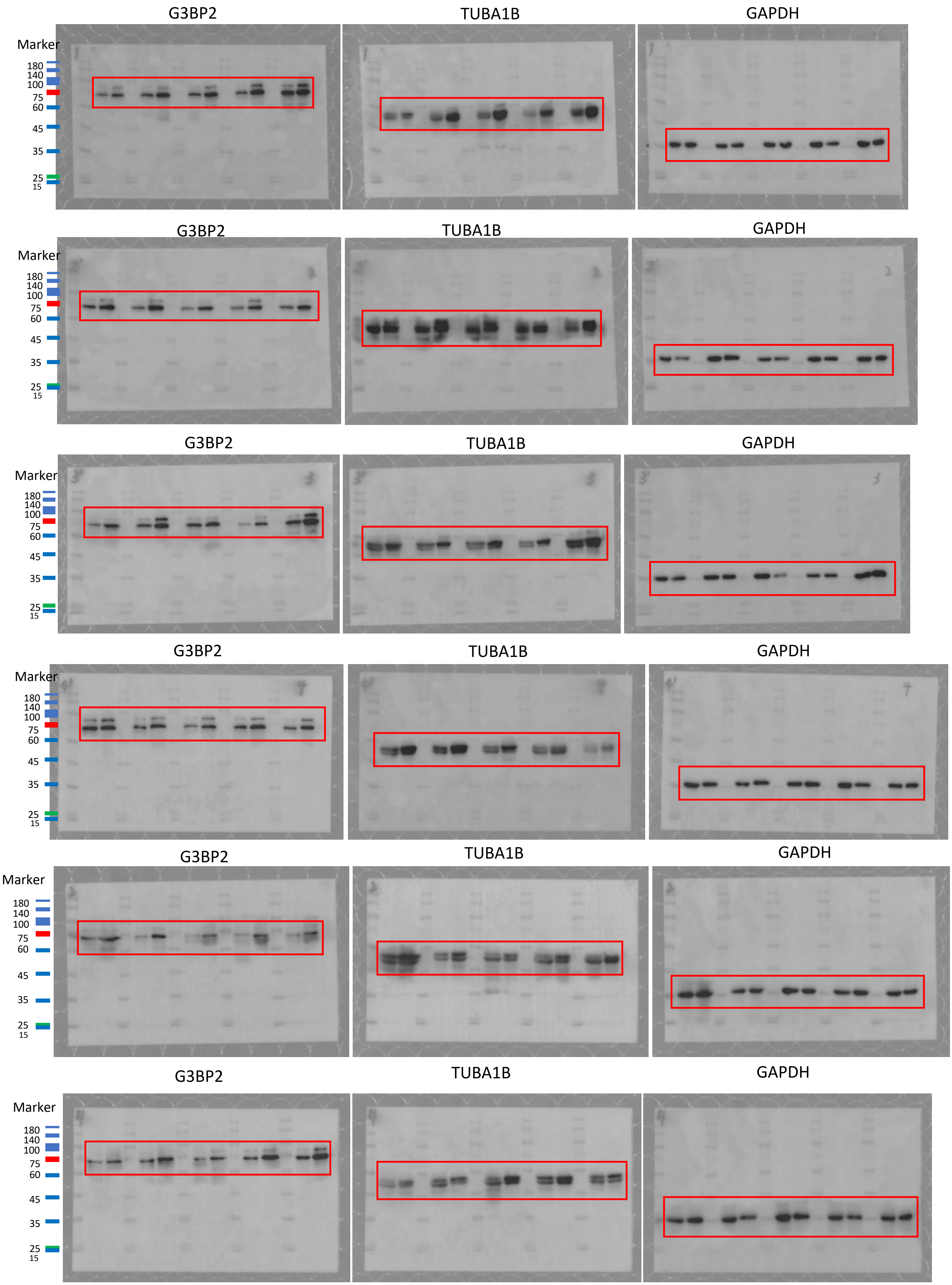

Fig.S17-27 Original blots corresponding to Fig.7A

Fig.S17-28

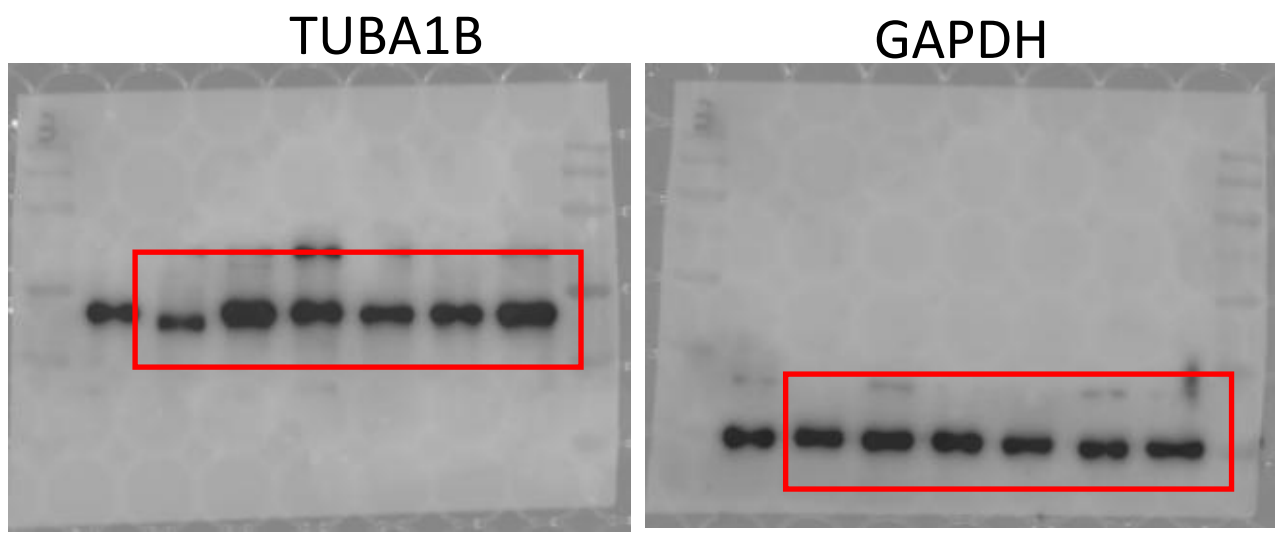

Fig.S17-28 Original blots corresponding to Fig.S3D

Fig.S17-29

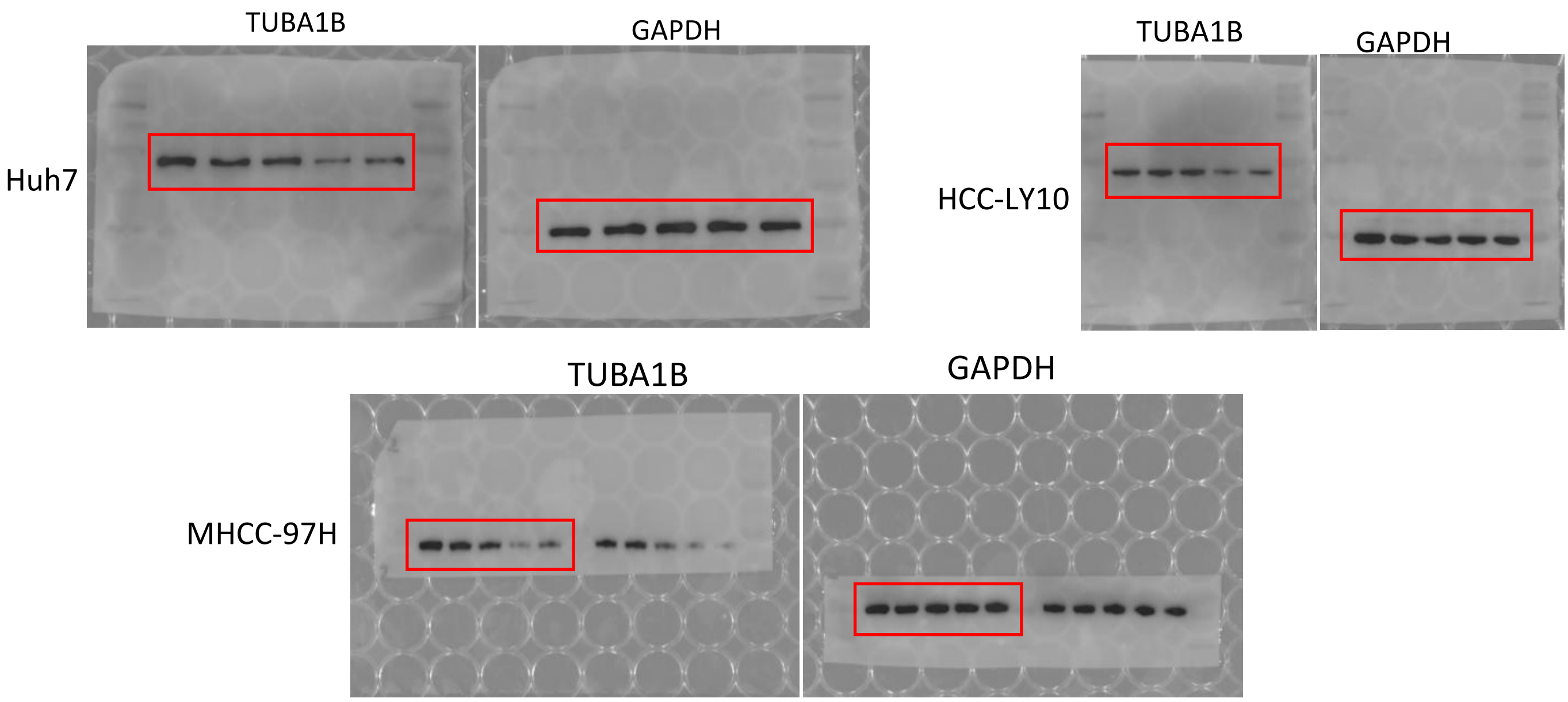

Fig.S17-29 Original blots corresponding to Fig.S4B

Fig.S17-30

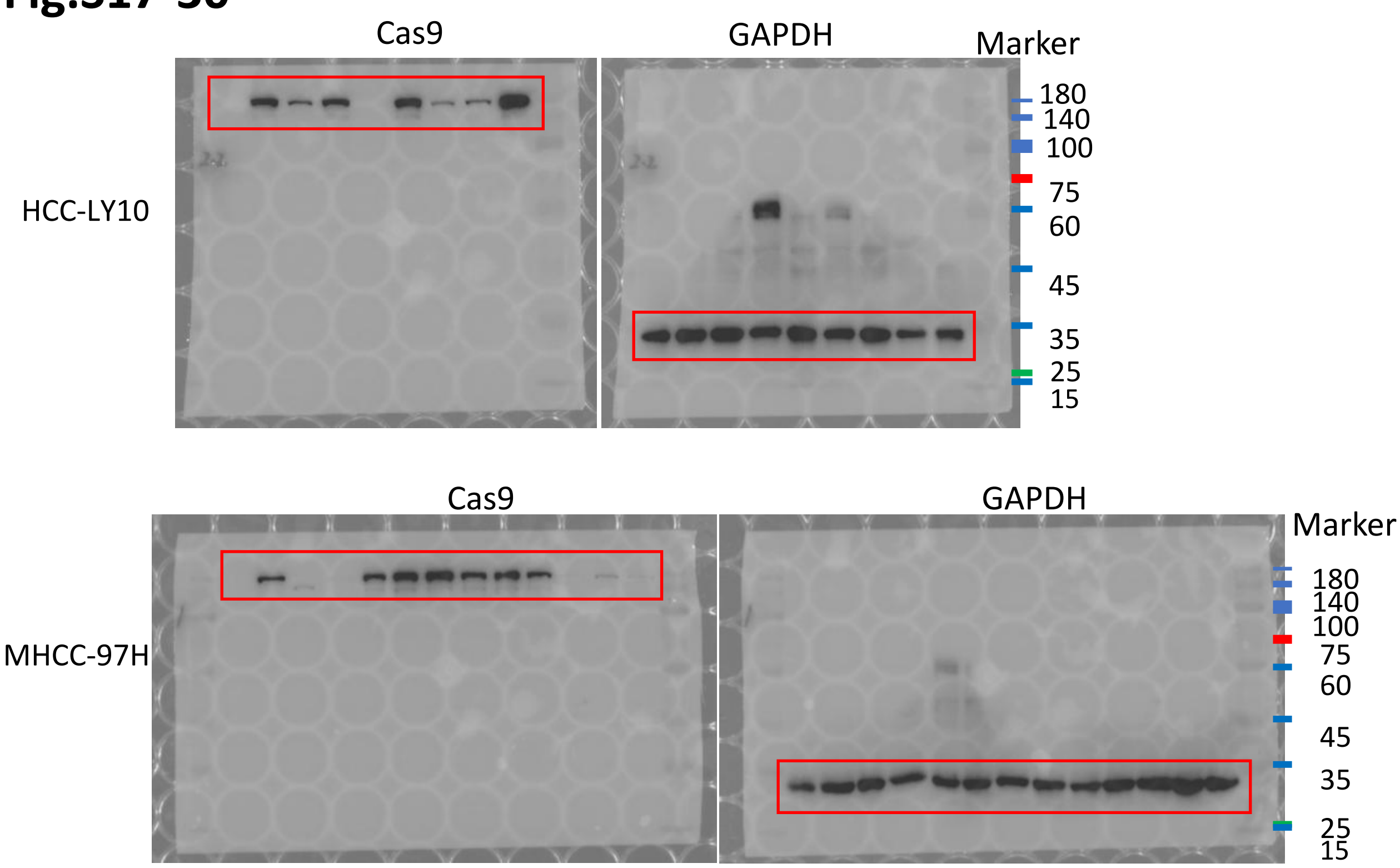

Fig.S17-30 Original blots corresponding to Fig.S4D

Fig.S17-31

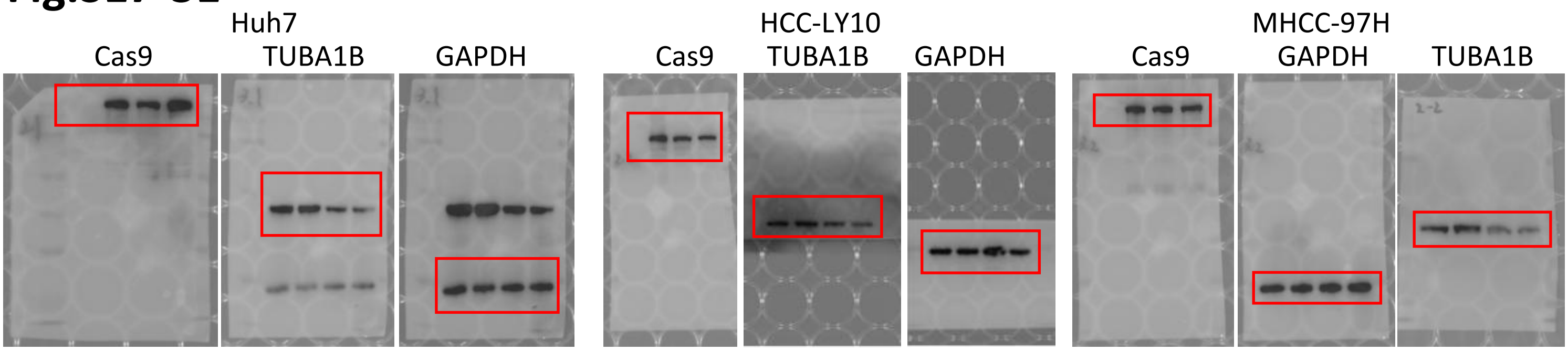

Fig.S17-31 Original blots corresponding to Fig.S4E

Fig.S17-32

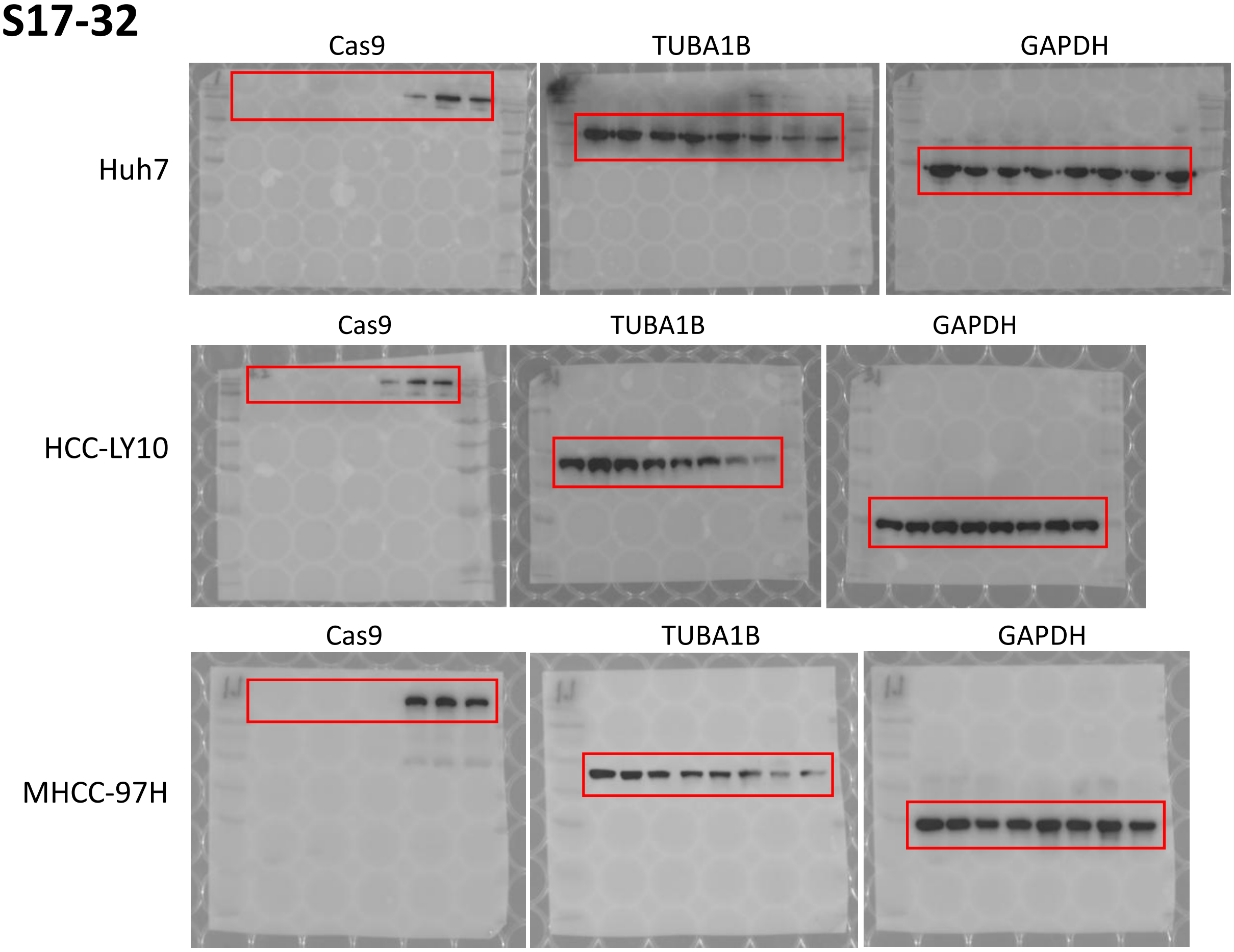

Fig.S17-32 Original blots corresponding to Fig.S4F

Fig.S17-33

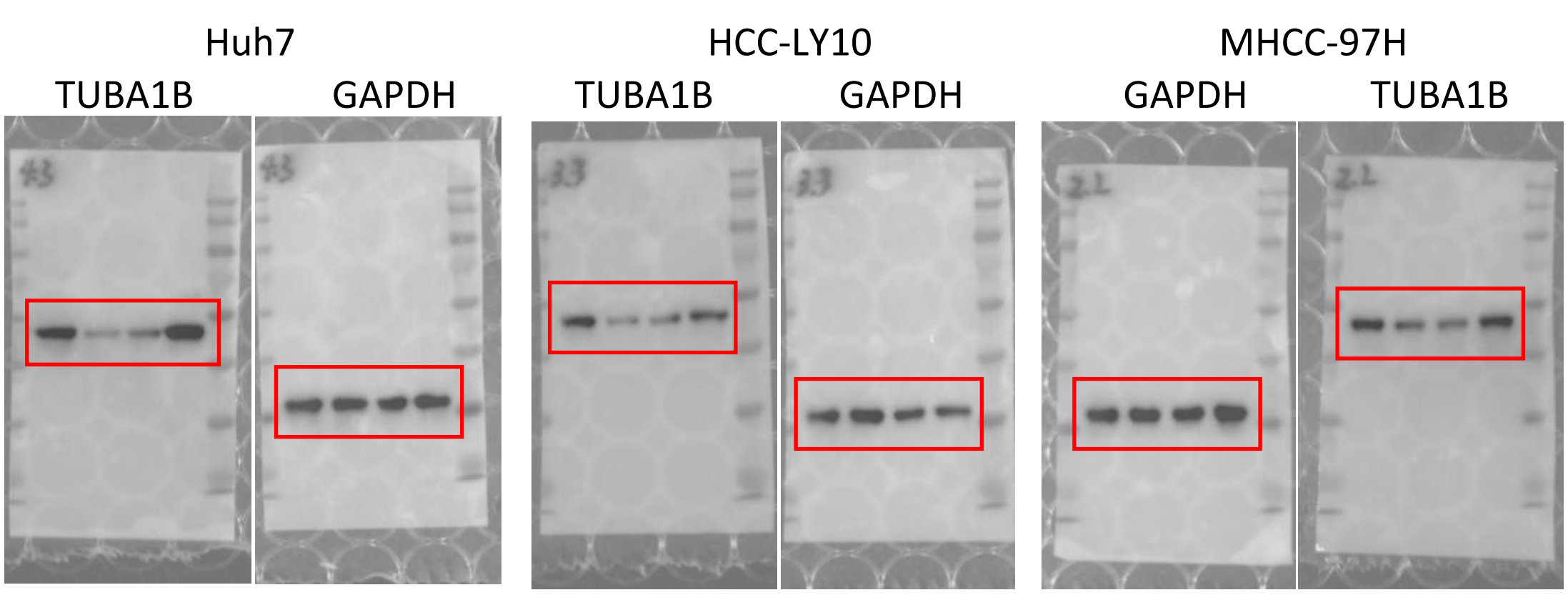

Fig.S17-33 Original blots corresponding to Fig.S8A

Fig.S17-34

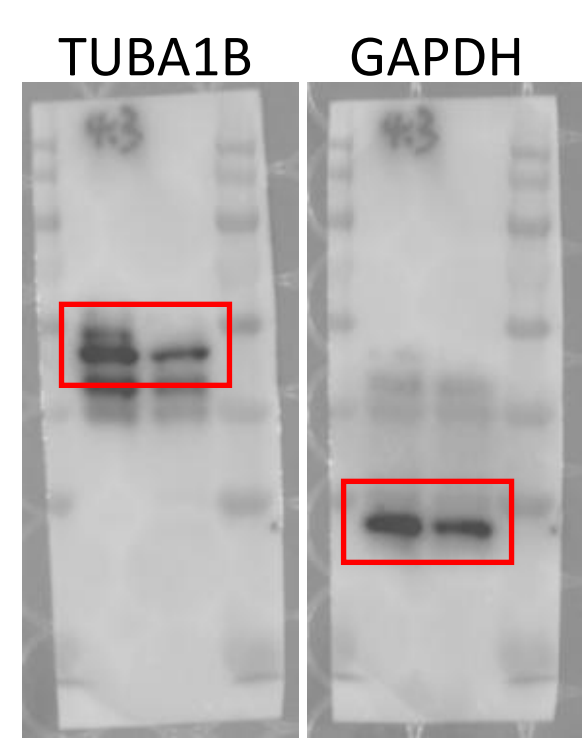

Fig.S17-34 Original blots corresponding to Fig.S8D

Fig.S17-35

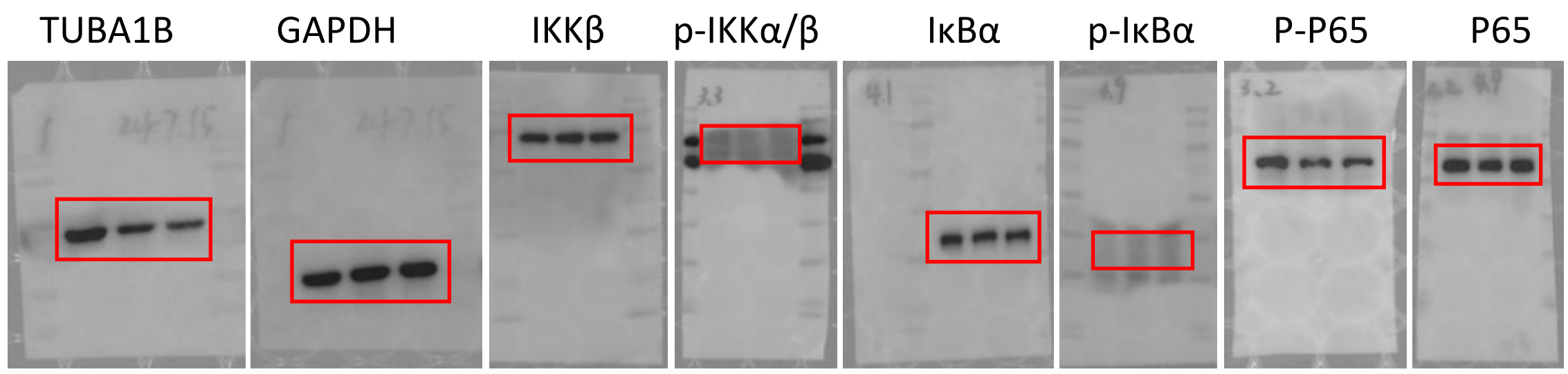

Fig.S17-35 Original blots corresponding to Fig.S9B

Fig.S17-36

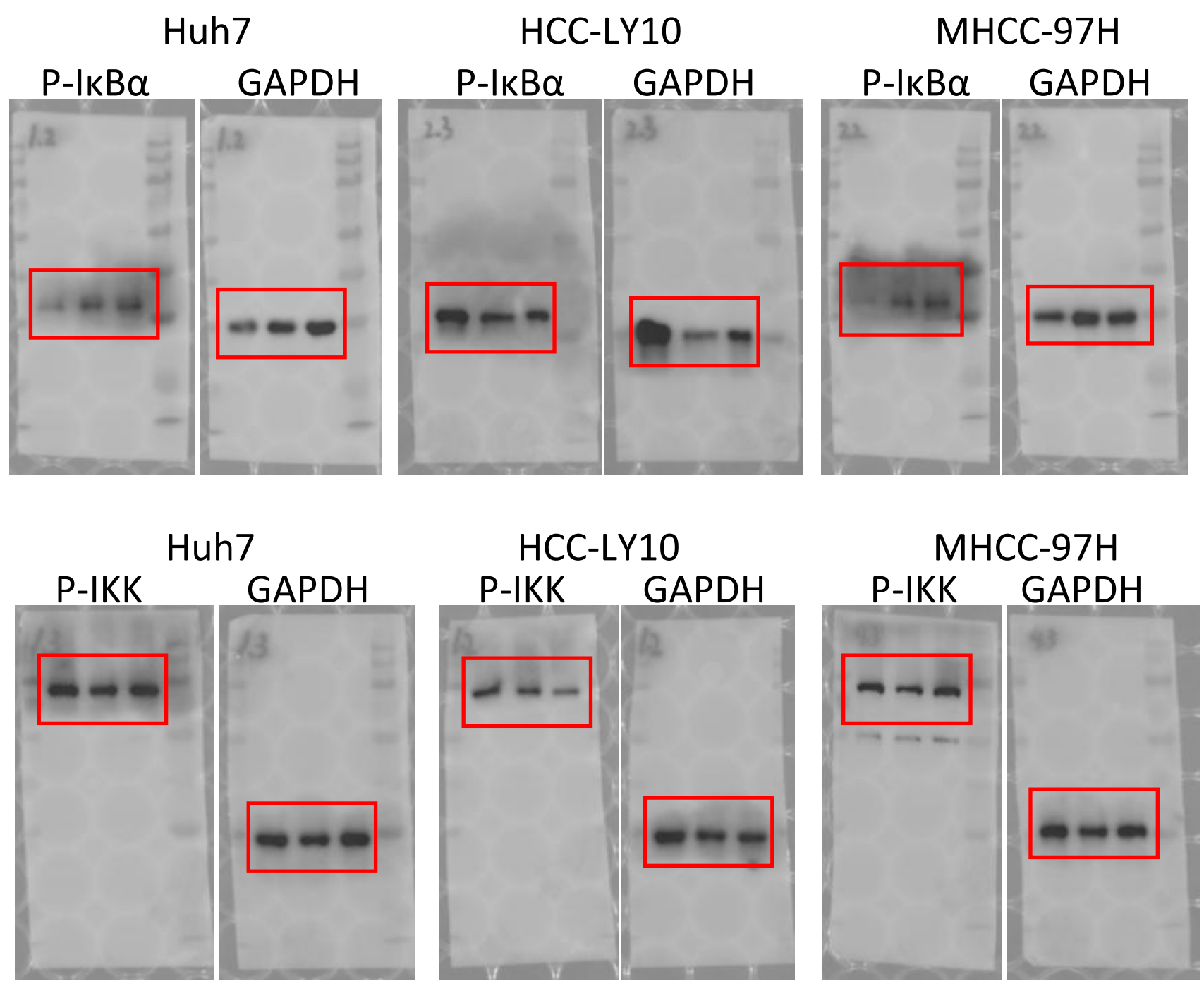

Fig.S17-36 Original blots corresponding to Fig.S9C

Fig.S17-37

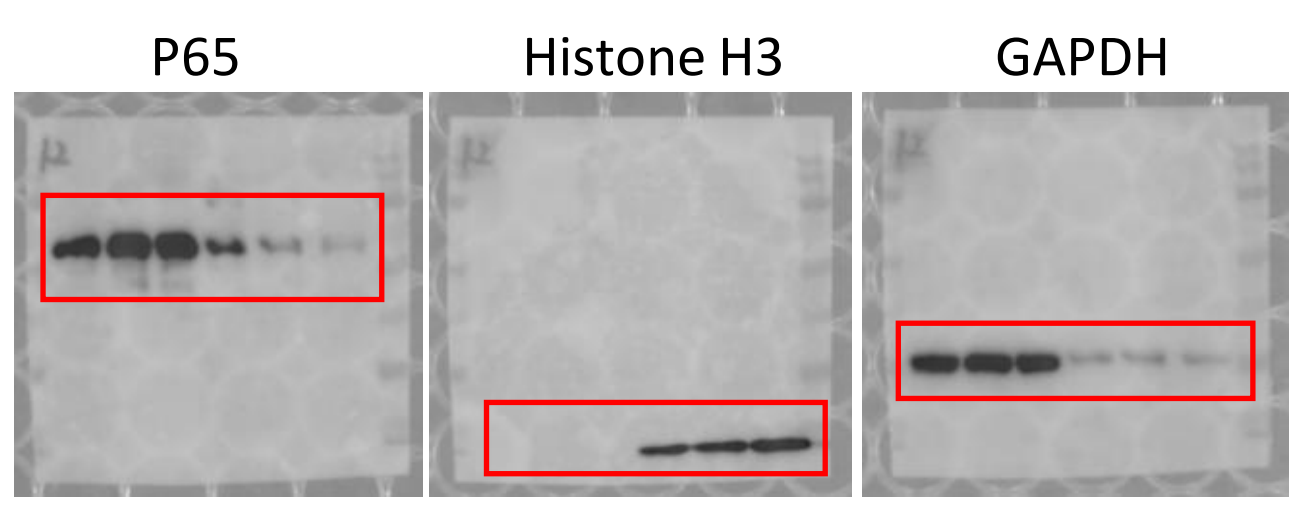

Fig.S17-37 Original blots corresponding to Fig.S9D

Fig.S17-38

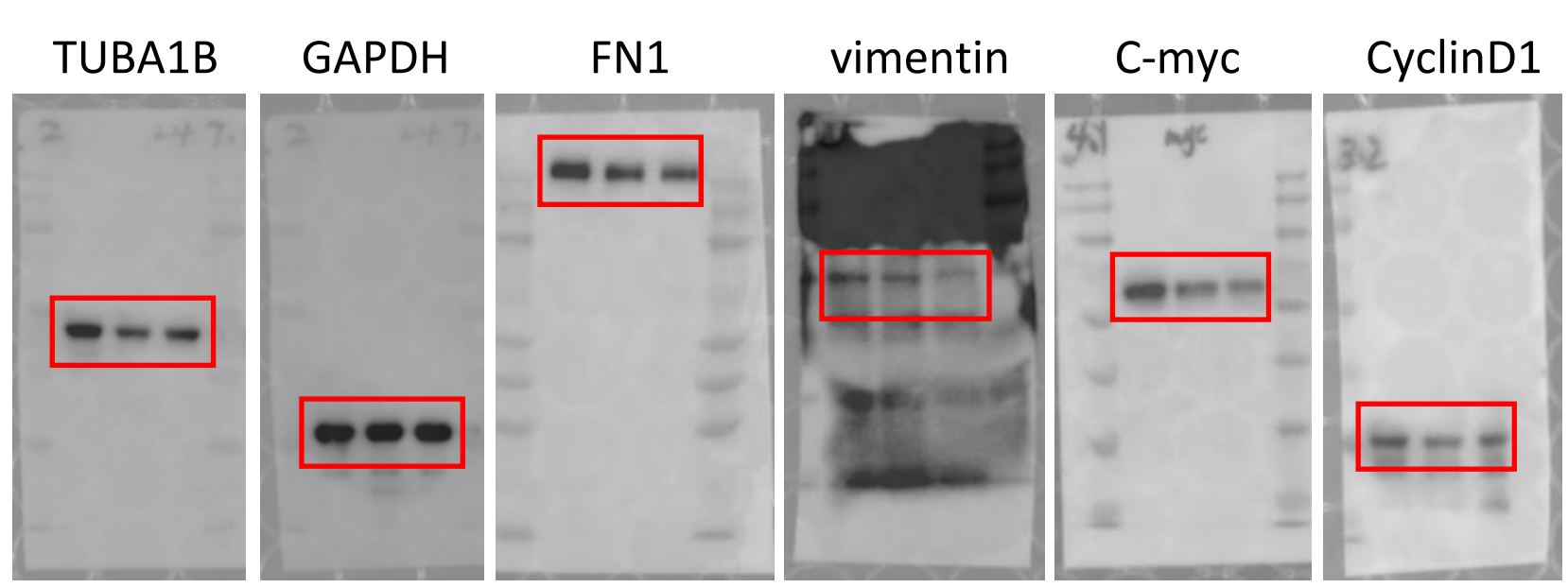

Fig.S17-38 Original blots corresponding to Fig.S9G

Fig.S17-39

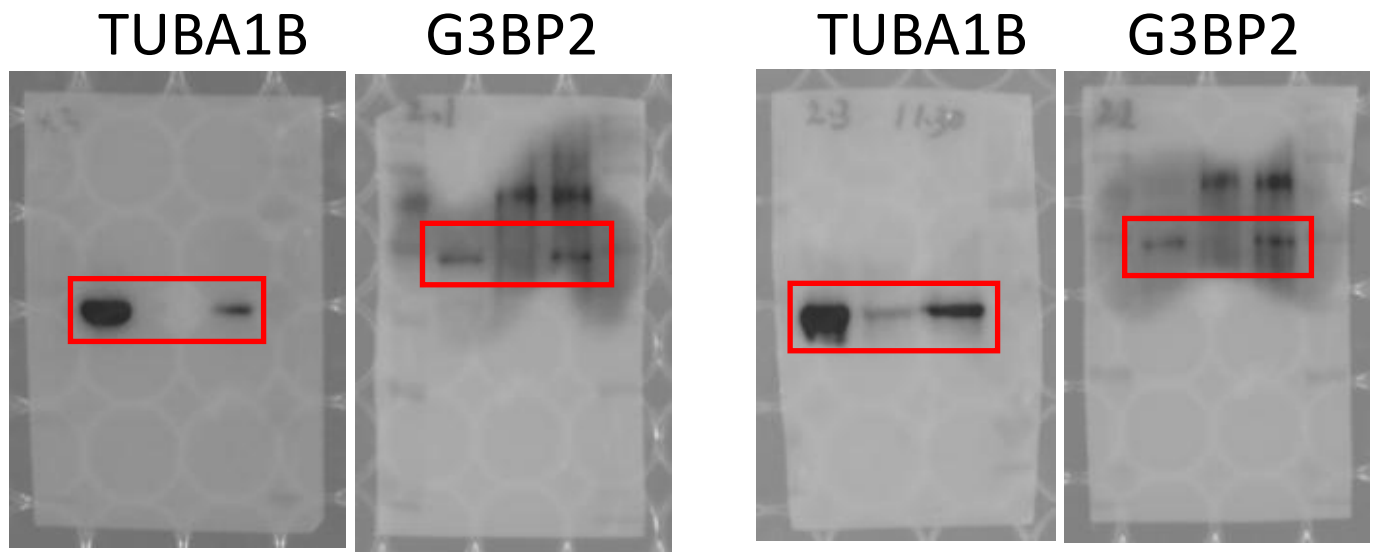

Fig.S17-39 Original blots corresponding to Fig.S10D

Fig.S17-40

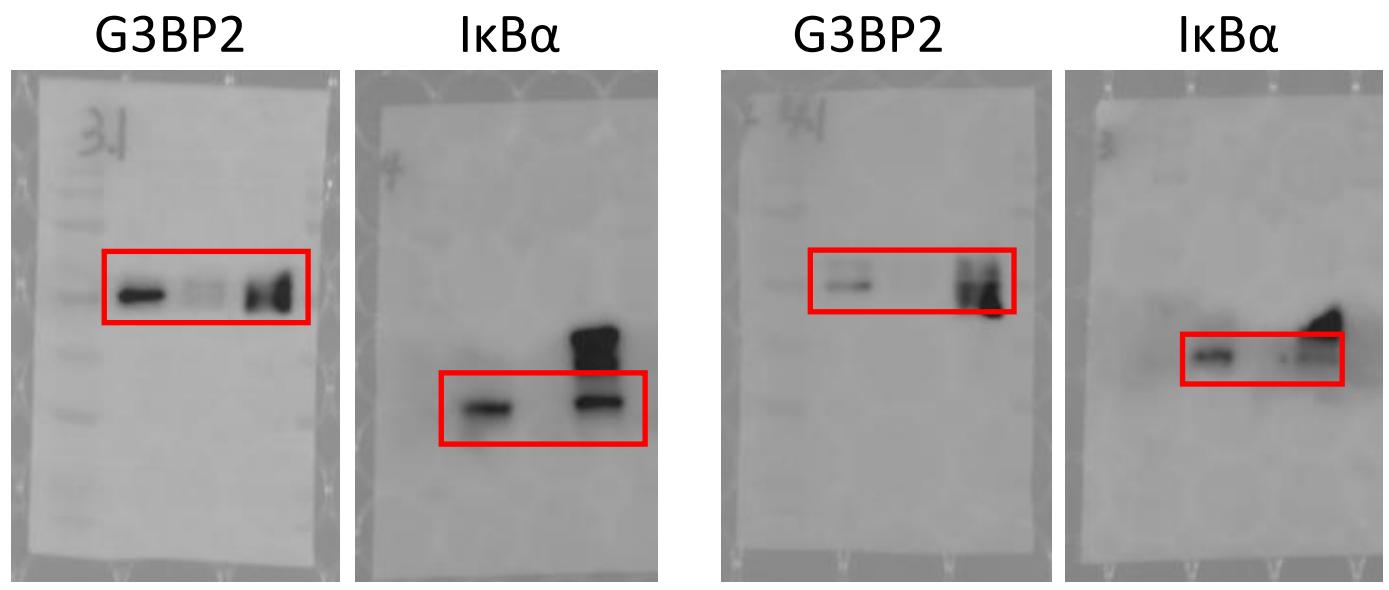

Fig.S17-40 Original blots corresponding to Fig.S10F

Fig.S17-41

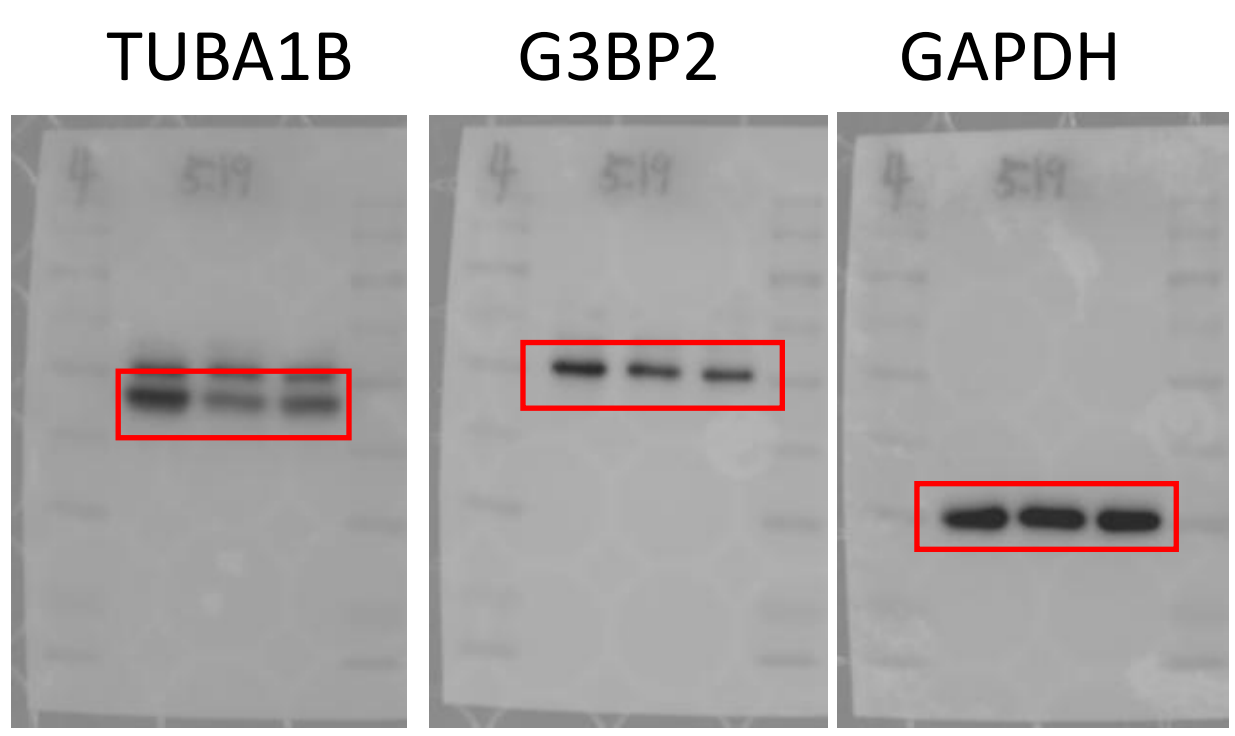

Fig.S17-41 Original blots corresponding to Fig.S10I

Fig.S17-42

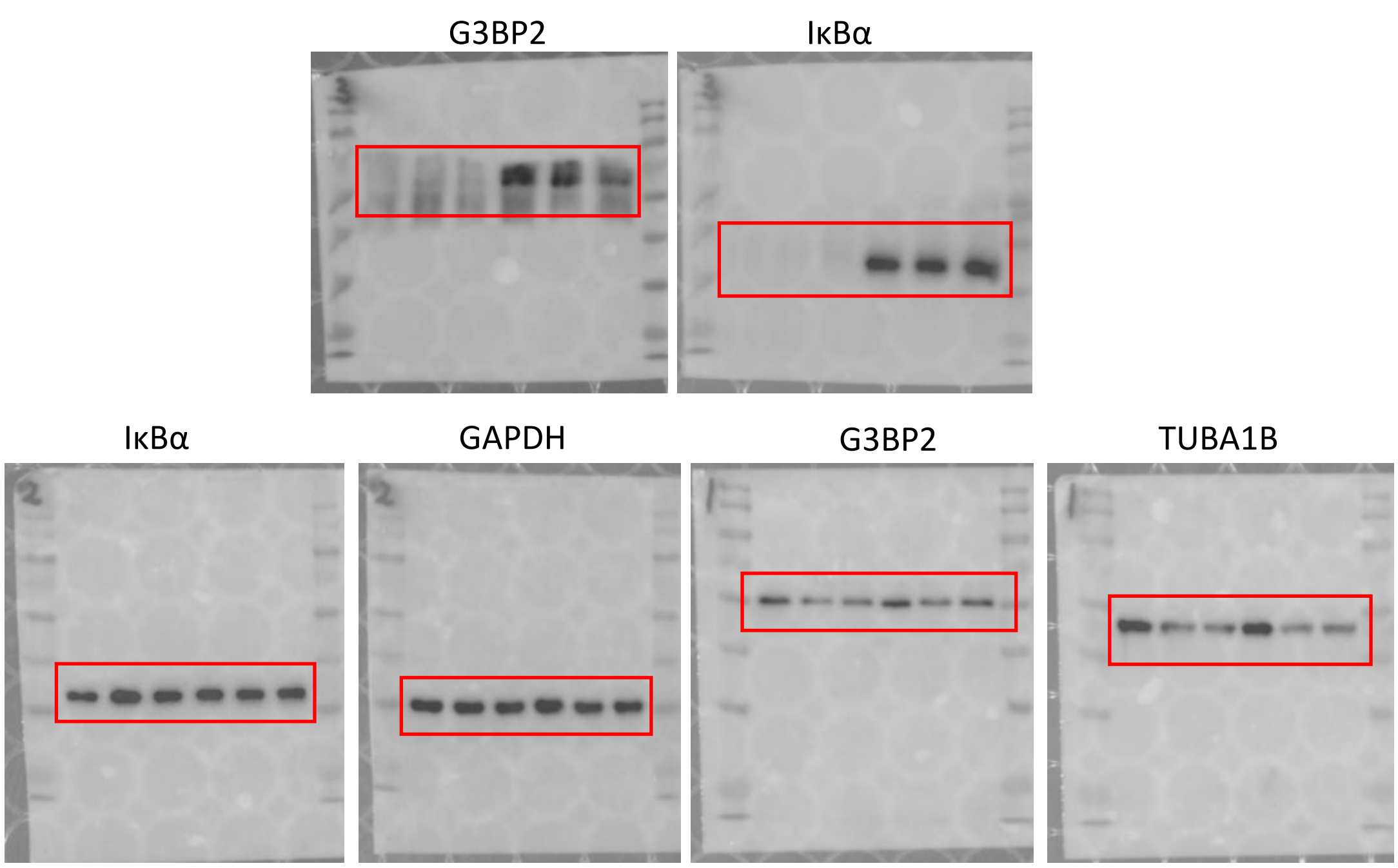

Fig.S17-42 Original blots corresponding to Fig.S10J

Fig.S17-43

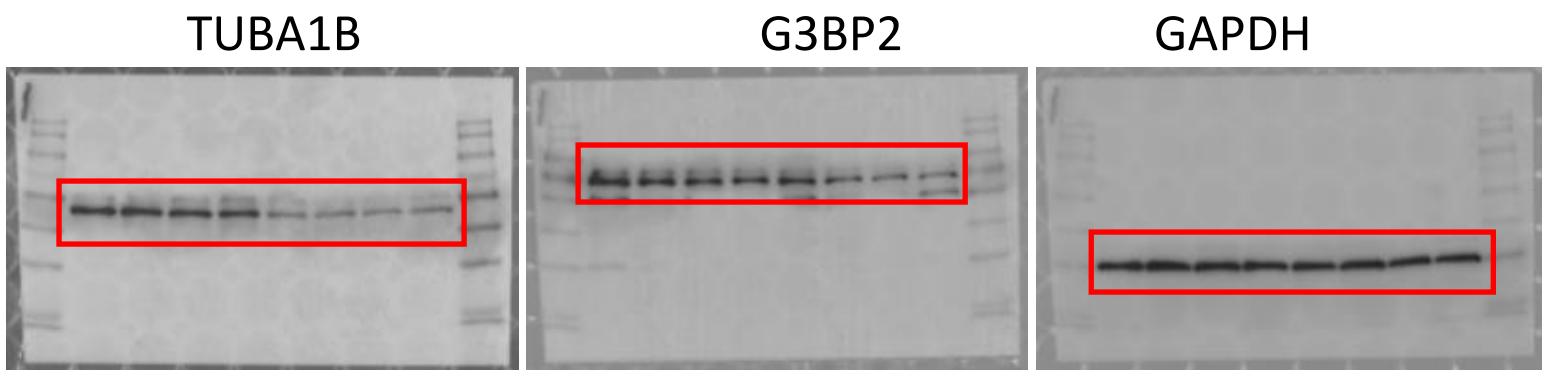

Fig.S17-43 Original blots corresponding to Fig.S11A

Fig.S17-44

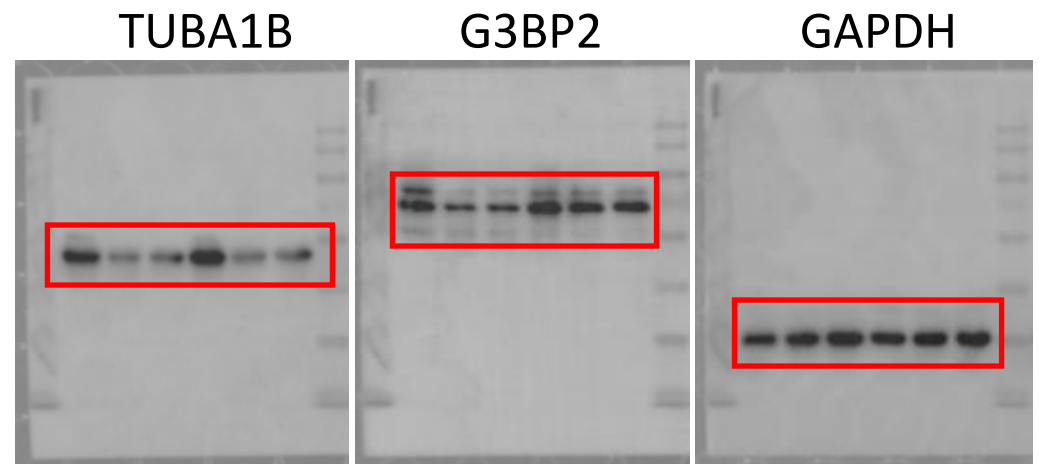

Fig.S17-44 Original blots corresponding to Fig.S11B

Fig.S17-45

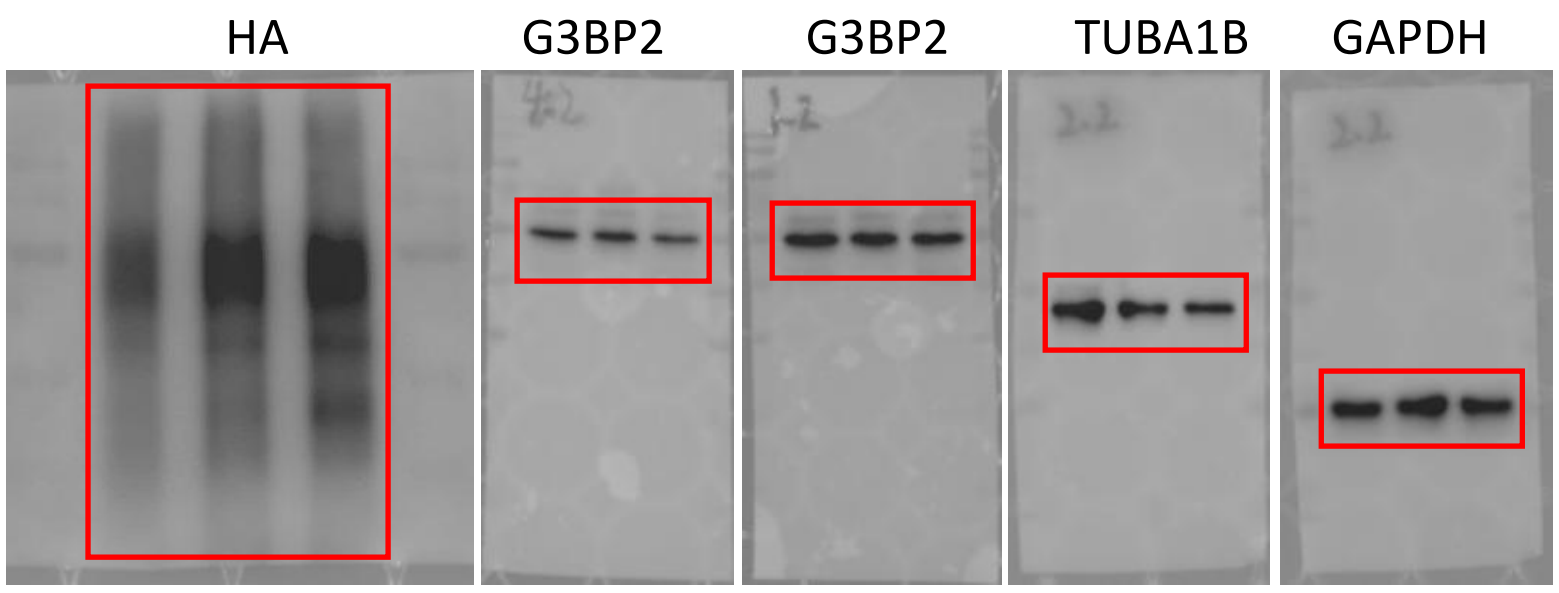

Fig.S17-45 Original blots corresponding to Fig.S11C

Fig.S17-46

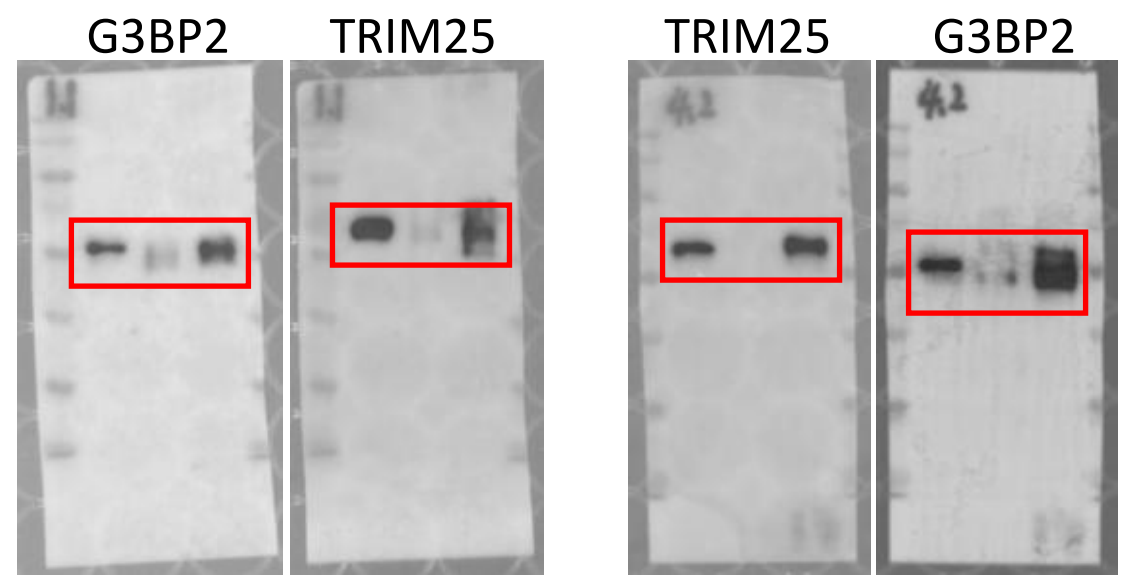

Fig.S17-46 Original blots corresponding to Fig.S11D

Fig.S17-47

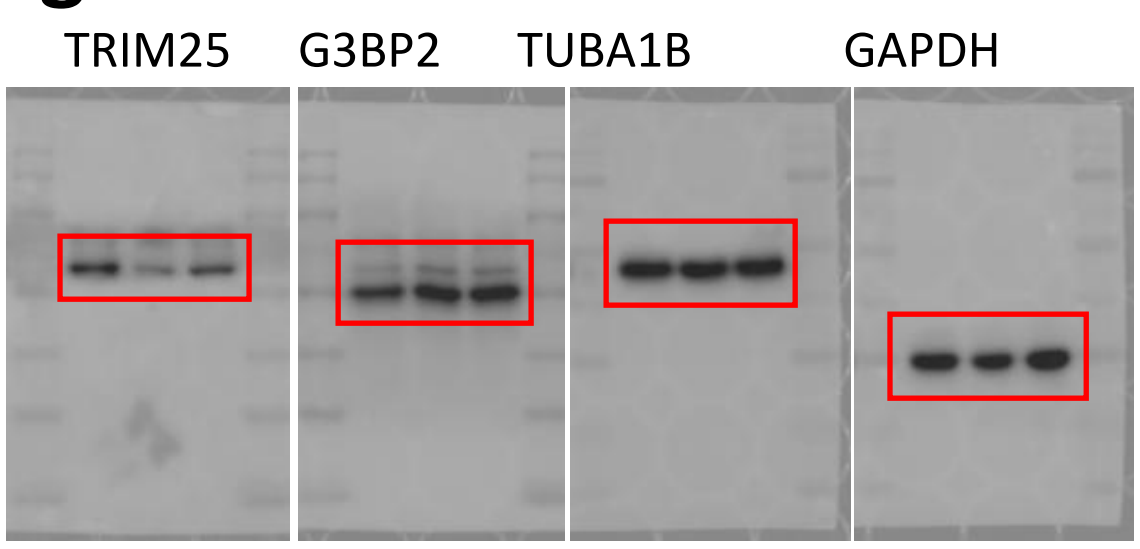

Fig.S17-47 Original blots corresponding to Fig.S11E

Fig.S17-48

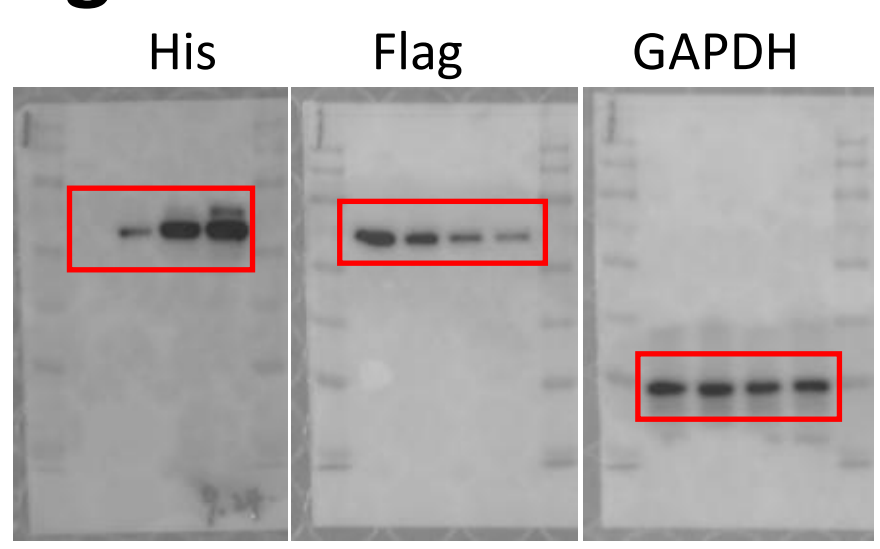

Fig.S17-48 Original blots corresponding to Fig.S11F

Fig.S17-49

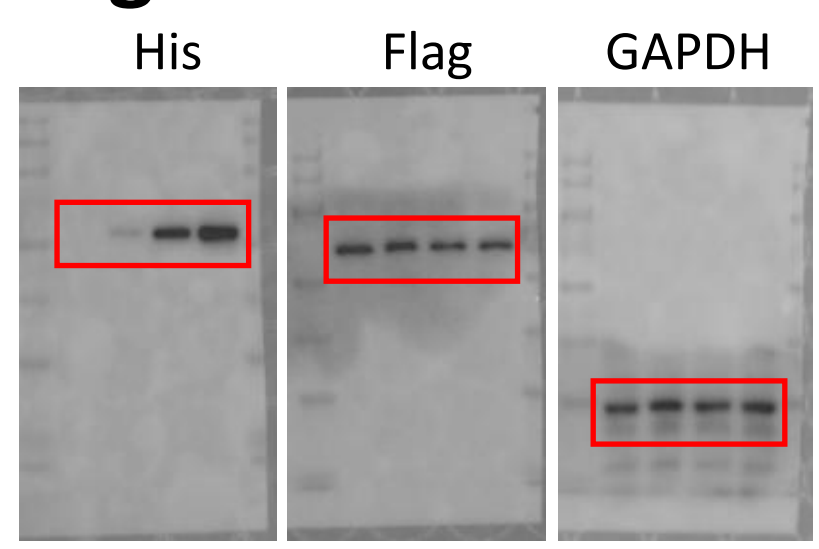

Fig.S17-49 Original blots corresponding to Fig.S11G

Fig.S17-50

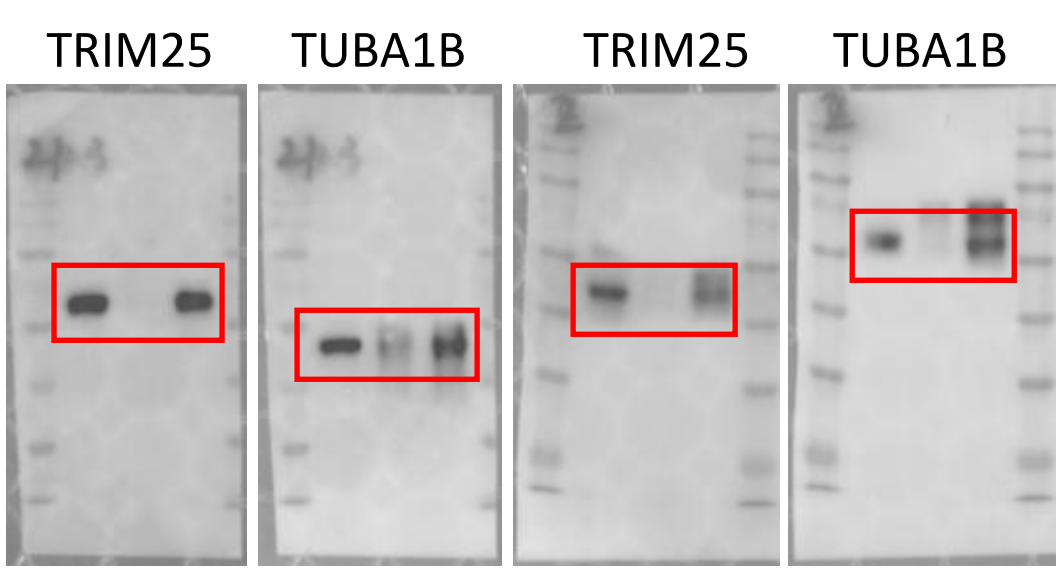

Fig.S17-50 Original blots corresponding to Fig.S11H

Fig.S17-51

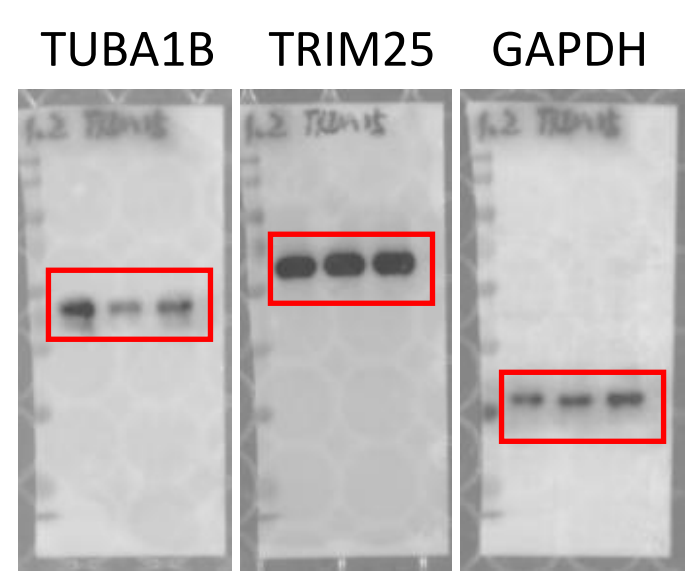

Fig.S17-51 Original blots corresponding to Fig.S11I

Fig.S17-52

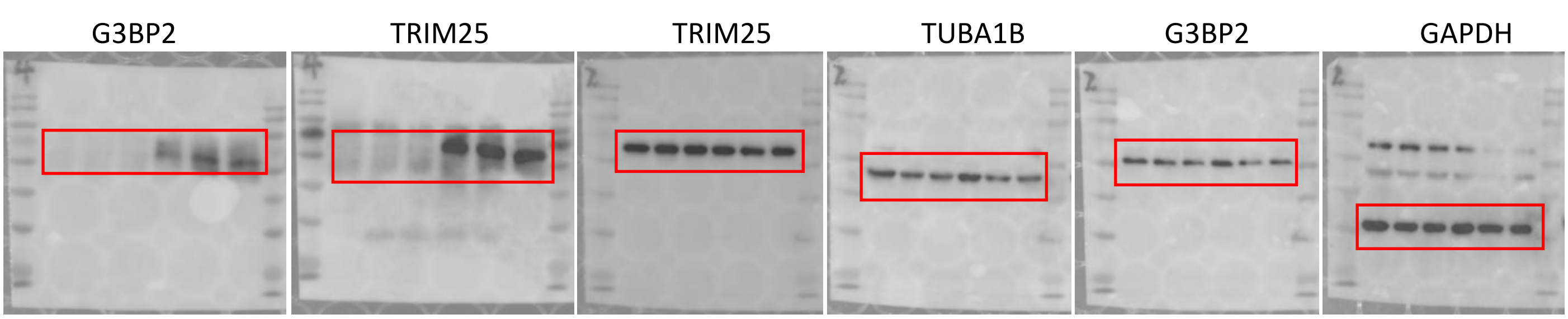

Fig.S17-52 Original blots corresponding to Fig.S11J

Fig.S17-53

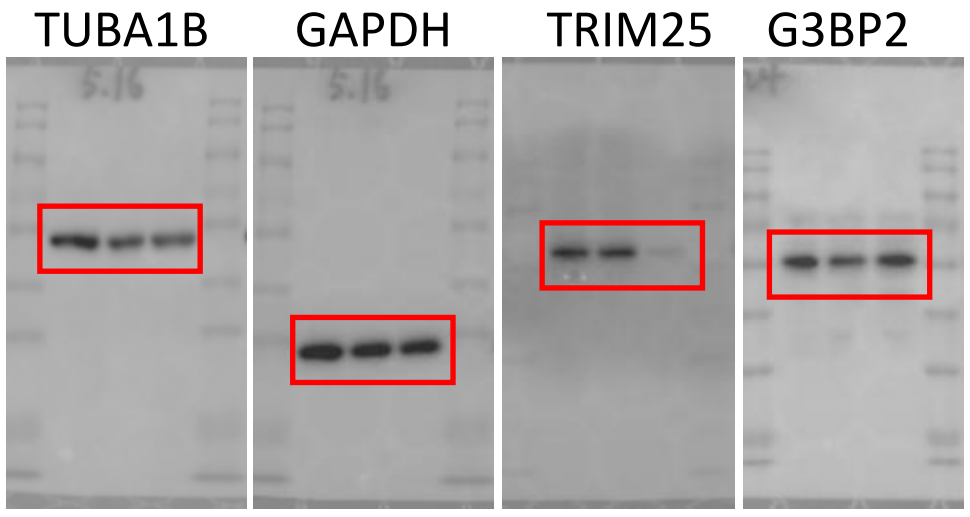

Fig.S17-53 Original blots corresponding to Fig.S11K

Fig.S17-54

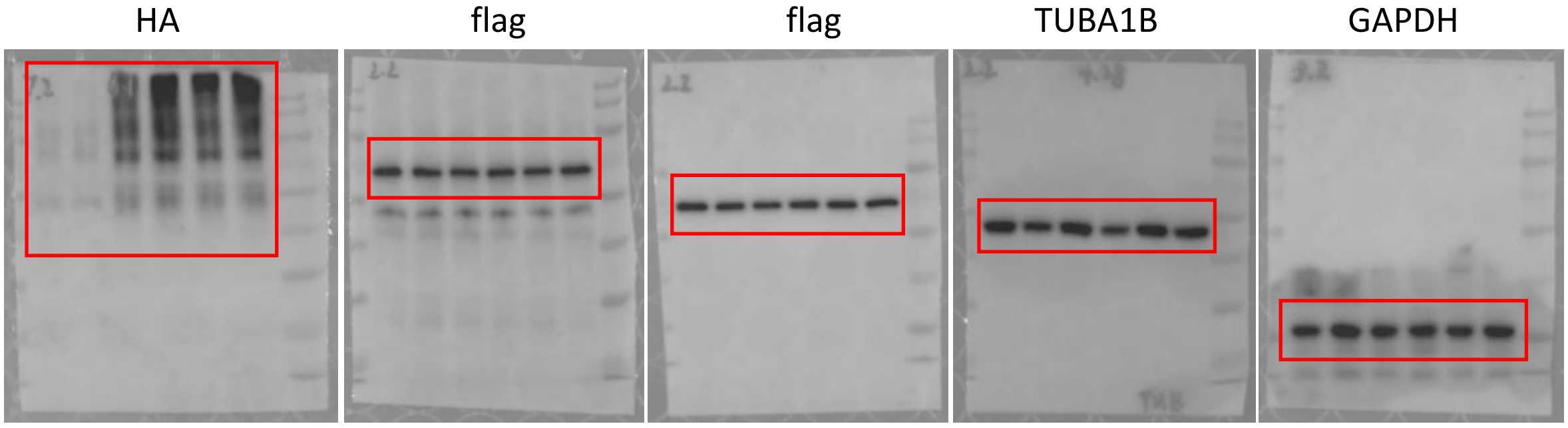

Fig.S17-54 Original blots corresponding to Fig.S11L

Fig.S17-55

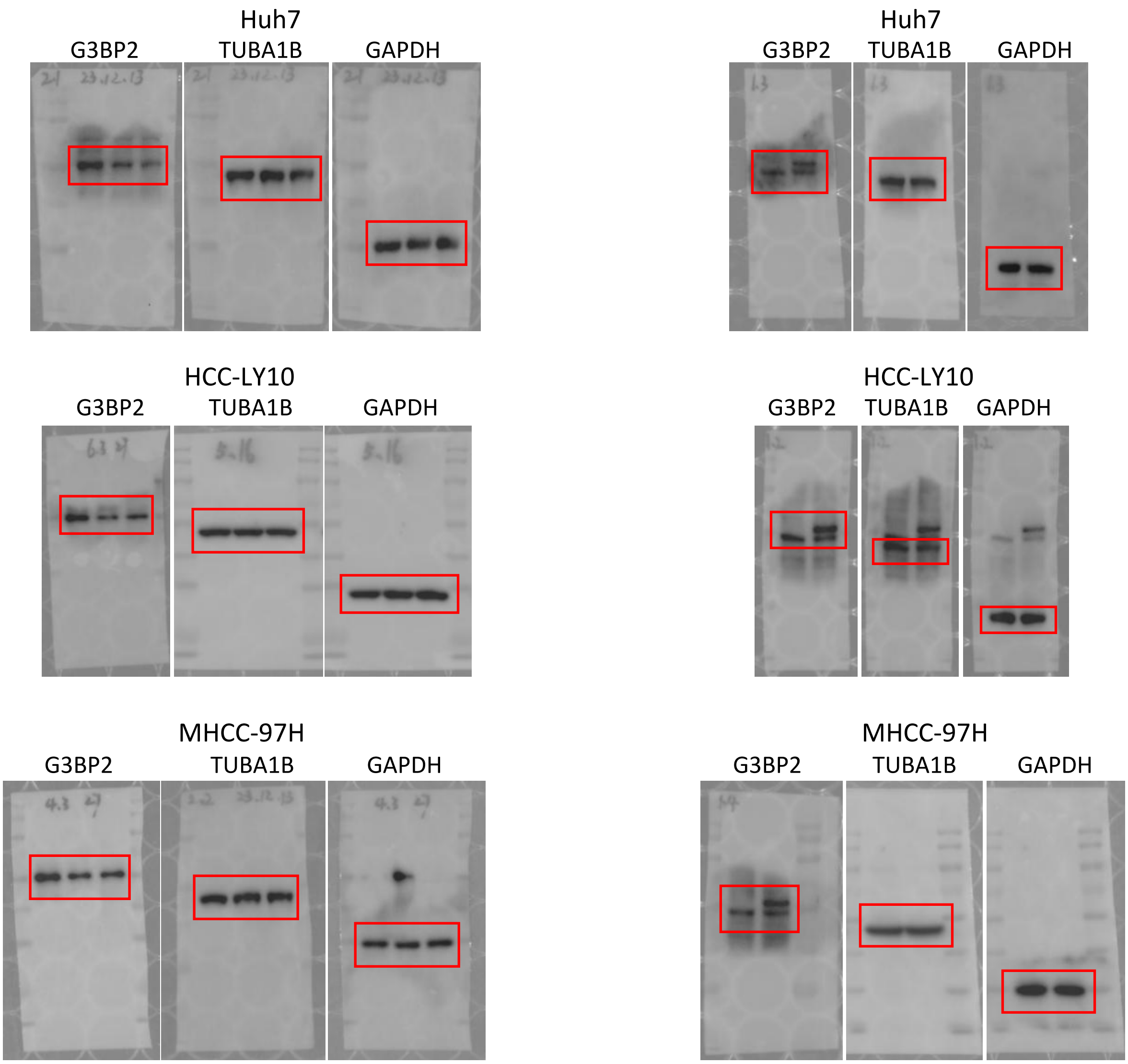

Fig.S17-55 Original blots corresponding to Fig.S12A

Fig.S17-56

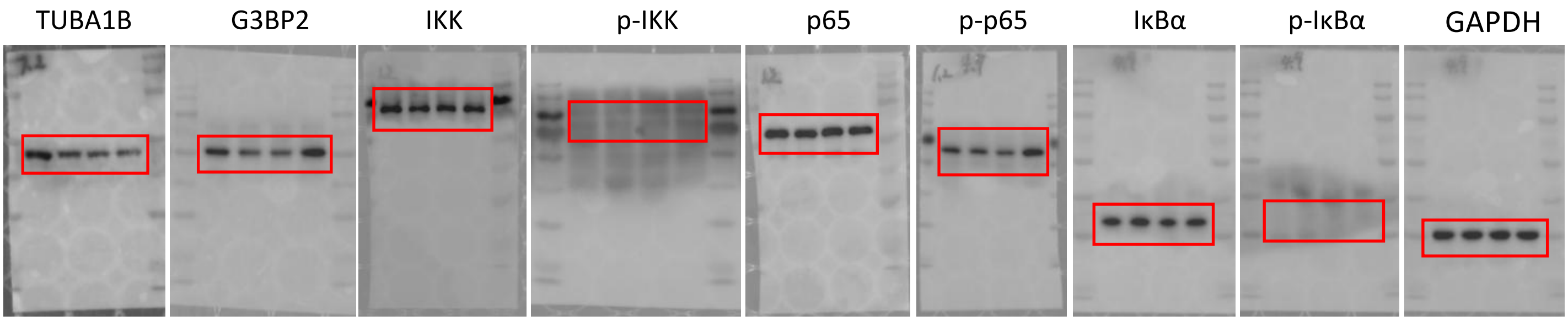

Fig.S17-56 Original blots corresponding to Fig.S13F

Fig.S17-57

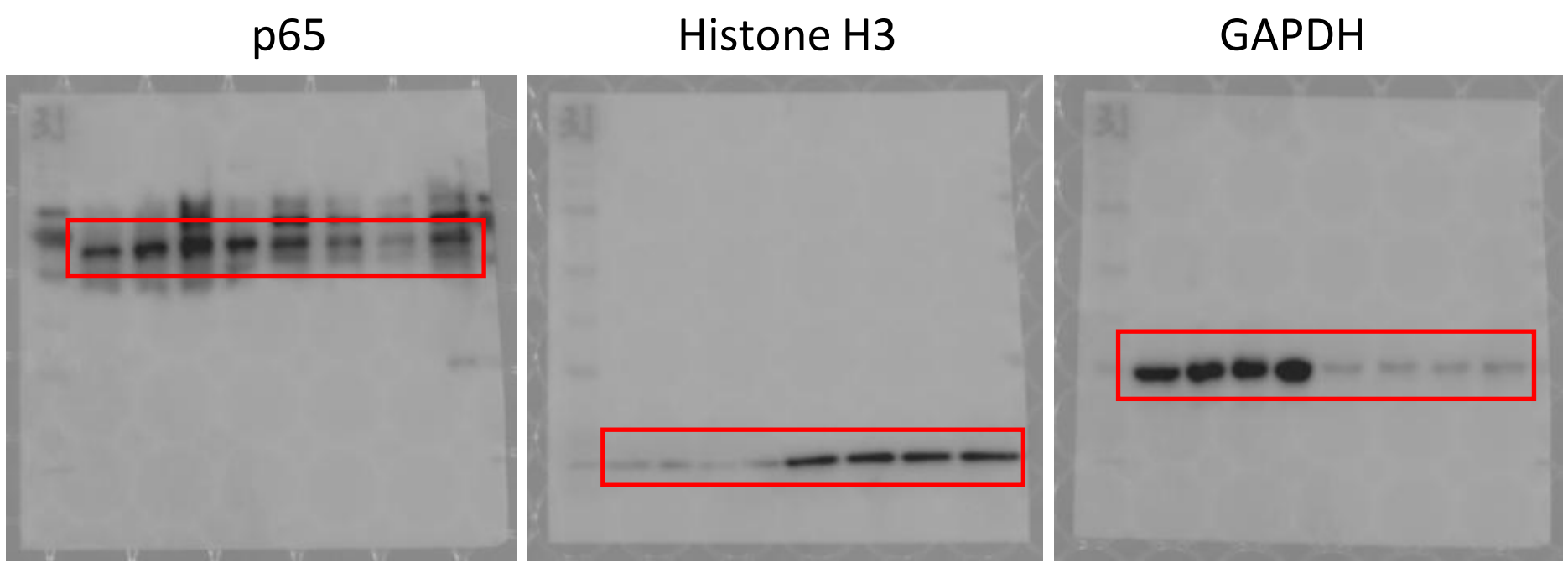

Fig.S17-57 Original blots corresponding to Fig.S13G

Fig.S17-58

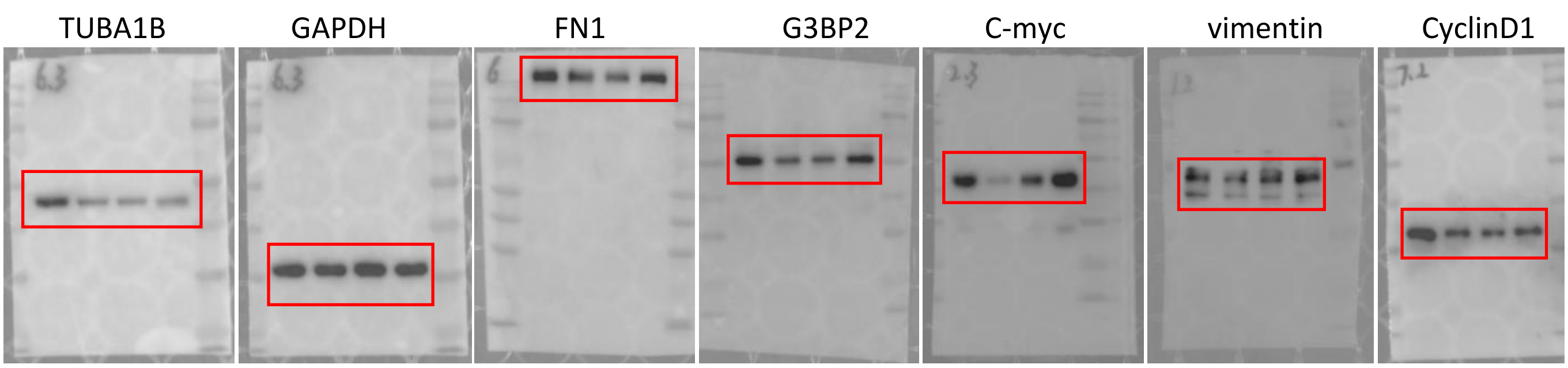

Fig.S17-58 Original blots corresponding to Fig.S13I
