# Supplementary material for: The TUBA1B–G3BP2 Axis Sustains p65 Transcriptional Activity to Promote Hepatocellular Carcinoma Progression
Source: Adv Sci (Weinh). 2026 Sep 6:e77502. Online ahead of print. doi: 10.1002/advs.77502 (PMC13547788; doi:10.1002/advs.77502)
Supplement: Supplementary file 2 [file ADVS-9999-e77502-s001.docx]

**Supplementary Information**

**Supplementary Methods**

**Clinical samples**

Human primary hepatocellular carcinoma (HCC) tissues (T) and paired adjacent non-cancer liver tissues (N) were obtained from patients at Qi Dong Liver Cancer Institute (Qi Dong, China). For western blot analysis, 30 paired fresh HCC tumor tissues and matched adjacent non-cancerous liver tissues were analyzed. For IHC analysis, HCC specimens from 199 patients were analyzed, and the baseline clinicopathological characteristics of this cohort are summarized in Table S7. All patients provided written informed consent prior to tissue collection, and no patient received preoperative radiotherapy or chemotherapy. This study was approved by the Research Ethics Committee of Renji Hospital, Shanghai Jiao Tong University School of Medicine and conducted in accordance with the Declaration of Helsinki.

**Plasmids and transfection**

The pLKO.1-shRNA-copGFP, pLKO.1-shTUBA1B-copGFP, pLKO.1-shTRIM25-copGFP, pcDNA3.1 vector, pcDNA3.1(+)/G3BP2-WT/N/M/C-3×FLAG, pcDNA3.1(+)/TUBA1B-WT/N/C-3×HA, pcDNA3.1(+)/TRIM25-6×His, pcDNA3.1(+)/TRIM25-ΔRING-6×His, lentiCRISPR-sgTUBA1B-copGFP, PX330-vector, PX330-sgTuba1b, pNF-kB-Luc , pRL-TK plasmids, pT3-EF1a vector, pT3-EF1a-Tuba1b, pT3-EF1a-Tubb2b and pT3-EF1a-Tubb were supplied by IGE Biotechnology (Guangzhou, China). The pLKO.1-shRNA control vector, pLKO.1-shG3BP2, pLVX-Vector and pLVX-G3BP2 plasmids were provided by the Renji Hospital Plasmid Repository (Shanghai, China). The plvx-TUBA1B-3×FLAG plasmid was purchased from iGeneBio (Guangzhou, China). The target sequences are listed in Table S3. The psPAX2, pMD2.G, pCW-Cas9, pRK5-HA-Ub-K48, pRK5-HA-Ub-K63, pRK5-HA-Ub, pT-CAG-NRasV12, pT3-myr-AKT and pCMV (CAT)T7-SB100 plasmids were gifts from Addgene listed in Table S4. Replication-deficient lentiviruses were produced using a second-generation packaging system. Lentiviral transfer vectors were co-transfected with helper plasmids (psPAX2 and pMD2.G) into 80-90% confluent HEK293FT cells using Lipofectamine® 2000 (Invitrogen, USA). Optimized plasmid ratios (transfer vector: psPAX2: pMD2.G = 4:3:1) and DNA/Lipofectamine 2000 ratio (1:2) were used. Viral supernatants were harvested at 48 h post-transfection, clarified by centrifugation at 1,000 × g for 3 min, and filtered through 0.22 μm filters (RS-PES-22, Biosharp). HCC cells were infected with lentiviruses at a 1:10 dilution, in the presence of 6 μg/mL polybrene (40804ES76, Yeasen). After 24 h of incubation, the medium was replaced with a fresh complete medium. Transduction efficiency was confirmed by >80% GFP⁺ cells via fluorescence microscopy (Zeiss, Germany) at 72 h post-infection.

**Immunohistochemical (IHC) analysis**

Following xylene deparaffinization and graded ethanol rehydration, sections underwent antigen retrieval via microwave heating at 95-98°C in Tris/EDTA buffer (pH 9.0) for 20 minutes with subsequent 20-minute natural cooling. Endogenous peroxidase blocking employed 3% H₂O₂/methanol followed by 30-minute non-specific blocking with 5% normal goat serum. Sections were incubated overnight at 4°C with primary antibodies listed in Table S2, PBS-washed, then treated with HRP-conjugated species-matched secondary antibodies for 1 hour at room temperature prior to DAB chromogenic development with hematoxylin counterstaining. Two blinded pathologists independently scored staining intensity (0: negative; 1: weak; 2: moderate; 3: strong) and positive cell percentage (0: 0%; 1: < 10%; 2: 10-35%; 3: 35-75%; 4: > 75%), calculating immunoreactive scores through score multiplication (IRS range: 0-12). Specimens with IRS ≥ 3 were classified high-expression and IRS < 3 low-expression, with discrepant evaluations resolved via joint microscopy re-examination. IHC quantification was performed in FIJI with IHC Profiler on 20× whole-slide scans. Vimentin and FN1 were quantified as Positive area (%), and total p65, p-p65 (S536), c-myc and Cyclin D1 by Combined H-score (0–300) based on nuclear intensity categories (0–3). Ki67 and PCNA were assessed on 80× ROIs as nuclear positivity (% positive nuclei). Complete antibody specifications including dilutions are archived in Table S2.

**Real-time quantitative PCR**

Cells were harvested from 6-well culture plates and total RNA was extracted using TRIzol reagent (15596018, Invitrogen). Then, cDNA was generated using PrimeScriptTM RT Reagent Kit (RR037A, Takara) and real-time quantitative PCR was performed using TB Green® Premix Ex TaqTM II (RR820A, Takara) on an Applied Biosystems 7500 (Thermo Fisher Scientific, MA, USA). The threshold cycle value of the target gene was normalized to that of GAPDH, and the relative expression of the target genes was calculated using the 2−ΔΔCt method. The primer sequences are listed in Table S5.

**RNA-sequencing**

Huh7 and MHCC-97H cells were transfected with shTUBA1B or shNC for 48 hours, after which total RNA was extracted using TRIzol reagent. RNA samples underwent sequencing by Shanghai Sinotech genomics Co., Ltd. (Shanghai, China). Subsequently, differential expression analysis was performed using DESeq2, with genes meeting adjusted *p* < 0.05 classified as differentially expressed. Finally, Gene Ontology (GO) and Kyoto Encyclopedia of Genes and Genomes (KEGG) enrichment analyses of these differentially expressed genes were conducted via clusterProfiler, applying a significance threshold of FDR-adjusted *p* < 0.05.

**Western Blotting**

Total cellular proteins were extracted using RIPA lysis buffer (Millipore). Protein concentrations were quantified with a BCA Protein Assay Kit (P0010, Beyotime). Nuclear and cytoplasmic fractions were isolated using a NE-PER Nuclear and Cytoplasmic Extraction Reagent Kit (78833, Thermo Scientific) following manufacturer's specifications. Total protein (30 μg) was resolved by SDS–PAGE and electro-transferred to PVDF membranes (Millipore). Membranes were blocked with 5% nonfat dried milk in PBST for 1 h at room temperature, followed by incubation with primary antibodies overnight at 4°C. After PBST washes, the membrane was incubated with HRP-conjugated secondary antibody for 2 h at room temperature, followed by detection using ECL substrate (GE Healthcare). The images of protein bands were analyzed in a semi-quantitative manner through ImageJ software. The original uncropped scans of the blots were provided in the Source Data file. Antibody details are provided in Table S2.

**Cell growth assay and colony formation assay**

Cell proliferation assays were conducted by seeding cells in 96-well plates at a density of 1,000 cells per well. Following overnight incubation to ensure attachment, viable cells were quantified at 24-hour intervals using the Celigo Image Cytometer (Nexcelom, USA), which automatically scans and counts cells based on morphological criteria. For colony formation analysis, cells were plated in six-well plates at 1000 cells per well to ensure discrete colony development. Cultures were maintained for 14 days in a humidified incubator at 37°C with 5% CO₂, with medium refreshed every 3 days. Resultant colonies were fixed with 4% paraformaldehyde for 30 minutes at room temperature, rinsed twice with PBS, and stained for 15 minutes with 0.5% Giemsa solution (Thermo). Colonies were imaged under brightfield microscopy (Nikon Eclipse Ti) and manually counted using ImageJ's particle analysis module. All experiments were performed with three independent biological replicates.

**Wound healing assay**

HCC cells were plated in 6-well plates at 1×10⁶ cells/well. After 24-h incubation forming confluent monolayers, scratch wounds were created using pipette tips followed by two 1× PBS washes. Cells were maintained in 2 mL DMEM with 2% FBS and 1 mM thymidine (Sigma-Aldrich). Gap closure was monitored at 0, 12, 24, and 48 h using the Lionheart™ FX Automated Live Cell Imager (Agent BioTek). Migration rates were calculated as [(Area0h - Areath)/Area0h] × 100% using ImageJ-measured gap areas (excluding ±10% edge regions to avoid distortion). All experiments included triplicate biological replicates with three technical replicates each, conducted at 37°C/5% CO₂ in humidified chambers.

**Transwell assay**

Cell migration and invasion capacities were assessed using Transwell® chambers (8-μm pore; Corning #3422). For invasion assays, membranes were pre-coated with Matrigel (Corning #354234) at 37°C for 1 h prior to cell seeding. Cells suspended in serum-free DMEM (Huh7: 1 × 10⁵, HCC-LY10: 1.5 × 10⁵, MHCC-97H: 2 × 10⁵ cells/well) were seeded in the upper chamber, while the lower chamber contained 700 μL DMEM with 10% FBS as chemoattractant. After 24 h (Huh7) or 48 h (HCC-LY10, MHCC-97H) of incubation, the non-migrated or non-invaded cells were gently removed from the upper chamber using a cotton swab. The remaining cells were fixed and stained using crystal violet solution (Invitrogen, CA, USA) and counted in three randomly selected visual fields.

**Ultra-low attachment plate-based cell aggregation assay**

HCC cells in the logarithmic growth phase were digested using trypsin, pelleted by centrifugation, and resuspended. Subsequently, the cells were seeded into Corning 6-well ultra-low attachment plates at a density of 300,000 cells per well, with each well containing 2 mL of DMEM supplemented with 10% FBS. Cultures were maintained overnight at 37°C in a 5% CO₂ humidified incubator for approximately 16-18 hours. The following day, cellular aggregation patterns were evaluated using a Zeiss inverted microscope. Spatial architecture and marginal morphological features of cellular clusters were systematically documented, with images captured from a minimum of three independent visual fields for subsequent analysis.

**5-Ethynyl-2’-deoxyuridine (EdU) assay**

Cell proliferation was assessed using the BeyoClick™ EdU-594 Kit (C0078S, Beyotime) in 12-well plates. After overnight seeding, cells were washed with PBS and incubated with pre-warmed EdU solution (10 µM final concentration) for 2 hours. Following aspiration, cells were fixed with 4% paraformaldehyde (15 min, RT) and permeabilized with 0.3% Triton X-100 solution (15 min, RT). The Click reaction solution was freshly prepared by sequentially combining Click Reaction Buffer, CuSO₄, Azide 594, and Click Additive Solution according to the kit instructions. Subsequently, 500 µl of the freshly prepared Click reaction solution was added to each well, and the plate was incubated at room temperature for 30 minutes in the dark. Cell nuclei were then stained with 1× Hoechst 33342 solution and incubated at room temperature for 10 minutes in the dark, followed by three washes with PBS. Finally, samples were examined under a fluorescence microscope.

**Mass spectrometry (MS) analysis**

Total proteins were extracted from MHCC-97H and Huh7 cells using lysis buffer followed by immunoprecipitation with an anti-TUBA1B antibody (11224-1-AP, Proteintech) or control IgG. The immunoprecipitated samples alongside input controls underwent SDS-PAGE separation. Gels were stained with Coomassie Blue solution (P0017, Beyotime) under constant agitation at 37°C for 20 h, after which target bands were excised and destained in a methanol: glacial acetic acid: ultrapure water solution (100:100:800, v/v/v) overnight at room temperature with agitation. Destained gel bands were submitted to APTBIO (Shanghai, China) for mass spectrometry analysis (Project ID: YAS202309070053-1). Protein entries or protein groups showing a TUBA1B-IP/IgG enrichment ratio greater than 1, or detected exclusively in the TUBA1B-IP samples but not in the corresponding IgG controls, were retained for initial candidate identification.

**Co-immunoprecipitation**

Cell lysates were prepared using lysis buffer (Beyotime, cat# P0013). Aliquots containing 1 mg of total protein were incubated with 1 μg of the indicated antibody overnight at 4°C. Pre-treated Protein A/G magnetic beads (B23202, Selleck) were added to the antigen-antibody complexes and incubated for 6 h at 4°C. The beads were subsequently washed three times with lysis buffer. For selected IP-WB experiments requiring improved background control, the IP antibody was crosslinked to Protein A/G magnetic beads before incubation with cell lysates to reduce antibody carryover during elution. An IP-compatible secondary antibody was used for immunoblot detection to minimize IgG-associated detection background. Finally, the beads were eluted with SDS-PAGE loading buffer, and the resultant eluates were subjected to immunoblotting analysis.

**Immunofluorescence confocal imaging**

Cells were grown on Lab-Tek chamber slides (Nunc), fixed with 4% paraformaldehyde for 30 min and permeabilized with 0.2% Triton X-100 for 5 min. After that, cells were blocked with 3% BSA (FA106-100G, GENEVIEW) at room temperature for 1 hour, incubated with primary antibodies (Table S2) overnight at 4℃ in a humidified chamber. Subsequently, the glass slides were washed three times in PBS and incubated with Alexa Fluor 594-conjugated or Alexa Fluor 488-conjugated secondary antibodies (Table S2) 2 h at room temperature, followed by sealing with Antifade Mounting Medium with DAPI (Beyotime, P0131). To detect the actin cytoskeleton, cultured HCC cells were gently rinsed twice with ice-cold PBS, followed by fixation with 4% methanol-free paraformaldehyde for 20 minutes at room temperature. After permeabilization using 0.1% Triton X-100 in PBS (three 5-minute washes), cells were incubated with 200 μL of Actin-Tracker Red-594 (Beyotime, C2205S; 1:100 dilution in PBS containing 3% BSA and 0.1% Triton X-100) under light-protected conditions at room temperature for 60 minutes. Following three additional 5-minute washes with 0.1% Triton X-100/PBS, samples were mounted using DAPI-containing antifade mounting medium (Beyotime, P0131) for nuclear counterstaining. Imaging was acquired using a confocal microscope (Leica, Germany). Cells containing at least three clearly visible linear F-actin bundles across the cell body were classified as stress fiber-positive.

**Molecular docking analysis**

Rigid protein–protein docking was performed between TUBA1B and G3BP2 to investigate the relationships by using GRAMM-X (http://gramm.compbio.ku.edu/). The protein structural domains of TUBA1B (PDB: 7sja C) and G3BP2 (PDB: 5drv A) were obtained from the Protein Data Bank PDB database (http://www.rcsb.org/). The docking simulation generated a low-energy complex model (binding energy: -9 kcal/mol), indicating stable complex formation. Pymol (Version 2.4) and PDBePISA (https://www.ebi.ac.uk/pdbe/pisa/) were used to investigate protein-protein interactions and visualize key binding domains at the interface.

**Protein half-life detection and ubiquitination assay**

Cells in the logarithmic growth phase were treated with 20 μM cycloheximide (CHX, Thermo, 239765) for 0, 5, 10, or 15 hours. Protein levels were analyzed by Western blot, and half-lives were calculated from densitometric measurements. For ubiquitination assays, HCC cells were transduced with shTUBA1B or non-targeting shRNA for 24 hours, followed by medium replacement. Cells were then co-transfected with Ub-HA using jetPRIME® (Polyplus, 101000046) for 24 hours and treated with 10 μM MG132 (MCE, USA) for 20 hours. Ubiquitinated proteins were analyzed by co-immunoprecipitation (co-IP). All experiments were independently repeated at least three times.

**Dual-luciferase reporter assay**

HCC cells were first infected with lentiviral carrying shTUBA1B or control shRNA for 24 hours, followed by complete medium replacement. Then, cells were co-transfected with the pNF-κB-Luc firefly reporter plasmid and pRL-TK Renilla control plasmid at a 5:1 ratio using jetPRIME® (101000046, Polyplus). Cell lysates were prepared 24 hours post-transfection using Passive Lysis Buffer and analyzed with Promega's Dual-Luciferase Reporter Assay System (Promega, Cat# E1960). Firefly luminescence values were normalized to Renilla readings (triplicate measurements) to determine relative NF-κB activity. The reduced normalized ratio reflected suppressed p65 transcriptional activity under TUBA1B- knockdown.

**Statistical analysis**

Quantitative data are presented as mean ± SD unless otherwise indicated. Statistical analyses and graph generation were performed using GraphPad Prism 8.4.3. Two-group comparisons were performed using two-sided Student’s t-tests, as appropriate. Comparisons among three or more groups were performed using one-way ANOVA followed by Dunnett’s or Šídák’s multiple-comparisons test, as appropriate. Two-factor experiments were analyzed using two-way ANOVA followed by Šídák’s multiple-comparisons test. For Celigo proliferation assays, statistical comparisons were performed at the final time point. The log-rank test was used to compare Kaplan–Meier survival curves. Categorical variables were analyzed using the chi-square test or Fisher’s exact test, and correlations were assessed using Pearson’s or Spearman’s correlation analysis, as indicated. Statistical significance is denoted as follows: *P < 0.05, **P < 0.01, ***P < 0.001, and ****P < 0.0001. Bioinformatics analyses were performed using R version 4.5.1 and Gene Set Enrichment Analysis software version 4.3.

**Supplementary Figures and Legends**

**Figure S1. Comparative analysis of microtubule protein upregulation across diverse HCC models. (A)** Using the GSE148379 dataset, we examined the upregulation of various microtubule protein isoforms (*Tuba1a, Tuba1b, Tuba1c, Tuba3a, Tuba3b, Tuba4a, Tuba8, Tubb1, Tubb2a, Tubb2b, Tubb3, Tubb4a, Tubb4b, Tubb5,* and *Tubb6*) across 11 genetically defined hepatocellular carcinoma (HCC) models, including *MYC;sg-p53, MYC;sg-Kmt2b, MYC;sg-Kmt2c, MYC;sg-Pten, MYC;CTNNB1, MYC;sg-Axin1, CTNNB1;sg-Pten, CTNNB1;Tert, MYC;Tert, myc-luc-bcat,* and *myc-lucOS-bcat*. All models were established via hydrodynamic tail vein injection, combining a known HCC driver (MYC or β-catenin) with additional genetic perturbations to generate specific oncogenic contexts.

 **Figure S2. TUBA1B is consistently upregulated among tubulin isoforms across multiple HCC cohorts. (A)** Differential expression of various tubulin isoforms (TUBA1A, TUBA1B, TUBA1C, TUBA3C, TUBA3D, TUBA3E, TUBA4A, TUBA8, TUBB, TUBB1, TUBB2A, TUBB2B, TUBB3, TUBB4A, TUBB4B, TUBB6, and TUBB8) in the TCGA-LIHC cohort. **(B)** Expression profiles of tubulin isoforms in the LIRI-JP cohort. **(C)** Expression profiles of tubulin isoforms in the CHCC-HBV cohort. **(D)** TUBA1B mRNA expression in HCC tumor tissues versus adjacent non-tumor liver tissues across public cohorts, including TCGA-LIHC, CHCC-HBV, LIRI-JP, and six independent GEO datasets: GSE124535, GSE144269, GSE69164, GSE77314, GSE77509, and GSE94660.

**Figure S3.** **TUBA1B expression and clinical relevance in HCC.** (A) Kaplan-Meier overall survival curves for TUBA1B in HCC-HBV and LIRI-JP cohorts. (B) GSE14520 analysis showing TUBA1B overexpression correlates with higher metastatic risk. (C, D) qRT-PCR (C) and Western blot (D) analysis of TUBA1B in HCC cell lines. Original blots can be found in Fig. S17-28.

**Figure S4.** **Establishment and validation of shRNA-mediated and doxycycline-inducible TUBA1B knockdown systems in HCC cells.** (A) shRNA-mediated knockdown efficiency of TUBA1B in HCC cells verified by qRT-PCR. (B) Western blot showing TUBA1B protein reduction after shRNA-mediated knockdown; GAPDH served as a loading control. (C) Schematic of inducible TUBA1B-knockdown system. (D) Doxycycline-induced Cas9 expression in HCC-LY10 and MHCC-97H monoclonal lines confirmed by Western blot. (E) Verification of inducible TUBA1B knockdown in Huh7, HCC-LY10 and MHCC-97H cells. (F) Verification of TUBA1B knockdown in Huh7 and HCC-LY10 cells with or without doxycycline. Data are presented as mean ± SD from three independent biological replicates; ns, not significant; **p* < 0.05, ***p* < 0.01, ****p* < 0.001, *****p* < 0.0001. Original blots can be found in Fig. S17-29 to Fig. S17-32.

**Figure S5. TUBA1B depletion suppresses HCC cell proliferation in vitro.** (A) Proliferation of HCC cells following shRNA-mediated TUBA1B knockdown, assessed by Celigo imaging cytometry. (B, C) Representative images and quantification of colony formation assays in HCC cells after TUBA1B knockdown. (D) EdU incorporation assay showing reduced DNA synthesis in HCC cells upon TUBA1B depletion, with corresponding quantification. (E) Proliferation of HCC cells with or without doxycycline treatment, assessed by Celigo imaging cytometry. (F) Representative images of colony formation assays in HCC cells cultured in the presence or absence of doxycycline. (G) Quantification of colony formation in MHCC-97H cells following doxycycline-inducible TUBA1B knockdown. Data are presented as mean ± SD from three independent biological replicates; ns, not significant; **p* < 0.05, ***p* < 0.01, ****p* < 0.001, *****p* < 0.0001.

**Figure S6. TUBA1B depletion suppresses metastatic behaviors and alters F-actin cytoskeletal organization in HCC cells.** (A) Transwell migration and Matrigel invasion assays showing reduced migratory and invasive capacities of HCC cells following TUBA1B knockdown. (B) Wound healing assays confirming impaired cell migration upon TUBA1B depletion. Scale bar, 400 μm. (C) Quantification of cell adhesion to fibronectin- or collagen-coated plates following TUBA1B knockdown. (D) Representative phalloidin staining images showing F-actin organization in shNC and shTUBA1B HCC cells. Quantification of the percentage of stress fiber-positive cells is shown. Cells containing at least three clearly visible linear F-actin bundles across the cell body were defined as stress fiber-positive. Scale bar, 25 μm. Data are presented as mean ± SD from three independent biological replicates; **p* < 0.05, ***p* < 0.01, ****p* < 0.001, *****p* < 0.0001.

**Figure S7. Doxycycline-inducible TUBA1B knockdown suppresses migratory and invasive capacities of HCC cells.** (A, B) Transwell migration and Matrigel invasion assays showing reduced migration and invasion of HCC cells upon doxycycline-induced TUBA1B depletion, with quantification (scale bar, 200 μm). (C, D) Wound healing assays confirming impaired cell migration upon doxycycline-induced TUBA1B depletion, with quantification (scale bar, 400 μm). Data are presented as mean ± SD from three independent biological replicates; **p* < 0.05, ***p* < 0.01, ****p* < 0.001, *****p* < 0.0001.

**Figure S8.** **TUBA1B re-expression rescues HCC cell phenotypes, whereas *Tuba1b* depletion suppresses HCC progression *in vitro* and *in vivo*.** (A) Western blot analysis confirming restoration of TUBA1B protein expression in TUBA1B-knockdown cells. (B, C) Colony formation (B) and Transwell migration/invasion assays (C) demonstrating functional rescue upon TUBA1B re-expression, with quantification. (D) Efficient *Tuba1b* knockdown in Hepa1-6 cells was confirmed. (E–G) *Tuba1b* depletion in Hepa1-6 cells resulted in reduced proliferation (E, F) and impaired migration and invasion (G). (H) Representative gross liver images and liver weight/body weight ratio in the *MYC/sg-p53* HCC model following co-delivery of *PX330* or *PX330-sgTuba1b*. Mice were sacrificed 19 days after HTVi (*n* = 7). (I) Representative H&E staining of liver sections from the indicated *MYC/sg-p53* HCC groups. For in vitro assays, data are presented as mean ± SD from three independent biological replicates. For in vivo experiments, data are presented as mean ± SD; **p* < 0.05, ***p* < 0.01, ****p* < 0.001, *****p* < 0.0001. Original blots can be found in Fig. S17-33 to Fig. S17-34.

**Figure S9.** **Association of TUBA1B with NF-κB signaling and oncogenic pathways.** (A) GSEA of the TCGA-LIHC cohort showing the association between TUBA1B expression and NF-κB signaling. (B) Immunoblot analysis of NF-κB pathway components following TUBA1B knockdown in MHCC-97H cells. (C) H₂O₂ stimulation was used as a positive control to confirm the detectability of phosphorylated IKKα/β and IκBα, which were barely detectable under basal conditions. (D) Nuclear–cytoplasmic fractionation analysis of p65 in MHCC-97H cells. (E-G) Experiments were performed in MHCC-97H, as in Fig.3G-I. (H) Correlation of TUBA1B expression with NF-κB target genes in the TCGA-LIHC cohort. (I–K) GSEA showing enrichment of EMT, MYC activation, and proliferation-related pathways in HCC cells. (L–N) TCGA-LIHC analyses supporting associations with proliferation- and metastasis-related pathways. Data are presented as mean ± SD from three independent biological replicates; **p* < 0.05, ***p* < 0.01, ****p* < 0.001, *****p* < 0.0001. Original blots can be found in Fig. S17-35 to Fig. S17-38.

**Figure S10. Identification of TUBA1B-interacting proteins and functional validation.** (A) SDS-PAGE analysis of proteins pulled down with TUBA1B in Huh7 and MHCC-97H cells. Red arrows indicate the putative TUBA1B bands. (B) IP-MS analysis confirmed efficient TUBA1B pull-down and identified 33 specifically enriched proteins. (C) Molecular docking of 3-dimensional structures shows the interaction of TUBA1B (green) and G3BP2 (blue). (D-G) Experiments were performed in MHCC-97H, as in Fig.4B-E. (H-J) Experiments were performed in MHCC-97H, as in Fig 4I-K. Data are presented as mean ± SD from three independent biological replicates; **p* < 0.05, ***p* < 0.01, ****p* < 0.001, *****p* < 0.0001. Original blots can be found in Fig. S17-39 to Fig. S17-42.

**Figure S11. Functional analysis of TRIM25 and its effect on G3BP2.** (A-D) Experiments were performed in MHCC-97H, as in Fig.5A-D. (E) G3BP2 and TUBA1B protein expression following TRIM25 knockdown. (F) MHCC-97H cells were co-transfected with 1 µg Flag-G3BP2 and increasing amounts of His-TRIM25-WT (0, 0.5, 1.0, 1.5 µg; total DNA equalized with empty vector), and G3BP2 protein levels were analyzed by Western blot. (G) MHCC-97H cells were co-transfected with 1 µg Flag-G3BP2 and increasing amounts of His-TRIM25-ΔRING (0, 0.5, 1.0, 1.5 µg; total DNA equalized with empty vector), and G3BP2 protein levels were analyzed by Western blot. (H) Endogenous interaction between TUBA1B and TRIM25 detected by co-immunoprecipitation. (I) TRIM25 protein expression following TUBA1B depletion. (J-L) Experiments were performed in MHCC-97H, as in Fig 5J-L. Data are presented as mean ± SD from three independent biological replicates; **p* < 0.05, ***p* < 0.01, ****p* < 0.001, *****p* < 0.0001. Original blots can be found in Fig. S17-43 to Fig. S17-54.

**Figure S12. Functional effects of G3BP2 manipulation in HCC cells.** (A) The efficiency of G3BP2 knockdown and overexpression in HCC cells was confirmed. (B-H) Proliferation assessed by Celigo imaging cytometer (B), colony formation assays (C, D), wound healing assay (E, F), and transwell invasion assays (G, H) in Huh7, HCC-LY10, and MHCC-97H cells after G3BP2 knockdown or overexpression. Data are presented as mean ± SD from three independent biological replicates, **p* < 0.05, ***p* < 0.01, ****p* < 0.001, *****p* < 0.0001. Original blots can be found in Fig. S17-55.

**Figure S13. G3BP2 rescues TUBA1B loss-induced defects *in vitro*.** (A-D) Experiments were performed in MHCC-97H, as in Fig 6A-D. (E) Correlation of G3BP2 expression with the NF-κB downstream genes FN1, MYC, VIM, CCND1, SNAI1, and TWIST1 in TCGA-LIHC. (F-J) Experiments were performed in MHCC-97H, as in Fig 6H-L. (K) Representative immunofluorescence images showing p65 and DAPI staining in the indicated cells. Scale bar, 25μm. Quantification of nuclear p65 fluorescence intensity is shown. Data are presented as mean ± SD from three independent biological replicates; **p* < 0.05, ***p* < 0.01, ****p* < 0.001, *****p* < 0.0001. Original blots can be found in Fig. S17-56 to Fig. S17-58.

**Figure S14. Prognostic and mechanistic significance of TUBA1B and G3BP2 in HCC.** (A, B) Validation of G3BP2 upregulation in HCC in LIRI-JP and CHCC-HBV cohorts. (C) Table showing Kaplan-Meier survival times (median and mean) for patients with high expression of TUBA1B, G3BP2, High/High, or the combined High/Low, Low/High, and Low/Low groups. (D) Representative IHC images showing TUBA1B/G3BP2 expression and nuclear p-p65 (S536) positivity. (E) Table showing pairwise and overall *p* values for nuclear p-p65 (S536) positivity among the four TUBA1B/G3BP2 expression groups. (F) Multivariable logistic regression analysis showed that TUBA1B expression was more strongly associated with nuclear p-p65 (Ser536) positivity than G3BP2.

**Figure S15. TUBA1B contributes to cytokine-responsive inflammatory transcription and is associated with immune-related features and immune-cell composition in HCC.** (A, B) RT-qPCR analysis of CCL2/CXCL8 expression in control and TUBA1B-depleted HCC cells left untreated or treated with TNFα (20 ng/mL, 4 h) or IL-6 (20 ng/mL, 4 h). (C) Spearman correlation analysis between TUBA1B expression and the indicated immune- and stroma-related features in the TCGA-LIHC cohort. Dot position indicates the correlation coefficient, dot size represents the magnitude of the correlation, and color represents the corresponding *p* value. (D) Heatmap showing dysfunction, exclusion, CAF, and TIDE scores in TCGA-LIHC tumors stratified into low-, medium-, and high-TUBA1B-expression groups. The lower heatmap shows representative samples from each group. (E) Representative gross liver images and liver-to-body weight ratios of mice with *NRasV12/myr-AKT* -driven liver tumors receiving *PX330* or *sgTuba1b*. (F) Representative H&E staining of liver tumors from the indicated groups. Scale bars are shown in the images. (G) Flow cytometric quantification of the indicated immune-cell populations in the livers of mice with *NRasV12/myr-AKT* -driven tumors receiving *PX330* or sg*Tuba1b*. (H) Flow cytometric quantification of the indicated immune-cell populations in the spleens of mice from the indicated groups. Data are presented as mean ± SD from three independent biological replicates. ns, not significant; **p* < 0.05, ***p* < 0.01, ****p* < 0.001, *****p* < 0.0001.

**Figure S16. Densitometric quantification of key western blot experiments.** Densitometric quantification from three independent biological replicates is shown for the indicated western blot experiments: (A) Fig. 3D, p65 pathway activity; (B) Fig. 3E, nuclear p65 levels; (C) Fig. 3H, NF-κB target proteins; (D) Fig. 4J, G3BP2 abundance; (E) Fig. 5B, MG132-mediated G3BP2 restoration; (F) Fig. 5E, TRIM25-dependent regulation of G3BP2; (G) Fig. 5K, TRIM25 knockdown rescue; (H) Fig. 6H, p65 pathway activity after G3BP2 rescue; (I) Fig. 6I, nuclear p65 accumulation after G3BP2 rescue; and (J) Fig. 6K, NF-κB-related protein expression after G3BP2 rescue. The corresponding representative western blot bands are shown in the indicated main figures. Band intensities were normalized to the appropriate loading controls, fractionation controls, or total protein levels, as indicated in each panel. Data are presented as mean ± SD from three independent biological replicates; ns, not significant; **p* < 0.05, ***p* < 0.01, ****p* < 0.001, *****p* < 0.0001.

**Supplementary Tables**

**Table S1.** **Baseline clinicopathological characteristics stratified by TUBA1B expression (n = 360)**

| **Characteristics** | **TUBA1B expression** | | ***p* value** | **test** |
| --- | --- | --- | --- | --- |
|  | **Low** | **High** |  |  |
| n | 180 | 180 |  |  |
| Pathologic T stage, n (%) |  |  | **0.036** | Chi-square test |
| T1 | 101 (28.1%) | 75 (20.8%) |  |  |
| T2 | 38 (10.6%) | 51 (14.2%) |  |  |
| T3 | 33 (9.2%) | 46 (12.8%) |  |  |
| T4 | 5 (1.4%) | 8 (2.2%) |  |  |
| Pathologic N stage, n (%) |  |  | 0.253 | Fisher’s exact test |
| N0 | 113 (31.4%) | 132 (36.7%) |  |  |
| N1 | 0 (0.0%) | 3 (0.8%) |  |  |
| Pathologic M stage, n (%) |  |  | 0.34 | Fisher’s exact test |
| M0 | 119 (33.1%) | 140 (38.9%) |  |  |
| M1 | 3 (0.8%) | 1 (0.3%) |  |  |
| Pathologic stage, n (%) |  |  | **0.019** | Chi-square test |
| Stage I-II | 132 (39.3%) | 116 (34.5%) |  |  |
| Stage III-IV | 34 (10.1%) | 54 (16.1%) |  |  |
| Gender, n (%) |  |  | 0.91 | Chi-square test |
| Female | 58 (16.1%) | 59 (16.4%) |  |  |
| Male | 122 (33.9%) | 121 (33.6%) |  |  |
| Age, n (%) |  |  | 0.114 | Chi-square test |
| <= 60 | 79 (21.9%) | 94 (26.1%) |  |  |
| > 60 | 101 (28.1%) | 86 (23.9%) |  |  |
| Weight, n (%) |  |  | 0.399 | Chi-square test |
| <= 70 | 85 (23.6%) | 93 (25.8%) |  |  |
| > 70 | 95 (26.4%) | 87 (24.2%) |  |  |
| BMI, n (%) |  |  | 0.461 | Chi-square test |
| <= 25 | 85 (23.6%) | 92 (25.6%) |  |  |
| > 25 | 95 (26.4%) | 88 (24.4%) |  |  |
| Histologic grade, n (%) |  |  | **<0.001** | Chi-square test |
| G1 | 39 (10.8%) | 14 (3.9%) |  |  |
| G2 | 93 (25.8%) | 78 (21.7%) |  |  |
| G3-4 | 46 (12.8%) | 85 (23.6%) |  |  |
| AFP (ng/mL), n (%) |  |  | **<0.001** | Chi-square test |
| <= 400 | 119 (33.1%) | 88 (24.4%) |  |  |
| > 400 | 61 (16.9%) | 92 (25.6%) |  |  |
| Vascular invasion, n (%) |  |  | **0.085** | Chi-square test |
| No | 115 (31.9%) | 85 (23.6%) |  |  |
| Yes | 49 (13.6%) | 55 (15.3%) |  |  |

Note: Percentages were calculated using the total cohort (n = 360) as the denominator, except for pathologic stage, for which percentages were calculated among the 336 cases with available stage information. Missing values are not shown.

**Table S2. Antibodies used in this study**

| **Antibody** | **Catalog** | **Dilution** | **Company** | **Detection** |
| --- | --- | --- | --- | --- |
| TUBA1B | ab108629 | 1:10000/1:2000/1:1000 | abcam | WB/IHC/IF |
| TUBA1B | SAB1404816-100UG | 1:1000 | Sigma-Aldrich | IF |
| TUBA1B | 11224-1-AP | 1ug:1mg | proteintech | Co-IP |
| G3BP2 | 16276-1-AP | 1:1000/1:50/1ug:1mg/1:500 | proteintech | WB/IHC/Co-IP/IF |
| G3BP2 | 31799 | 1:1000/1ug:1mg | CST | WB/Co-IP |
| Cas9 | ab189380 | 1:1000 | abcam | WB |
| TRIM25 | ab167254 | 1:1000/1ug:1mg | abcam | WB/Co-IP |
| IKKβ | 8943 | 1:1000 | CST | WB |
| P-IKKα/β (Ser176/180) | 2694 | 1:1000 | CST | WB |
| IκBα | 4814 | 1:1000/1ug:1mg/1:200 | CST | WB/Co-IP/IF |
| P-IκBα (Ser32/36) | 9246 | 1:1000 | CST | WB |
| P65 | 6956S | 1:1000/1:200 | CST | WB/IF |
| NF-κb P65 | ab32536 | 1:200 | abcam | IHC |
| P-P65 (Ser536) | 3033 | 1:1000 | CST | WB |
| P-P65 (Ser536) | ab86299 | 1:50 | abcam | IHC |
| FN1 | ab268020 | 1:1000/1:200 | abcam | WB/IHC |
| Vimentin | ab92547 | 1:1000/1:100 | abcam | WB/IHC |
| c-myc | 67447-1-Ig | 1:1000/1:100 | proteintech | WB/IHC |
| c-myc | ab32072 | 1:1000 | abcam | WB |
| CyclinD1 | 26939-1-AP | 1:1000/1:100 | proteintech | WB/IHC |
| Histone H3 | 4499S | 1:1000 | CST | WB |
| His | 12698 | 1:1000/1ug:1mg | CST | WB/Co-IP |
| Flag | 14793 | 1:1000/1ug:1mg | CST | WB/Co-IP |
| Flag | F1804 | 1:1000/1ug:1mg | Sigma-Aldrich | WB/Co-IP |
| HA | 3724 | 1:1000/1ug:1mg | CST | WB/Co-IP |
| Ki67 | 9027 | 1:50 | CST | IHC |
| PCNA | 13110 | 1:2000 | CST | IHC |
| GAPDH | MAB374 | 1:1000 | Sigma-Aldrich | WB |
| HRP conjugated goat anti-rabbit IgG | AP106P | 1:5000 | Sigma-Aldrich | WB |
| HRP conjugated goat anti-mouse IgG | AP127P | 1:5000 | Sigma-Aldrich | WB |
| Normal Rabbit IgG Polyclonal Antibody control | 12-370 | 1ug:1mg | Sigma-Aldrich | Co-IP |
| Mouse Anti-rabbit IgG (Conformation Specific) (L27A9) mAb (HRP Conjugate) | 5127S | 1:2000 | CST | WB |
| Goat anti-Mouse IgG (H+L) Highly Cross-Adsorbed Secondary Antibody, Alexa Fluor™ 546 | A11030 | 1:200 | Invitrogen | IF |
| Chicken anti-Rabbit IgG (H+L) Cross-Adsorbed Secondary Antibody, Alexa Fluor™ 488 | A21441 | 1:200 | Invitrogen | IF |
| Donkey anti-Mouse IgG (H+L) Highly Cross-Adsorbed Secondary Antibody, Alexa Fluor™ 488 | A21202 | 1:500 | Invitrogen | IF |
| Goat anti-Rabbit IgG (H+L) Cross-Adsorbed Secondary Antibody, Alexa Fluor™ 568 | A11011 | 1:500 | Invitrogen | IF |

**Table S3. shRNA and sgRNA/gRNA target sequences**

| **Gene** | **Target name** | **Target Sequence (5'-3')** |
| --- | --- | --- |
| TUBA1B | shTUBA1B-1 | GCTGTGTTTGTAGACTTGGAA |
| TUBA1B | shTUBA1B-2 | AGGAGAGGAATACTAATTATC |
| TUBA1B | shTUBA1B-3 | TGACTCCTTCAACACCTTCTT |
| TUBA1B | shTUBA1B-4 | GGAGAGGAATACTAATTATCC |
| G3BP2 | shG3BP2-1 | CCAGAAAGAAAGTTTATGCAA |
| G3BP2 | shG3BP2-2 | CCACAAAGTATTATCTCTGAA |
| TRIM25 | shTRIM25-2 | GAACTGAACCACAAGCTGATA |
| TRIM25 | shTRIM25-3 | CCGGAACAGTTAGTGGATTTA |
| TUBA1B | sgTUBA1B-1 | CGCTGGCAAGCACGTGCCCC |
| TUBA1B | sgTUBA1B-2 | TTCAGAGGCCCGTGAAGATA |
| Tuba1b | gRNA-Tuba1b-Ex2 | AGGATGGAAGAGCTGGCGGT |
| Tuba1b | gRNA-Tuba1b-Ex3 | CCTGTGCACTGGTCAGCCTG |

**Table S4. The information of plasmids from Addgene**

| pCW-Cas9 | pCW-Cas9 was a gift from Eric Lander & David Sabatini (Addgene plasmid # 50661; http://n2t.net/addgene:50661; RRID:Addgene_50661) |
| --- | --- |
| pRK5-HA-Ub-K48 | pRK5-HA-Ubiquitin-K48 was a gift from Ted Dawson (Addgene plasmid # 17605; http://n2t.net/addgene:17605; RRID:Addgene_17605) |
| pRK5-HA-Ub-K63 | pRK5-HA-Ubiquitin-K63 was a gift from Ted Dawson (Addgene plasmid # 17606; http://n2t.net/addgene:17606; RRID:Addgene_17606) |
| pRK5-HA-Ub | pRK5-HA-Ubiquitin-WT was a gift from Ted Dawson (Addgene plasmid # 17608; http://n2t.net/addgene:17608; RRID:Addgene_17608) |
| pT-CAG-NRAS(V12) | pT/Caggs-NRASV12 was a gift from John Ohlfest (Addgene plasmid # 20205; http://n2t.net/addgene:20205; RRID:Addgene_20205) |
| pT3-myr-AKT | pT3-myr-AKT-HA was a gift from Xin Chen (Addgene plasmid # 31789; http://n2t.net/addgene:31789; RRID:Addgene_31789) |
| pCMV（CAT）T7-SB100 | pCMV(CAT)T7-SB100 was a gift from Zsuzsanna Izsvak (Addgene plasmid # 34879 ; http://n2t.net/addgene:34879 ; RRID:Addgene_34879) |
| psPAX2 | psPAX2 was a gift from Didier Trono (Addgene plasmid # 12260; http://n2t.net/addgene:12260; RRID:Addgene_12260) |
| pMD2.G | pMD2.G was a gift from Didier Trono (Addgene plasmid # 12259; http://n2t.net/addgene:12259; RRID:Addgene_12259) |

**Table S5. Primer sequences**

| **Gene name** | **Sequence (5'-3')** |
| --- | --- |
| TUBA1B-F | GAGGAGATGACTCCTTCAACACC |
| TUBA1B-R | TGATGAGCTGCTCAGGGTGGAA |
| G3BP2-F | GAGCTGAAACCACAAGTGGAGG |
| G3BP2-R | GGTCACTGAAGCCCAGGAGAAA |
| FN1-F | ACAACACCGAGGTGACTGAGAC |
| FN1-R | GGACACAACGATGCTTCCTGAG |
| VIM-F | AGGCAAAGCAGGAGTCCACTGA |
| VIM-R | ATCTGGCGTTCCAGGGACTCAT |
| C-MYC-F | CCTGGTGCTCCATGAGGAGAC |
| C-MYC-R | CAGACTCTGACCTTTTGCCAGG |
| CCND1-F | TCTACACCGACAACTCCATCCG |
| CCND1-R | TCTGGCATTTTGGAGAGGAAGTG |
| GAPDH-F | GTCTCCTCTGACTTCAACAGCG |
| GAPDH-R | ACCACCCTGTTGCTGTAGCCAA |

**Table S6. Genetically defined mouse HCC models included in GSE148379**

| **Reported mouse model** | **Major genetic drivers / perturbations inferred from model name** |
| --- | --- |
| MYC;sg-p53 | MYC overexpression with p53/Trp53 perturbation |
| MYC;sg-Kmt2b | MYC overexpression with Kmt2b perturbation |
| MYC;sg-Kmt2c | MYC overexpression with Kmt2c perturbation |
| MYC;sg-Pten | MYC overexpression with Pten perturbation |
| MYC;CTNNB1 | MYC overexpression with CTNNB1/β-catenin activation |
| MYC;sg-Axin1 | MYC overexpression with Axin1 perturbation |
| CTNNB1;sg-Pten | CTNNB1/β-catenin activation with Pten perturbation |
| CTNNB1;Tert | CTNNB1/β-catenin activation with Tert expression |
| MYC;Tert | MYC overexpression with Tert expression |
| myc-luc-bcat | MYC-luciferase reporter-associated β-catenin model |
| myc-lucOS-bcat | MYC-luciferase reporter-associated β-catenin model |

Note: This table summarizes the reported mouse HCC model groups analyzed from GSE148379 based on the dataset annotation and model names. These models provide cross-species supportive evidence across selected genetically defined oncogenic contexts, but they do not comprehensively represent the etiological, immune, stromal, or molecular subtype heterogeneity of human HCC.

**Table S7. Baseline clinicopathological characteristics of**

**the institutional IHC cohort**

| **Characteristic** | **Institutional IHC cohort (n = 199)** |
| --- | --- |
| Age, years, median (range) | 50 (25–83) |
| Male sex, n (%) | 160/199 (80.4%) |
| Cirrhosis, n (%) | 168/199 (84.4%) |
| HBsAg positive, n (%) | 158/193 (81.9%) |
| HBeAg positive, n (%) | 32/186 (17.2%) |
| anti-HBc positive, n (%) | 145/186 (78.0%) |
| anti-HCV positive, n (%) | 8/86 (9.3%) |
| AFP, ng/mL, median (range) | 50 (0–158526) |
| AFP >400 ng/mL, n (%) | 51/196 (26.0%) |
| Tumor size, cm, median (range) | 5.3 (1.5–20) |
| Tumor size >5 cm, n (%) | 101/195 (51.8%) |
| Lymph-node metastasis, n (%) | 5/199 (2.5%) |
| Intrahepatic metastasis, n (%) | 62/199 (31.2%) |
| Vein invasion, n (%) | 33/198 (16.7%) |
| Vascular invasion, n (%) | 15/153 (9.8%) |
| Histological grade, I / II / III / IV | 10 / 87 / 55 / 47 |
| Follow-up time, days, median (range) | 757 (2–3149) |
| Molecular subclass | Not available |

**Note**: Data are shown as n/available n (%) unless otherwise indicated. Cases with missing information were excluded from the corresponding summary. Molecular subclass information was not available because matched transcriptomic profiling was not performed for this institutional IHC cohort.
